# Endoribonuclease-mediated control of *hns* mRNA stability constitutes a key regulatory pathway for *Salmonella* Typhimurium pathogenicity island 1 expression

**DOI:** 10.1371/journal.ppat.1009263

**Published:** 2021-02-01

**Authors:** Minho Lee, Minkyung Ryu, Minju Joo, Young-Jin Seo, Jaejin Lee, Hong-Man Kim, Eunkyoung Shin, Ji-Hyun Yeom, Yong-Hak Kim, Jeehyeon Bae, Kangseok Lee

**Affiliations:** 1 Department of Life Science, Chung-Ang University, Dongjak-Gu, Seoul, Republic of Korea; 2 Department of Pharmacy, Chung-Ang University, Dongjak-Gu, Seoul, Republic of Korea; 3 Department of Microbiology, Daegu Catholic University School of Medicine, Daegu, Republic of Korea; University of Toronto, CANADA

## Abstract

Bacteria utilize endoribonuclease-mediated RNA processing and decay to rapidly adapt to environmental changes. Here, we report that the modulation of *hns* mRNA stability by the endoribonuclease RNase G plays a key role in *Salmonella* Typhimurium pathogenicity. We found that RNase G determines the half-life of *hns* mRNA by cleaving its 5′ untranslated region and that altering its cleavage sites by genome editing stabilizes *hns* mRNA, thus decreasing *S*. Typhimurium virulence in mice. Under anaerobic conditions, the FNR-mediated transcriptional repression of *rnc* encoding RNase III, which degrades *rng* mRNA, and simultaneous induction of *rng* transcription resulted in rapid *hns* mRNA degradation, leading to the derepression of genes involved in the *Salmonella* pathogenicity island 1 (SPI-1) type III secretion system (T3SS). Together, our findings show that RNase III and RNase G levels-mediated control of *hns* mRNA abundance acts as a regulatory pathway upstream of a complex feed-forward loop for SPI-1 expression.

## Author summary

Recent studies have shown that pathogenic bacteria with ribonuclease mutations display attenuated virulence, impaired mobility, and reduced proliferation in host cells. However, the molecular mechanisms underlying ribonuclease-associated pathogenesis have not yet been characterised. Here, we provide strong experimental evidence that the coordinated modulation of endoribonuclease activity constitutes an additional regulatory layer upstream of a complex feed-forward loop controlling global regulatory systems in the *Salmonella* pathogenicity island 1 (SPI-1) type III secretion system (T3SS). In addition, we showed that this regulatory pathway plays a key role in the virulence of *S*. Typhimurium in the host. Thus, our study improves the understanding of the mechanisms through which bacterial pathogens sense the host environment and respond precisely by expressing gene products required for adaptation to that particular niche.

## Introduction

Bacterial ribonucleases regulate gene expression via RNA processing and decay. They are essential for processing RNA precursors into mature, functional RNAs, and initiate the reactions that cleave and differentially regulate certain polycistronic mRNAs [1–5]. Bacterial ribonucleases also degrade mRNAs in response to environmental changes, such as nutrient starvation, exposure to toxic compounds, and changes in physicochemical factors such as temperature, pH, oxygen, pressure, redox-state, and salt concentration [2–7].

In *Escherichia coli*, mRNA decay is heavily dependent on endoribonuclease E (RNase E) [8–10], whose paralog endoribonuclease G (RNase G; encoded by *rng*) has a 36% amino acid sequence identity with the RNase E N-terminal catalytic domain [11,12]. Both enzymes are single-stranded RNA-specific endoribonucleases with a preference for cleaving AU-rich sequences [11,13,14]. RNase G participates in rRNA maturation and mRNA degradation [9,11,15–19]. *E*. *coli* lacking *rne* can be made viable by overexpressing RNase G; however, *rng*-complementation affects the abundance of a small portion of mRNAs in *rne*-deficient cells [9], indicating that RNase G and E play distinct physiological roles.

*Salmonella enterica* serovar Typhimurium (*Salmonella* Typhimurium) is a gram-negative, facultative intracellular bacterial pathogen of animals and humans. Since its RNase E and G homologs are evolutionarily close to those found in *E*. *coli* [20], *S*. Typhimurium is an excellent model system for studying the effect of RNA processing and degradation on bacterial pathogenicity. *S*. Typhimurium contains chromosomal regions, known as *Salmonella* pathogenicity islands (SPIs), that encode virulence factors. The SPI-1 region contains 39 genes encoding a type III secretion system (T3SS) that is essential for host cell invasion [21]. SPI-1 and other SPI genes are required for different stages of *Salmonella* pathogenesis [22–24], and the expression of SPI-1 invasion and effector genes can change in response to multiple environmental signals, decreasing under high oxygen, low osmolarity, low pH, and stationary phase conditions [23,25,26].

Coordinated signalling systems regulate SPI-1 gene expression via the transcription factors HilA, HilC, and HilD, encoded by SPI-1 [27,28], which are regulated upstream by histone-like nucleoid-structuring protein (H-NS). H-NS is a nucleoid-associated global transcriptional silencer abundant in enteric bacteria and represses AT-rich gene expression to protect the bacterial genome from horizontally acquired sequences [29,30]. H-NS regulates SPI-1 expression by binding to and down-regulating the *hilA* promoter [31], and is thought to form a complex with Hha, a small nucleoid-associated protein encoded by the *hly* operon in *E*. *coli* [32,33]; however, *S*. Typhimurium H-NS may bind the *hilA* promoter independently of Hha [31,34,35].

mRNA stability modulation has been associated with the ability of *S*. Typhimurium to rapidly adapt to host environments [36]. For instance, *S*. Typhimurium with RNase E and RNase III mutations display attenuated virulence, impaired mobility, and reduced proliferation inside *Galleria mellonella* and mice [37]. RNase III and polynucleotide phosphorylase (PNPase) are involved in the regulation of small regulatory RNAs in *S*. Typhimurium [38,39], and we previously showed that RNase G affects *S*. Typhimurium aminoglycoside resistance by processing the 5′ end of 16S rRNA [19]; however, the molecular mechanisms underlying ribonuclease-mediated *S*. Typhimurium pathogenesis have not yet been elucidated.

In this study, we observed that *S*. Typhimurium pathogenicity is associated with RNase G expression and further investigated the molecular mechanism underlying the pathophysiological role of RNase G in SPI-1 T3SS expression.

## Results

### Effect of RNase G on *S*. Typhimurium pathogenicity

**Fig 1:**
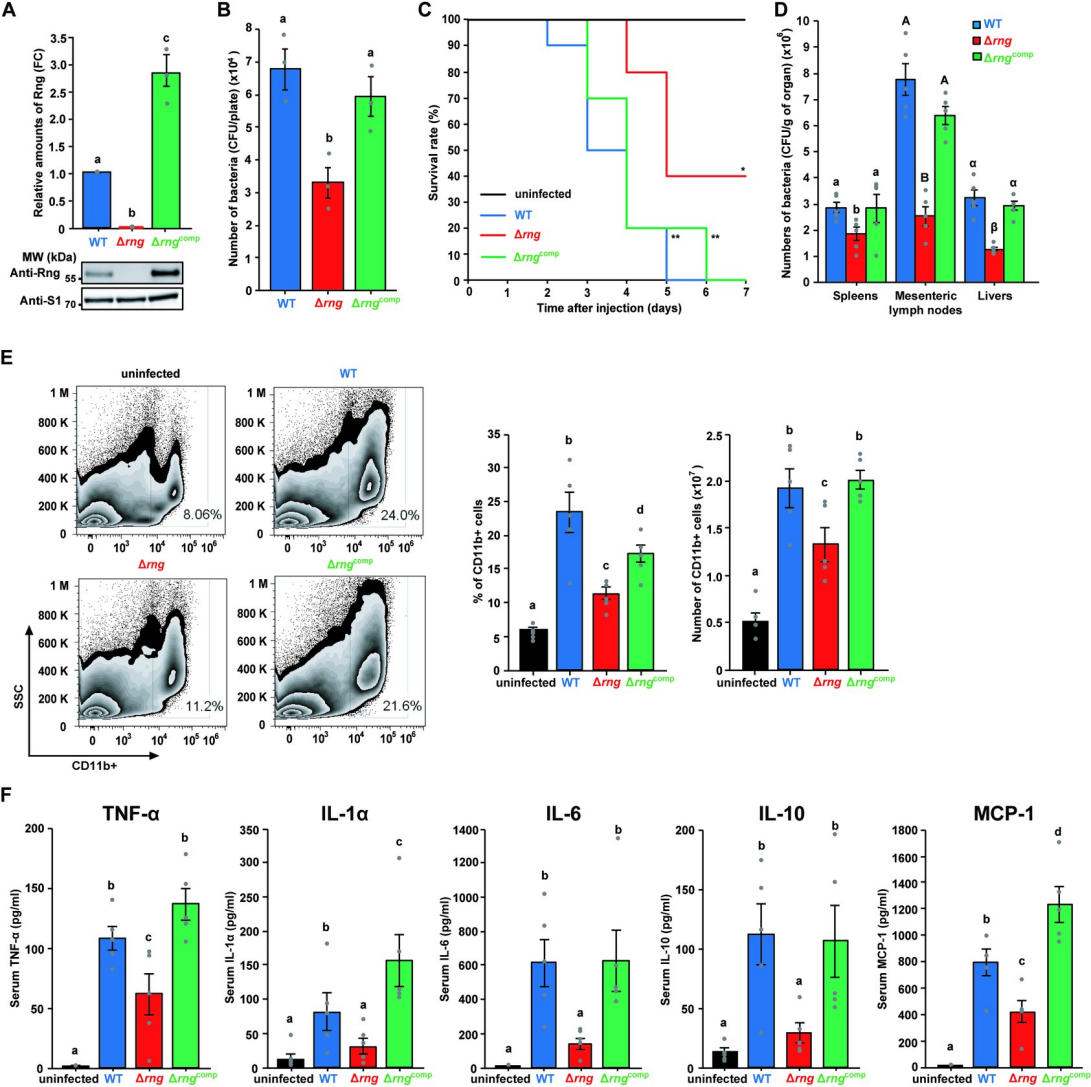
Effect of RNase G on *S*. Typhimurium pathogenicity. **(A)** Western blot analysis of Rng in *S*. Typhimurium strains (WT, Δ*rng*, and Δ*rng*^comp^). WT Rng levels were set to 1 and ribosomal protein S1 was used as an internal standard. **(B)** Effect of *rng* expression on *S*. Typhimurium epithelial cell invasion. Epithelial cells (HCT116) were infected with the *S*. Typhimurium cultures used in (A) at a multiplicity of infection (MOI) of 100 and the number of intracellular bacteria counted as CFUs. For **(A)** and **(B)**, data present the mean ± s. e. m. of at least three independent experiments. Statistically significant differences are indicated by different letters. Western blot data were analyzed by one-way analysis of variance (ANOVA) with Student-Newman-Keuls test, *P* < 0.0001. Epithelial cell invasion data were analyzed using the one-way ANOVA with Student-Newman-Keuls test, *P* < 0.05. **(C)** Survival rate and duration of BALB/c mice infected with *S*. Typhimurium strains (WT, Δ*rng*, and Δ*rng*^comp^) and PBS as a control. Survival was monitored for 7 days (*n* = 10 per group). * *P* < 0.05 and ** *P* < 0.01 for WT-, Δ*rng*-, or Δ*rng*^comp^-infected mice vs. PBS-treated mice (two-sided unpaired Student’s *t*-test). **(D)** Colonization assay in BALB/c mice (*n* = 5 per group) infected with *S*. Typhimurium strains (WT, Δ*rng*, and Δ*rng*^comp^). Bacterial CFUs were counted in the spleens, livers, and mesenteric lymph nodes. Data represent the mean ± s. e. m. of at least three independent experiments. Statistically significant differences by one-way ANOVA with Student-Newman-Keuls tests (*P* < 0.0001) are indicated by different letters: small letters indicate a difference from the spleens; large letters indicate a difference from the mesenteric lymph nodes; Greek symbols indicate a difference from the livers. **(E)** Flow cytometry analysis of innate myeloid immune cells (CD11b+) in mouse splenocytes from BALB/c mice infected with or without *S*. Typhimurium strains (WT, Δ*rng*, or Δ*rng*^comp^). Cell numbers were calculated from total splenocyte counts. **(F)** Serum inflammatory cytokine (TNF-α, IL-1α, IL-6, IL-10, and MCP-1) levels in BALB/c mice infected with or without *S*. Typhimurium strains (WT, Δ*rng*, or Δ*rng*^comp^) for 60 h. Data represent the mean ± s. e. m. of at least two independent experiments. Statistically significant differences from one-way ANOVA with Student-Newman-Keuls tests (*P* < 0.0001 for TNF-α and MCP-1; *P* < 0.01 for IL-1α, IL-6, and IL-10) are indicated by different letters.

First, we examined whether RNase G levels affected *S*. Typhimurium pathogenicity using an epithelial cell infection assay. This method is a good model system for examining *S*. Typhimurium virulence since mutants with defective invasion and persistence in epithelial cells display markedly reduced virulence in animal hosts [40,41]. To assess the effect of RNase G levels on bacterial virulence, we measured the colony forming units (CFUs) of wild-type (WT; SL1344 + pACYC177), *rng*-deleted (Δ*rng*; SL1344*rng* + pACYC177), and *rng*-complemented (Δ*rng*^comp^; SL1344*rng* + pSt-rng) *S*. Typhimurium strains following the infection of human intestinal epithelial cells (HCT116; Fig 1A). We found that the Δ*rng* strain produced 51% fewer CFUs in HCT116 cells than the WT, whereas the Δ*rng*^comp^ strain displayed a comparable number of intracellular CFUs to the WT strain (Fig 1B).

Next, we investigated RNase G level-associated *S*. Typhimurium pathogenicity in a mouse model infected with *S*. Typhimurium at 10^4^ CFUs (S1 Fig). While mice infected with either WT or Δ*rng*^comp^ strain exhibited a similar mortality rate (all succumbed to the infection within 7 days), 40% of the mice infected with the Δ*rng* strain survived 7 days post-infection (Fig 1C). In addition, we assessed bacterial load in the spleens, livers, and mesenteric lymph nodes of the infected mice. There were significantly fewer Δ*rng* cells than WT cells in all the three organs. Furthermore, this colonization defect was restored to WT levels by complementation with the *rng* gene (Δ*rng*^comp^; Fig 1D).

Since the spleen plays key roles in the clearance of invading pathogens and the generation of specific immune responses, we assessed whether RNase G levels in *S*. Typhimurium affect host immune responses against bacterial infection by analysing the proportion of innate myeloid immune cells (CD11b+) in spleens by flow cytometry (Fig 1E). Mice infected with the WT strain displayed a higher percentage (uninfected: 5.9%, WT-infected: 23.4%) and number (uninfected: 0.5 × 10^7^, WT-infected: 1.9 × 10^7^) of CD11b+ cells than those infected with Δ*rng* cells (11.3% and 1.3 × 10^7^) and this reduction was restored in mice infected with the Δ*rng*^comp^ strain (17.3% and 2.0 × 10^7^; Fig 1E).

These results were confirmed by measuring serum levels of inflammatory cytokines, including IL-1α, IL-1β, IL-6, IL-10, IL-12p70, IL-17A, IL-23, IL-27, MCP-1, IFN-β, TNF-α, and GM-CSF. While IL-1β, IL-12p70, IL-17A, IL-23, IL-27, IFN-β, and GM-CSF levels did not change significantly in Δ*rng*-infected mice (S2 Fig), WT infection sharply increased the levels of TNF-α, IL-1α, IL-6, IL-10, and MCP-1 in the serum of mice (Fig 1F), indicating that RNase G plays a pathogenic role in inflammation induced by *S*. Typhimurium infection. Consistent with our previous results, mice infected with the Δ*rng*^comp^ strain displayed inflammatory cytokine levels comparable to those of the WT-infected mice.

Cytokines released from activated innate immune cells play essential roles as mediators of inflammation and in the elimination of invading pathogens [42]; however, the excessive expression of inflammatory cytokines such as TNF-α, IL-1α, and IL-6 is directly involved in bacterial pathogenicity, leading to the death of infected hosts [43–45]. Our data revealed that the mortality rate of infected mice correlated positively with the levels of inflammatory cytokines, including TNF-α, IL-1α, and IL-6 (Fig 1F). Thus, inflammatory cytokine expression was lower in the RNase G-deficient strain than in the RNase G-sufficient strain and may explain the reduced mortality of mice infected with the Δ*rng* strain. Taken together, these results suggest that RNase G levels are strongly associated with *S*. Typhimurium pathogenicity.

### Effect of RNase G levels on *S*. Typhimurium protein profiles

**Fig 2:**
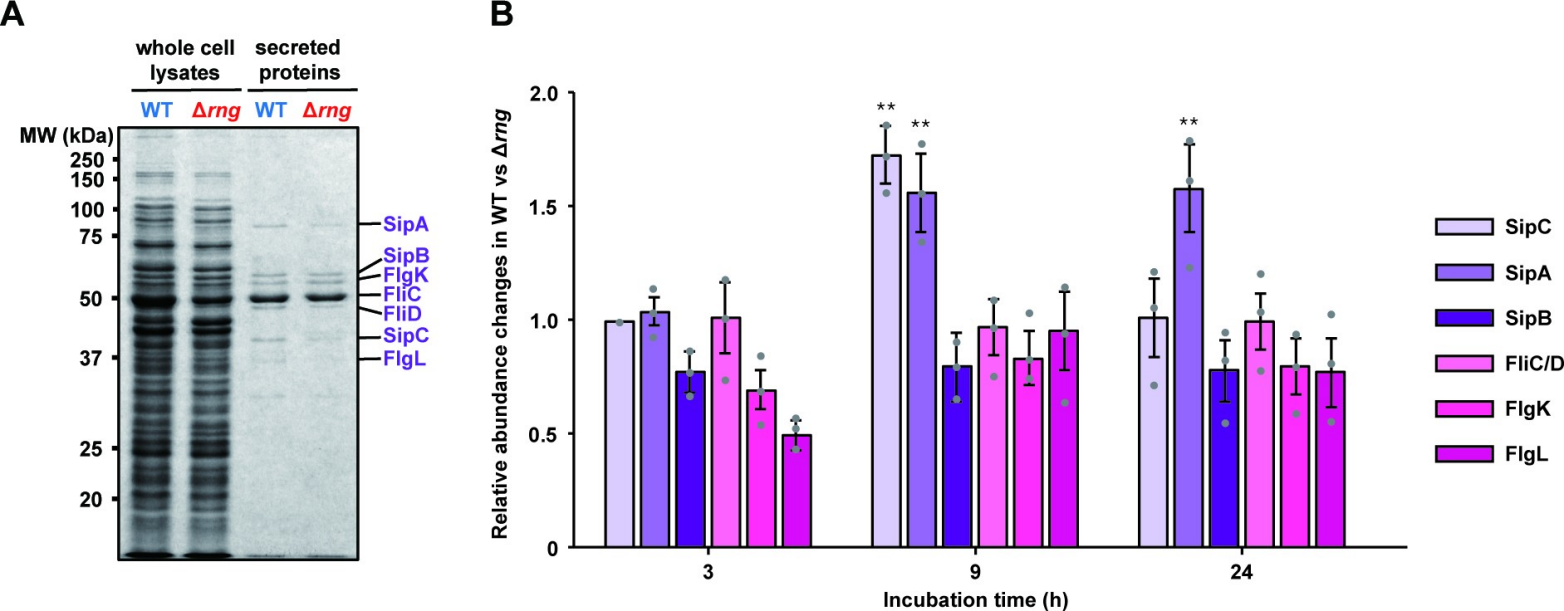
Differentially expressed proteins in WT and Δ*rng* strains. **(A)** SDS-PAGE analysis of T3SS effector proteins and flagellar proteins in the whole-cell lysates and concentrated supernatants of *S*. Typhimurium strains (WT and Δ*rng*). Seven specific protein bands (SipA, SipB, FlgK, FliC, FliD, SipC, and FlgL denoted on the right of the gel) were identified by in-gel digestion and tandem mass spectrometry, as described in the Materials and Methods. **(B)** The relative abundance of T3SS effector proteins and flagellar proteins determined from the protein expression profiles in the supernatants of WT and Δ*rng* strains, as described in S3 Fig. Relative abundance of these proteins was measured by densitometric analysis of the band intensities of these proteins. For this analysis, the relative abundance of FliC/FliD proteins was set to 1. Statistically significant differences in relative SipA and SipC levels between WT and Δ*rng* cells were compared to the FliC/FliD levels by two-tailed *t*-tests. ** *P* < 0.01.

The proteome of *S*. Typhimurium cells is known to change dynamically throughout adhesion, colonization, invasion, intracellular survival, proliferation, and biofilm formation under the control of SPI gene expression [46,47]. To understand the effect of RNase G on T3SS expression, we examined the protein expression profiles in supernatants of the WT and Δ*rng* strains by SDS-PAGE (S3 Fig) and the major protein bands between 30 to 90 kDa by tandem mass spectrometry. The two strains displayed overall similar growth rates and protein secretion levels during cultivation in Luria-Bertani (LB) medium with aeration (S3A Fig) except that the WT strain was able to secrete higher levels of the T3SS effector proteins SipA and SipC during early-stationary (or late-exponential) growth phase (9 h incubation), compared to the Δ*rng* strain (Figs 2 and S3B). The mass spectrometry results showed that the secretion of SlrP and SopA was also higher in the WT than in the Δ*rng* strain (Table 1). We also observed that the secretion levels of several cytoplasmic proteins (FusA, GlpK, LpdA, PflB, and Pta) in the WT strain were relatively lower compared to the major T3SS effector proteins (Table 1). Further studies are needed to explain this phenomenon and, in this study, we focused on the change of SPI-related genes expression to understand the effects of RNase G levels on the pathogenicity of *S*. Typhimurium.

**Table 1:** Normalized spectral abundance factors and fold changes in proteins identified by tandem mass spectrometry analysis of in-gel digested bands from the supernatants of WT and Δ*rng Salmonella* Typhimurium.

Accession No.	Description (protein name)	Protein	Molecular	PSM No.		NASF		Fold
		length (a. a)	Mass (kDa)	WT	Δ*rng*	WT	Δ*rng*	WT/Δ*rng*
CBW16874.1	T3SS effector protein (SlrP)	765	86.8	8	4	0.003	0.002	1.776
CBW18139.1	T3SS effector protein (SopA)	782	86.7	14	7	0.005	0.003	1.776
CBW17217.1	Flagellar hook-associated protein 3 (FlgL)	317	34.2	3	2	0.003	0.002	1.332
CBW16256.1	Dihydrolipoamide acetyltransferase component E2 (AceF)	629	66.0	7	5	0.003	0.003	1.243
CBW17984.1	Flagellar hook associated protein (FliD)	467	49.8	208	152	0.128	0.106	1.215
CBW18959.1	SPI-1 T3SS effector protein (SipA)	685	73.9	602	458	0.253	0.217	1.167
CBW18961.1	SPI-1 T3SS effector protein (SipC)	409	43.0	6	5	0.004	0.004	1.066
CBW18855.1	Flagellin (FljB)	506	52.5	388	356	0.221	0.229	0.968
CBW17983.1	Flagellin (FliC)	495	51.6	605	565	0.353	0.371	0.951
CBW18962.1	SPI-1 T3SS effector protein (SipB)	593	62.4	10	10	0.005	0.005	0.888
CBW17216.1	Flagellar hook-associated protein 1 (FlgK)	553	59.1	5	5	0.003	0.003	0.888
CBW19351.1	Polynucleotide phosphorylase (Pnp)	711	77.0	8	10	0.003	0.005	0.710
CBW19923.1	ATP synthase beta subunit (AtpD)	460	50.3	4	5	0.003	0.004	0.710
CBW19508.1	Elongation factor G (FusA)	704	77.6	5	9	0.002	0.004	0.493
CBW20124.1	Glycerol kinase (GlpK)	502	56.0	7	13	0.004	0.008	0.478
CBW16257.1	Dihydrolipoamide dehydrogenase (LpdA)	474	50.6	5	10	0.003	0.007	0.444
CBW17006.1	Formate acetyltransferase 1 (PflB)	760	85.0	4	13	0.002	0.006	0.273
CBW18409.1	Phosphate acetyltransferase (Pta)	714	77.2	2	9	0.001	0.004	0.197

*N*.*b*. Mass spectrometric data combines all the gel slices obtained from the supernatants of WT and Δ*rng S*. Typhimurium at different cultivation times of 3 h, 9 h, 24 h, and 48 h, as shown in S3 Fig. PSM (peptide spectrum match) numbers and NSAF (normalized spectral abundance factor) of each protein identified by filtering the tandem mass spectrometric data with two or more unique peptides are shown in S1 and S2 Tables.

SPI-1 T3SS induction in aerobically grown late-exponential phase *Salmonella* is known to export effector proteins into the medium or host cells that are necessary for invasion, stimulating inflammation, and virulence [48,49]. During *Salmonella* pathogenesis, increased SipA and SipC secretion in the supernatant of WT cells can aid invasion by facilitating the trafficking of the mammalian tetraspanning membrane protein PERP at the apical surface of host cells [50] and remodelling its actin cytoskeleton [51]. The two strains displayed no significant changes in normalized spectral abundance factors (NSAFs) or flagellar proteins, such as FlgK, FlgL, FljB, FliC, and FliD. Similar results have been reported for salt-activated expression in the *Salmonella* T3SS without altering flagellar protein secretion by increasing the transcription of *hilA*, *hilC*, and *hilD* [52]. Together, our findings suggest that WT *S*. Typhimurium can secrete more SPI-1 T3SS effector proteins than the Δ*rng* strain.

### Effect of RNase G levels on T3SS activity

**Fig 3:**
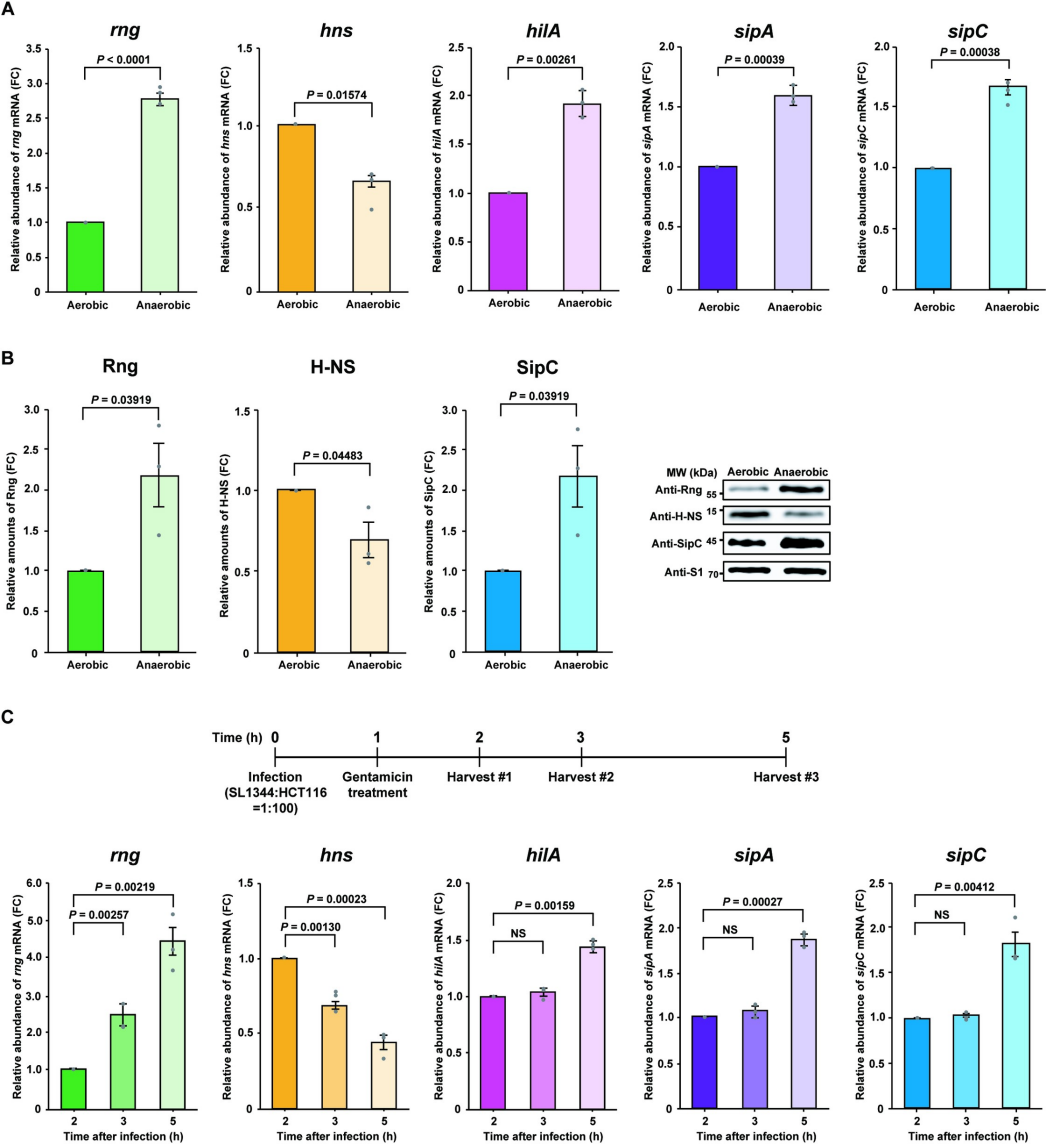
Effect of RNase G on SPI-1 T3SS. **(A)** Analysis of *rng*, *hns*, and SPI-1-related gene (*hilA*, *sipA*, and *sipC*) mRNA expression in WT *S*. Typhimurium under aerobic and anaerobic conditions by qRT-PCR. Expression levels of *rng*, *hns*, *hilA*, *sipA*, and *sipC* mRNA under aerobic conditions were set to 1. **(B)** Western blot analysis of Rng, H-NS, and SipC in the *S*. Typhimurium cultures used in (A). WT Rng, H-NS, and SipC levels under aerobic conditions were set to 1. Ribosomal protein S1 was used as an internal standard. **(C)** Analysis of *rng*, *hns*, and SPI-1-related gene (*hilA*, *sipA*, and *sipC*) mRNA expression in the infection stage by qRT-PCR. HCT116 cells were infected with WT *S*. Typhimurium at a multiplicity of infection (MOI) of 100. *rng*, *hns*, *hilA*, *sipA*, and *sipC* mRNA expression levels at 2 h after infection were set at 1. For **(A)** and **(C)**, *rng*, *hns*, *hilA*, *sipA*, and *sipC* mRNA expression levels were normalized to *ribE* mRNA and gene expression was quantified using the ΔΔCt method. For **(A)**, **(B)**, and **(C)**, data represent the mean ± s. e. m. of three independent experiments. Statistically significant values from two-sided unpaired Student’s *t*-tests are indicated. NS; not significant.

RNase G is a bacterial endoribonuclease known to negatively regulate gene expression via mRNA decay [9,15–17]; however, SDS-PAGE and mass spectrometry revealed that some SPI-1 T3SS effectors (SlrP, SopA, SipA, and SipC) were up-regulated in the WT strain compared to that in the Δ*rng* strain, indicating that changes in their mRNA levels were not a direct consequence of mRNA decay by RNase G. To assess this further, we analyzed upstream pathways to identify a SPI-1 T3SS repressor that can be negatively regulated by RNase G. When *S*. Typhimurium invades host cells or responds to elevated sucrose or salt concentrations, SPI-1 T3SS effector gene expression is activated by the major transcriptional activator HilA, which is generally repressed by H-NS [31,35,52]. To explore the relationships between *sipA*, *sipC*, *hilA*, and *hns* mRNA expression levels, real-time reverse transcription polymerase chain reaction (qRT-PCR) was performed using total RNA from WT cells cultured under aerobic and anaerobic conditions, since SPI-1 T3SS expression is known to be up-regulated during *Salmonella* growth under low oxygen conditions and during host cell infection [27,53]. Expression levels of *sipA*, *sipC*, *hilA*, and *hns* gene changed dynamically in WT *S*. Typhimurium cells in response to anaerobic conditions. Steady-state *sipA*, *sipC*, and *hilA* mRNA expression increased by approximately 1.6-, 1.7-, and 1.9-fold, respectively, compared to cells grown under aerobic conditions, whereas *hns* mRNA abundance decreased by ~ 40% under anaerobic conditions (Fig 3A). In addition, the relative abundance of *rng* mRNA increased by ~ 2.8-fold under anaerobic conditions (Fig 3A). Changes in RNase G, H-NS, and SipC protein levels following changes in oxygen availability correlated with their mRNA abundance (Fig 3B), indicating that RNase G is associated with the positive and negative regulation of SPI-1-related gene expression by HilA and H-NS, respectively.

Next, we investigated the association between the expression of RNase G and that of virulence factors in WT *S*. Typhimurium cells infecting epithelial cells. The differential expression of SPI-1-associated genes was monitored by qRT-PCR in total RNA samples from WT *S*. Typhimurium cells 2, 3, and 5 h after epithelial cell infection. The relative abundance of *sipA*, *sipC*, *hilA*, and *rng* mRNA gradually increased as *hns* mRNA levels decreased during infection (Fig 3C); therefore, we hypothesized that increased RNase G levels downregulate *hns* transcript expression in *S*. Typhimurium infecting host cells or under anaerobic conditions, thus enhancing SPI-1 T3SS gene expression.

### Effect of RNase G levels on *hns* mRNA stability

**Fig 4:**
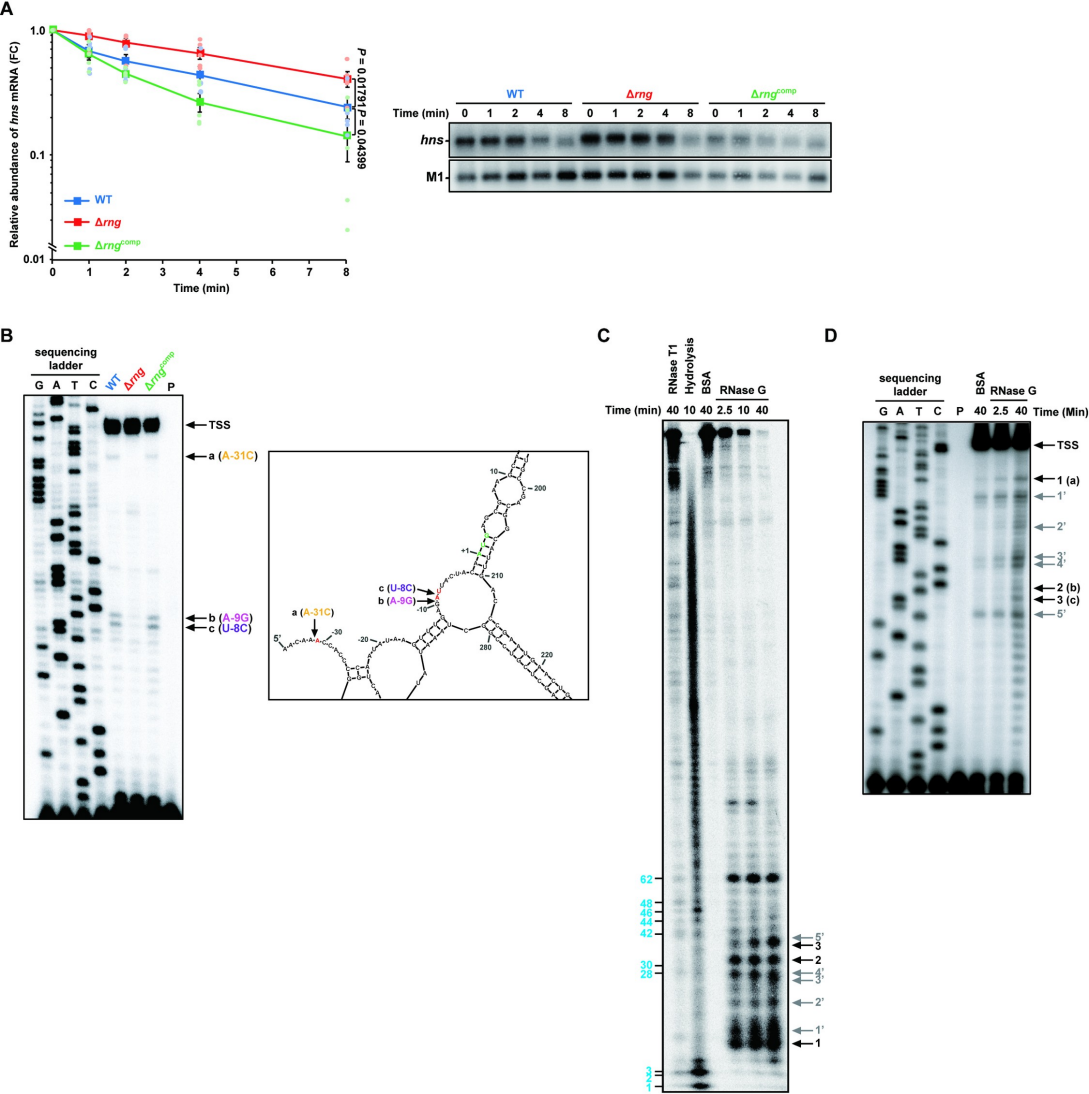
Down-regulation of *hns* mRNA abundance by RNase G. **(A)** Effect of cellular RNase G on *hns* mRNA decay in *S*. Typhimurium strains (WT, Δ*rng*, and Δ*rng*^comp^) grown under anaerobic conditions, as measured by northern blot analysis. Data represent the mean ± s. e. m. of three independent experiments and presented on a semi-logarithmic plot. Statistically significant values from two-sided unpaired Student’s *t*-tests are indicated. **(B)** Primer extension analysis of *hns* mRNA 5′ UTR *in vivo*. Left: Total RNA samples isolated from the *S*. Typhimurium cultures used in (A) were analyzed. Sequencing ladders were synthesized with the same primer used in cDNA synthesis and PCR DNA encompassing the *hns* gene as a template. Right: The secondary structure of the 5′ UTR of *hns* mRNA deduced from the M-Fold program. Arrows indicate RNase G cleavage sites. The start codon is indicated by bold, green characters. TSS; transcription start site. **(C)** *In vitro* cleavage of *hns* mRNA. RNase T1 cleavage products are indicated by blue bold characters. Black arrows indicate the cleavage sites identified in (B) (1, 2, and 3). **(D)** Primer extension analysis of the *in vitro*-cleaved *hns* mRNA products purified from (C). Black arrows indicate the cleavage sites identified in (B) and (C) (1 = a, 2 = b, and 3 = c). For **(C)** and **(D)**, grey arrows indicate (1′-5′) nonspecific RNase G cleavage sites.

To determine whether *hns* mRNA abundance is directly regulated by RNase G, we determined the decay rate of *hns* mRNA in WT, Δ*rng*, and Δ*rng*^comp^ strains under anaerobic conditions. Northern blot analysis revealed that the half-life of *hns* mRNA was ~ 2.6-fold higher in Δ*rng* cells than in WT cells (7.6 min vs. 3.0 min; Fig 4A), whereas the Δ*rng*^comp^ strain rapidly degraded *hns* mRNA with a half-life of 1.7 min. These experimental results suggest that *hns* mRNA stability is greatly influenced by RNase G levels.

Since RNase E/G family proteins are known to influence mRNA half-life by cleaving the 5′ untranslated region (UTR) of most biochemically characterised mRNA substrates [15–17,54], we performed primer extension analysis to identify the exact RNase G cleavage sites in the 5′ UTR of *hns* mRNA. Total RNA extracted from the WT, Δ*rng*, and Δ*rng*^comp^ strains cultivated under anaerobic conditions was used to synthesise cDNA, which formed three bands (a, b, and c) distinct from those in WT and Δ*rng*^comp^ cells (Fig 4B, left panel) and corresponded to sites in the 5′ UTR of *hns* mRNA (Fig 4B, right panel). When the full-length *hns* mRNA sequence (450 nucleotides) was analyzed using the M-Fold program (http://unafold.rna.albany.edu) [55], we observed the unique secondary structure of *hns* 5′ UTR, in which the identified RNase G cleavage sites, are in single-stranded regions, in six thermodynamically stable structures of 16 secondary structures. The analysis indicated two cleavage sites (b and c) in the putative Shine-Dalgarno sequence and another (a) in a region close to the transcriptional start site.

To demonstrate biochemically enzymatic cleavage of *hns* mRNA by RNase G, we carried out an *in vitro* cleavage assay using purified *S*. Typhimurium RNase G protein and synthetic RNA containing the full-length *hns* sequence. *In vitro* RNase G cleavage of the 5′-^32^P-end-labeled synthetic *hns* mRNA generated three major cleavage products (bands 1, 2, and 3 in Fig 4C) whose size corresponded to the cleavage sites detected by primer extension analysis (Fig 4D). We also observed RNase G-dependent cleavage products (1′-5′) that were not detected by primer extension analysis (Fig 4C and 4D). These products may be due to non-specific RNase G cleavage activity against the synthetic *hns* transcript *in vitro*, as noted for other substrates [16,17]. Nonetheless, these results provide experimental evidence that RNase G can cleave specific sites in the 5′ UTR of *hns* mRNA, possibly affecting *hns* mRNA stability.

## Effects of nucleotide substitutions in RNase G cleavage sites in 5′ UTR of *hns* mRNA on *S*. Typhimurium virulence

Although RNase G is known to preferentially cleave single-stranded, AU-rich sequences [11,13,14], the structural RNA determinants of RNase G cleavage activity have not yet been characterised. To examine whether RNase G cleavage is responsible for *hns* mRNA degradation, we introduced nucleotide substitutions at positions A-31, A-9, or U-8 in the *hns* 5′ UTR (A of the start codon defined as +1). This was then introduced into an *hns*::*cat* fusion construct to produce a reporter system expressing the 5′ UTR of *hns* mRNA fused to the chloramphenicol acetyl transferase (*cat*) coding region, with nucleotide substitutions from A to C at position -31 (A-31C), A to G at position -9 (A-9G), or U to C at position -8 (U-8C). These nucleotide substitutions were chosen to avoid AU-rich sequences which are known to be a property of RNase G cleavage sites. The fusion gene was constitutively expressed from a mutant tryptophan promoter (*trp*^c^) in a multi-copy plasmid in WT cells to measure CAT expression levels. Western blot analysis revealed that WT cells expressing the *hns*::*cat* fusion mRNAs with nucleotide substitutions at RNase G cleavage sites produced ~ 1.8- to 8.2-fold more CAT protein than those expressing WT *hns*::*cat* fusion mRNA (S4A Fig). The increased CAT protein levels produced by mutant *hns*::*cat* mRNAs was attributed to stabilisation of the fusion mRNA in the WT strain, as indicated by a strong correlation between CAT levels and the half-lives of the mutant fusion mRNAs (A-31C, A-9G, and U-8C: 7.9, 2.5, and 6.6 min, respectively, WT: 2.0 min; S4B Fig).

**Fig 5:**
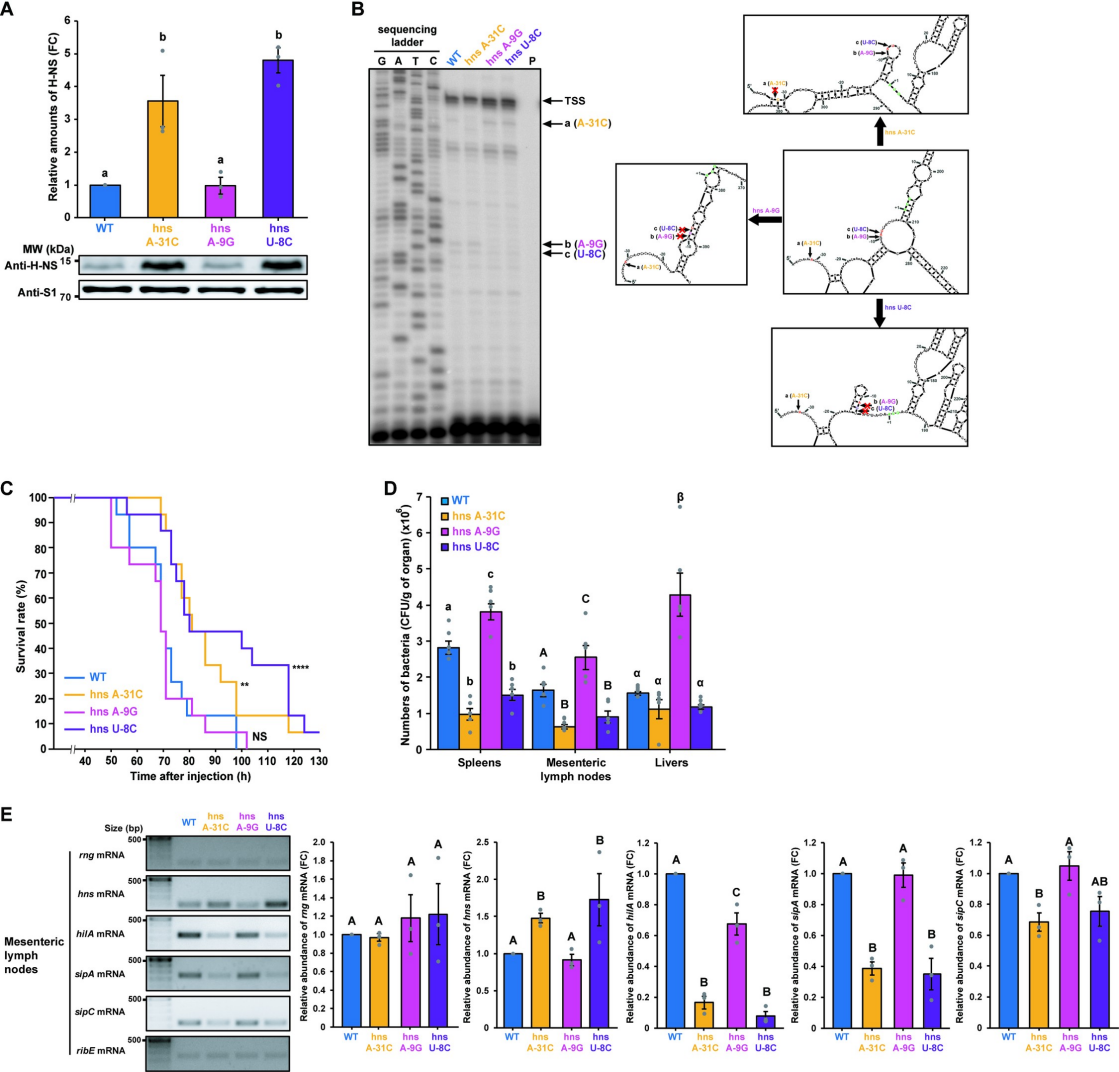
Effects of nucleotide substitutions in the RNase G cleavage sites of the *hns* mRNA 5′ UTR on *hns* expression. **(A)** Western blot analysis of H-NS in *S*. Typhimurium strains (WT, hns A-31C, hns A-9G, and hns U-8C). WT H-NS levels were set to 1. Ribosomal protein S1 was used as an internal standard. **(B)** Primer extension analysis of *hns* mRNA 5′ UTR containing the wild-type or mutated sequence (hns A-31C, hns A-9G, or hns U-8C) *in vivo*. Left: Total RNA samples isolated from the *S*. Typhimurium cultures used in (A) were analyzed. Sequencing ladders were synthesized with the same primer used in cDNA synthesis and PCR DNA encompassing the *hns* gene as a template. Right: The secondary structure of the full-length *hns* sequence containing the wild-type or mutated sequence (hns A-31C, hns A-9G, or hns U-8C) was deduced using the M-Fold program. Representative structures of the 5′ UTR that most frequently occur when the mutated sequences are used to deduce the secondary structure of full-length *hns* mRNA. Arrows indicate RNase G cleavage sites. Start codon is indicated by bold, green characters. TSS; transcription start site. **(C)** Survival rates and duration of BALB/c mice (*n* = 15 per group) infected with *S*. Typhimurium. strains (WT, hns A-31C, hns A-9G, and hns U-8C). Survival was monitored for 7 days. ** *P* < 0.01 and **** *P* < 0.0001 for hns A-31C-, hns A-9G-, or hns U-8C-infected mice vs. WT-infected mice (two-sided unpaired Student’s *t*-test). **(D)** Colonization assay for *S*. Typhimurium. BALB/c mice (*n* = 5 per group) intraperitoneally infected with *S*. Typhimurium strains (WT, hns A-31C, hns A-9G, and hns U-8C). Data represent the mean ± s. e. m. of at least three independent experiments. Statistically significant differences from one-way ANOVA with Student-Newman-Keuls test (*P* < 0.0001) are indicated by different letters: small letters indicate differences from spleens; large letters indicate differences from mesenteric lymph nodes; Greek symbols indicate differences from livers. **(E)** Characterisation of *hns* and SPI-1-related gene (*hilA*, *sipA*, and *sipC*) mRNA expression levels in the mesenteric lymph nodes of *S*. Typhimurium (WT, hns A-31C, hns A-9G, and hns U-8C)-infected mice (*n* = 3 per group). The relative mRNA abundance of *hns* and SPI-1-related genes is shown on the right side of the gel images. *hns* and SPI-1-related gene expression levels were normalized to *ribE* mRNA. Data represent the mean ± s. e. m. of at least three independent experiments. Statistically significant differences from one-way ANOVA with Student-Newman-Keuls tests are indicated by different letters. *P* < 0.05 for *hns* and *sipC* mRNA and *P* < 0.0001 for *hilA* and *sipA* mRNA.

To further investigate the effects of RNase G cleavage site alterations on *S*. Typhimurium virulence, we introduced the same nucleotide substitutions into the *hns* gene 5′ UTR in the *S*. Typhimurium genome using a CRISPR-Cas9 system [17,56] (S5 Fig) and measured H-NS levels in the mutant strains. Western blot analysis revealed that the mutant strains expressing *hns* mRNA containing A-31C (hns A-31C) or U-8C (hns U-8C) showed ~ 3.6- or 4.8-fold higher production of the H-NS protein, respectively, than that showed by WT cells expressing WT *hns* mRNA (Fig 5A). However, H-NS protein levels did not significantly change in the mutant strain expressing *hns* mRNA containing A-9G (hns A-9G; Fig 5A).

To analyze the *in vitro* RNase G reactivity of the *hns* mRNA carrying the 5′ UTR mutations, we carried out an *in vitro* cleavage assay using purified *S*. Typhimurium RNase G protein and synthetic full-length *hns* transcripts containing the WT or the mutated sequences (hns A-31C, hns A-9G, or hns U-8C) (S6A Fig). We observed that the amounts of cleavage products generated at site (1) were markedly diminished when the hns A-31C mutant transcript was used. However, the accumulation of cleavage products at site (2) was increased compared to that from the WT transcript (S6A Fig). The rate of accumulation of cleavage products at site (1) when the hns A-9G mutant transcript was used was similar to that when WT transcript was used, whereas the amounts of the cleavage products at sites (2) and (3) decreased compared to those from the WT transcript (S6A Fig). For the hns U-8C mutant transcript, cleavage product accumulation at sites (1) and (2) was similar to that for the WT transcript, whereas the accumulation of cleavage products at sites (3) was increased compared to that from the WT transcript (S6A Fig). Higher levels of uncleaved full-length transcripts were observed for the hns U-8C mutant transcript than those from other transcripts (S6B Fig). The results from *in vitro* cleavage of hns U-8C mutant transcript was not consistent with experimental results showing increased half-life of *hns* mRNA and H-NS expression levels when the U-8C mutation was introduced (Figs 5A and S4A Fig). We believe that this difference resulted from alterations of secondary structures of the *hns* 5′ UTR due to the incorporation of three G residues at the 5′-terminus of synthetic *hns* transcripts. These residues were incorporated because of the intrinsic property of T7 RNA polymerase used for the synthesis of synthetic *hns* transcripts (S6C Fig). When the secondary structures of synthetic *hns* transcripts were analyzed using the M-Fold program, the introduction of nucleotide substitution at the nucleotide A-31 (A-31C) resulted in the formation of a stem-loop that buried the cleavage site (1) in the region. We observed a similar result when the A-9G mutation is introduced (S6C Fig). Unlike these mutations, the U-8C mutation resulted in forming an open bulge in the region (S6C Fig). These results suggest that the introduction of nucleotide substitution in the *hns* transcript as well as addition of three G residues at the 5′-terminus resulted in alterations in the secondary structure of the *hns* 5′ UTR, leading to changes in RNase G reactivity on these synthetic transcripts.

We further investigated RNase G cleavage reactivity on *hns* mutant mRNA by performing primer extension analysis on total RNA from *S*. Typhimurium cells expressing WT or mutant *hns* mRNAs. The A-31C mutation resulted in diminished RNase G cleavage reactivity at site (a), whereas A-9G and U-8C mutations reduced RNase G cleavage reactivity at both sites (b and c) (Fig 5B, left panel). RNase G cleavage activity on the hns U-8C mRNA appeared to be different when analyzed *in vivo* using primer extension analysis compared to that analyzed *in vitro* (S6A Fig). When the secondary structures of *hns* mRNA 5′ UTR without three additional G residues were analyzed using the M-Fold program, all mutations lead to the formation of a stem-loops containing the corresponding cleavage site with high frequency (Fig 5B, right panel). We speculate that different secondary structures of the *hns* mRNA 5′ UTR can be formed because of three additional G residues at the 5′-terminus of synthetic transcripts, resulting in altered RNase G reactivity at cleavage sites in *hns* mRNA. Nonetheless, the effects of RNase G cleavage site mutations on the half-life of *hns* mRNA and expression of H-NS can be explained by altered RNase G reactivity on *hns* mRNA by the mutations *in vivo*.

When the pathogenicity of these mutant strains was tested in a mouse model, higher survival rates were observed in mice infected with hns A-31C or hns U-8C strains than the WT or hns A-9G (Fig 5C). In addition, the number of bacterial cells in several organs from the infected mice correlated with the virulence of the mutant strains in the mouse model (Fig 5D). Furthermore, *hns* expression was inversely correlated with the mRNA abundance of SPI-1-related genes in the spleens, livers, and mesenteric lymph nodes of mice infected with the mutant strains (Figs 5E and S7). Thus, these findings indicate that changes in *hns* mRNA stability due to alterations in RNase G cleavage activity in its 5′ UTR is a direct cause of changes in *S*. Typhimurium virulence.

### Regulation of RNase G levels in response to oxygen availability

**Fig 6:**
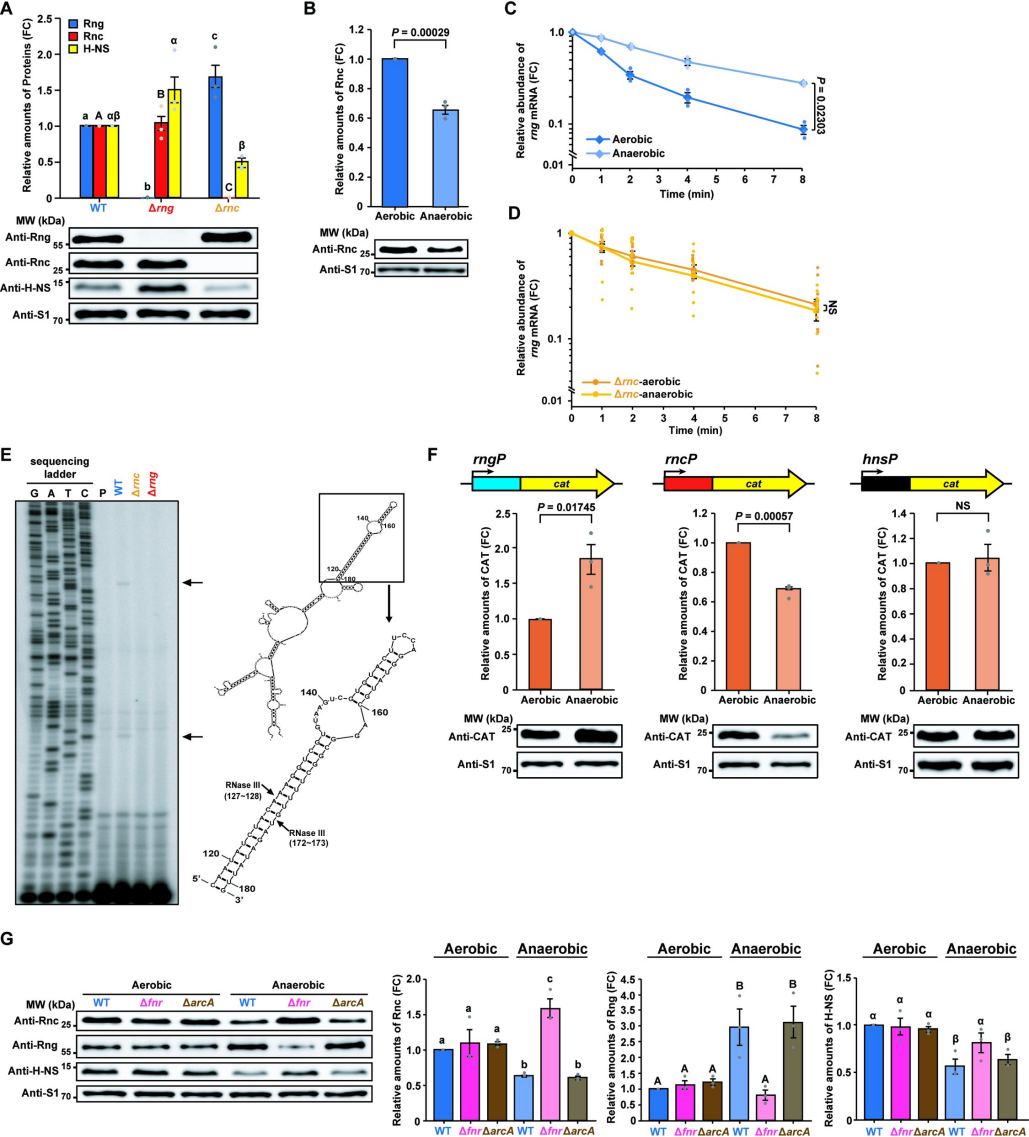
Increased RNase III activity down-regulates RNase G expression. **(A)** Western blot analysis of Rng, Rnc, and H-NS in *S*. Typhimurium strains (WT, Δ*rng*, and Δ*rnc*). WT Rng, Rnc, and H-NS levels were set to 1. **(B)** Western blot analysis of Rnc levels in WT *S*. Typhimurium under aerobic and anaerobic conditions. Rnc levels under aerobic conditions were set to 1. **(C)** Effects of oxygen on *rng* mRNA stability in the *S*. Typhimurium cultures used in (B) measured by qRT-PCR. **(D)** Effects of oxygen on *rng* mRNA stability in *S*. Typhimurium Δ*rnc* cells measured by qRT-PCR. NS; not significant. **(E)** Identification of RNase III cleavage sites in *rng* mRNA *in vivo*. Left: Primer extension analysis of total RNA from *S*. Typhimurium strains (WT, Δ*rnc*, and Δ*rng*). Black arrows indicate RNase III cleavage sites. Right: *rng* mRNA secondary structure was deduced using the M-Fold program. Black arrows indicate RNase III cleavage sites in *rng* mRNA identified by primer extension analysis. **(F)** Effects of oxygen on *rng*, *rnc*, and *hns* gene promoter activity in WT *S*. Typhimurium. Top: DNA fragments containing *rng*, *rnc*, or *hns* promoters cloned in-frame with the *cat* gene CDS into pCAT924. Bottom: Western blot analysis of CAT in WT *S*. Typhimurium harbouring *rngP*::*cat*, *rncP*::*cat* or *hnsP*::*cat* fusion constructs. WT CAT levels under aerobic conditions were set to 1. **(G)** Western blot analysis of Rnc, Rng, and H-NS to determine the effects of FNR and ArcA on *S*. Typhimurium strains (WT, Δ*fnr*, and Δ*arcA*) under aerobic and anaerobic conditions. WT Rnc, Rng, and H-NS levels under aerobic conditions were set to 1. For **(A)**, data represent the mean ± s. e. m. of at least three independent experiments. Statistically significant differences from one-way ANOVA with Student-Newman-Keuls test (*P* < 0.001 for Rng, *P* < 0.0001 for Rnc, and *P* < 0.05 for H-NS) are indicated by different letters: small letters indicate differences in Rng levels; capital letters indicate differences in Rnc levels; Greek symbols indicate differences in H-NS levels. For **(B)** and **(F)**, data represent the mean ± s. e. m. of three independent experiments. Statistically significant values from two-sided unpaired Student’s *t*-tests are indicated. For **(C)** and **(D)**, *rng* mRNA expression was normalized using 16S rRNA, and gene expression was quantified using the ΔΔCt method and represented on a semi-logarithmic plot. Data represent the mean ± s. e. m. of three or ten independent experiments. Statistically significant values from two-sided unpaired Student’s *t*-tests are indicated. For **(G)**, data represent the mean ± s. e. m. of at least three independent experiments. Statistically significant differences from one-way ANOVA with Student-Newman-Keuls test (*P* < 0.0001 for Rng, *P* < 0.001 for Rnc and H-NS) are indicated by different letters: small letters indicate differences in Rnc levels; large letters indicate differences in Rng levels; Greek symbols indicate differences in H-NS levels. For **(A)**, **(B)**, **(F)**, and **(G)**, ribosomal protein S1 was used as an internal standard.

Although we revealed that increased RNase G levels contributes toward the down-regulation of *hns* expression in the host environment, it remains unclear how *rng* expression is up-regulated under these conditions. Previously, we showed that *rng* mRNA abundance is down-regulated by RNase III in *E*. *coli* [19]; therefore, we examined whether RNase III regulates RNase G by measuring its levels in WT and *rnc*-deleted (Δ*rnc*; SL1344*rnc* + pACYC177) strains. RNase G protein levels were ~ 68% higher in the Δ*rnc* strain than in the WT, whereas H-NS levels were ~ 49% lower in the Δ*rnc* strain (Fig 6A).

When RNase III levels were measured in WT cells grown in the presence and absence of oxygen, it was found to be ~ 35% lower under anaerobic conditions than under aerobic conditions (Fig 6B). The increase in RNase G levels observed under anaerobic conditions (Fig 3B) was attributed to changes in the half-life of *rng* mRNA, which was around 2.5-fold higher in WT cells under anaerobic conditions (3.9 min) than under aerobic conditions (1.6 min; Fig 6C). We compared the *rng* transcript lifetime under anaerobic and aerobic conditions in cells lacking RNase III using qRT-PCR. We observed no significant changes in half-lives of *rng* mRNA when *rnc*-deleted cells were grown under aerobic or anaerobic conditions (Fig 6D). These results indicate that RNase III determines *rng* mRNA stability. Primer extension analysis of *rng* mRNA under aerobic conditions identified two cDNA bands generated in WT cells but not Δ*rnc* or Δ*rng* cells. These bands corresponded to sites in the double stranded regions of the secondary structure of *rng* mRNA (Fig 6E) and were very similar to those identified in *E*. *coli rng* mRNA [19]. Taken together, these results indicate that RNase III post-transcriptionally regulates RNase G expression depending on oxygen availability.

Since RNase G levels were only ~ 68% higher in the Δ*rnc* strain than in the WT strain (Fig 6A), the down-regulation of its expression by RNase III is unlikely to be wholly responsible for the three-fold increase in RNase G levels observed under anaerobic conditions (Fig 3B). Therefore, we tested whether oxygen availability affected *rng* promoter (*rngP*) activity using a reporter construct expressing *cat* under the control of the *S*. Typhimurium *rngP*. CAT levels were induced in WT cells grown under anaerobic conditions (Fig 6F, left panel), indicating that increased RNase G levels are also attributable to *rngP* induction under anaerobic conditions. Moreover, the promoter activity of the *rnc* gene (*rncP*) was found to be down-regulated under anaerobic conditions when measured using a *cat* construct fused to *rncP* (Fig 6F, middle panel), suggesting an additional molecular basis for the down-regulation of RNase III levels under anaerobic conditions (Fig 6B). However, *hns* promoter (*hnsP*) activity did not significantly change under anaerobic conditions when tested using a similar reporter construct expressing *cat* under the control of the *S*. Typhimurium *hnsP* (Fig 6F, right panel).

To understand the basis for the oxygen-dependent promoter activities of the *rnc*, *rng*, and *hns* genes, we analyzed their respective promoter sequence regions in *S*. Typhimurium. Bioinformatics analysis of these promoters (online database Prodoric) [57] indicated the existence of sequences similar to the binding sequences of the oxygen-sensitive transcription factors, fumarate nitrate reductase (FNR) and aerobic respiratory control (ArcA), in the *rnc* and *rng* genes (S8 Fig). Sequences similar to the binding sequences of FNR and ArcA were not identified in the *hns* gene (S8 Fig). This complex regulatory system has been well characterised in *S*. Typhimurium, wherein the DNA-binding proteins FNR and ArcA sense changes in oxygen availability and control the expression of many genes either alone or in cooperation with other regulators [58–61]. In strains that did not express FNR (Δ*fnr*) or ArcA (Δ*arcA*), RNase III levels increased by 2.5-fold in the Δ*fnr* strain but did not significantly change in the Δ*arcA* strain compared to the WT strain (Fig 6G). Conversely, the *rng* promoter was repressed, leading to a 3.7-fold reduction in RNase G expression in the Δ*fnr* strain compared to the WT strain, whereas RNase G levels did not significantly differ between the Δ*arcA* and WT strains (Fig 6G). The simultaneous up-regulation of RNase III and down-regulation of RNase G expression at the transcriptional level in anaerobically grown Δ*fnr* cells produced H-NS expression levels comparable to those in WT cells grown under anaerobic conditions (Fig 6G). Furthermore, *fnr* or *arcA* deletion did not significantly affect RNase III, RNase G, or H-NS levels under aerobic conditions (Fig 6G). Together, these findings indicate that FNR negatively and positively regulates RNase III and RNase G expression, respectively, at the transcriptional level under anaerobic conditions.

## Discussion

**Fig 7:**
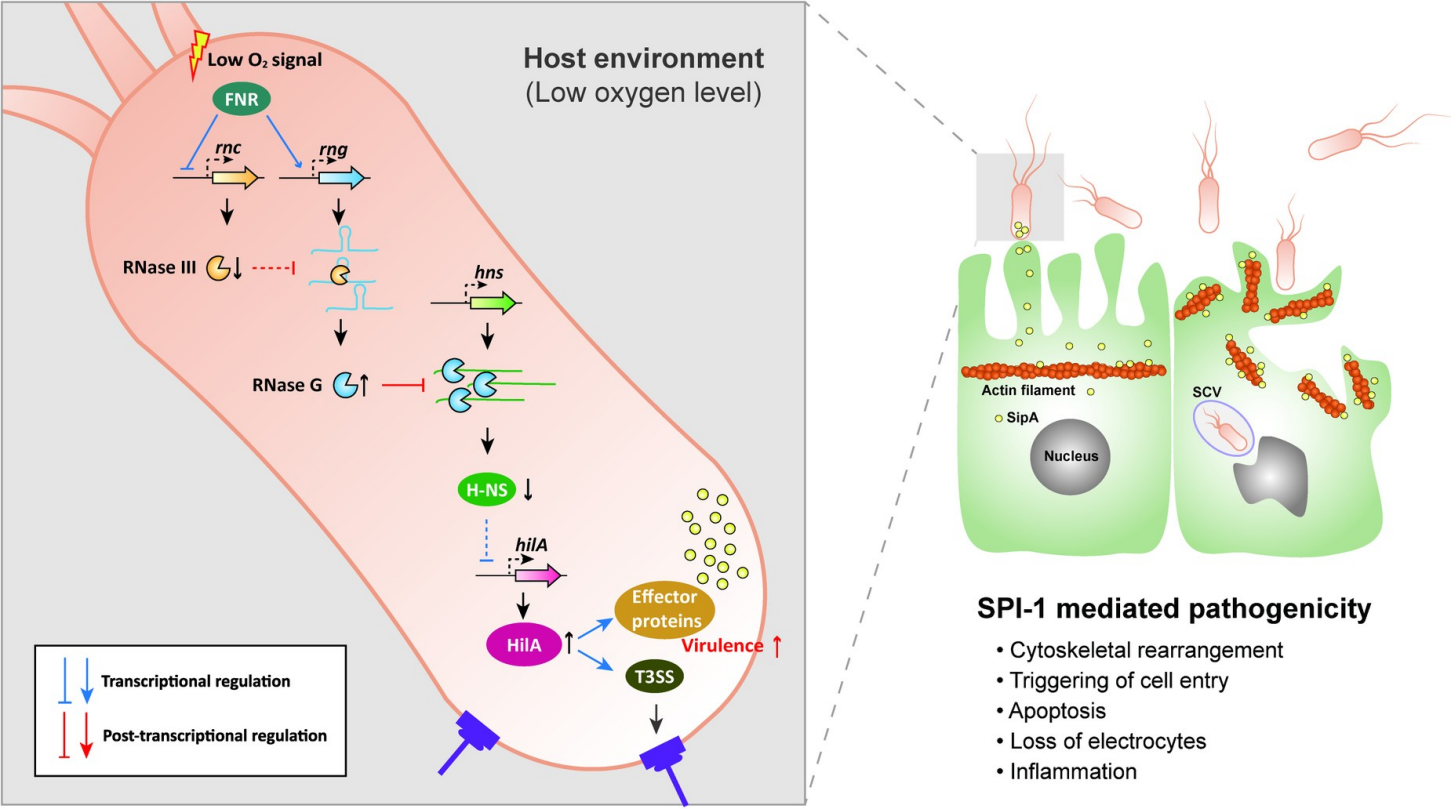
Model for the adaptive pathogenicity of *S*. Typhimurium to the host environment via the sequential modulation of RNase III and RNase G endoribonucleolytic activity. Host environmental signals triggered by the exposure of *S*. Typhimurium to low oxygen (anaerobic) conditions activate the regulation of the oxygen sensing regulator, FNR. FNR reduces RNase III levels by inhibiting *rnc* gene expression and promotes *rng* mRNA by activating *rng* gene expression, leading to increased cellular RNase G levels. RNase G induction accelerates the degradation of the *hns* mRNA 5′ UTR, thus downregulating *hns* mRNA levels. H-NS usually represses *hilA* gene expression; however, decreased cellular H-NS levels appear to activate the *hilA* promoter. HilA activates SPI-1 T3SS genes and induces the encoded effector proteins. These virulence cascades increase the invasiveness of *S*. Typhimurium to result in pathogenic *S*. Typhimurium infection.

The expression of *S*. Typhimurium virulence genes is known to be regulated by changes in environmental conditions [23,25]. The SPI-1 T3SS is considered essential for the invasion of the host intestinal epithelium by *Salmonella* and its transcription is regulated by a variety of global regulatory systems that converge on HilA expression via a complex feed-forward loop in which H-NS acts as a repressor [21]. This study highlights an endoribonuclease-mediated mechanism that regulates H-NS expression as an additional layer upstream of this loop. We observed that *hns* mRNA abundance decreased in *S*. Typhimurium in the host environment, with reduced oxygen availability repressing *rnc* gene promoter activity via FNR. This then decreased the activity of RNase III against *rng* mRNA, resulting in the rapid degradation of *hns* mRNA by RNase G. Consequently, decreased H-NS levels derepressed the genes involved in the SPI-1 T3SS (Fig 7). It is unlikely that changes in *S*. Typhimurium pathogenicity by RNase G levels, especially in the host environment, resulted from RNase G cleavage activity on other RNA substrates. Our experimental results showing that *hns* expression levels by genomic alterations in the RNase G cleavage sites in the 5′ UTR of *hns* are inversely correlated with *S*. Typhimurium virulence strongly support that this endoribonuclease-mediated regulation of *hns* mRNA abundance largely contributes to changes in *S*. Typhimurium virulence in the host environment.

The cellular levels of both RNase E and G are controlled post-transcriptionally via endoribonucleolytic cleavage of mRNAs encoding these enzymes in *E*. *coli* [19,62]. In addition, the activity of RNase E is regulated by protein inhibitors while such factors have not been identified for RNase G [63–65]. These findings indicate the importance of modulation of these endoribonucleases expression and activity in bacterial cellular physiology. In fact, for *S*. Typhimurium, mutations in RNase E and RNase III led to changes in pathogenicity that include attenuated virulence, impaired mobility, and reduced proliferation in the host environments [37]. However, these changes may well stem from pleiotropic effects driven by these mutant endoribonucleases, since RNase E and RNase III expression is known to affect the abundance of a large portion of mRNA species in *E*. *coli* [8–10,66].

A regulatory pathway involving RNase G and RNase III has been identified in *E*. *coli* in which incomplete 16S rRNA processing by RNase G is down-regulated by increased RNase III activity (not the protein level) under aminoglycoside antibiotics stress [19]. A recent study reported that enolase expression is regulated by the coordinated action of RNase III and RNase G in response to oxygen availability in *E*. *coli* [16]. However, it remains unclear how RNase III activity and expression are regulated in *E*. *coli* in response to different environmental changes [16,19,66]. We found that RNase III expression was negatively regulated by the oxygen sensing regulator, FNR, in *S*. Typhimurium cells grown anaerobically, whereas RNase G expression was enhanced. Together, our findings suggest how FNR can act as a global regulator of virulence in *S*. Typhimurium [61]. The key roles of FNR and its homologues in the virulence of some pathogens (i.e., *Neisseria meningitides* [67] and *Pseudomonas aeruginosa* [68]) have already been reported; however, future studies should investigate whether other pathogenic bacteria with FNR-associated virulence also adopt post-transcriptional regulatory pathways involving the modulation of endoribonuclease activity to rapidly respond to host environments.

## Materials and methods

### Ethics statement

All animal experiments were performed in accordance with the national guidelines for the use of animals in scientific research and were approved by Chung-Ang University Support Center (Approval No. CAU2012-0044).

### Animals

Mouse feeding and experimental procedures were performed as described previously [69,70]. Pathogen-free 6-week-old female BALB/c mice (survival assay: *n* = 10 or 15 per group, colonization assay: *n* = 5 per group, immune response assay: *n* = 5 per group, and RT-PCR analysis of organs: *n* = 3 per group) were purchased from Orient Bio.

### Bacterial strains and plasmid construction

*S*. Typhimurium and *E*. *coli* strains were grown at 37°C in LB medium supplemented with ampicillin (100 μg ml^-1^), tetracycline (5 μg ml^-1^), or chloramphenicol (5 μg ml^-1^), where appropriate, at 37°C under aerobic or anaerobic conditions. For anaerobic growth, *E*. *coli* cells were added to a 30 ml cylindrical bottle containing a sterilised stir bar and filled with LB medium containing ampicillin (100 μg ml^-1^), sealed with tape, and cultured on a magnetic stirrer [16].

The bacterial strains, plasmids, and primers used in this study are listed in S3 and S4 Tables. The Δ*rng* strain was constructed as described previously [19]. The Δ*rnc*, Δ*fnr*, and Δ*arcA* strains were constructed via one-step chromosomal gene inactivation using the method described by Datsenko and Wanner [71]. The Δ*rnc* strain was constructed by amplifying the tetracycline resistance marker in *E*. *coli* strain HT115. Replacement of the resistance marker with the *rnc* gene was confirmed by PCR amplification of the chromosomal region encompassing the *rnc* locus. The Δ*fnr* and Δ*arcA* strains were constructed by amplifying the chloramphenicol resistance marker in pKD3 [71]. Replacement of the resistance marker with the *fnr* or *arcA* gene was confirmed by PCR amplification of the chromosomal regions encompassing the *fnr* or *arcA* loci.

Genomic DNA from the *S*. Typhimurium SL1344 strain was used as a template for cloning. pSt-rng was constructed by amplifying the coding region of *S*. Typhimurium *rng*, which was digested with XhoI and BamHI and ligated into pACYC177. The pSt-rng-His plasmid was inserted with a hexahistidine affinity tag before the *rng* stop codon. The pSt-hns (WT)-cat, pSt-hns (A-31C)-cat, pSt-hns (A-9G)-cat, and pSt-hns (U-8C)-cat plasmids expressing St-*hns* (WT)::*cat*, St-*hns* (A-31C)::*cat*, St-*hns* (A-9G)::*cat*, and St-*hns* (U-8C)::*cat*, respectively, were constructed via multiple steps. To construct pSt-hns (WT)-cat, pSt-hns (A-31C)-cat, pSt-hns (A-9G)-cat, and pSt-hns (U-8C)-cat, NotI and NcoI sites were created using the overlap-extension PCR method. The PCR product was digested with NotI and NcoI and cloned into pCAT924 [66]. pSt-rngP-cat, pSt-rncP-cat, and pSt-hnsP-cat plasmids expressing *cat* under the control of the *S*. Typhimurium *rng*, *rnc*, or *hns* promoters, respectively, were constructed using the overlap-extension PCR method. PCR products were digested using NotI and NcoI and cloned into pCAT924.

The hns A-31C, hns A-9G, and hns U-8C strains were constructed using a CRISPR-Cas9 system. The pCas and pTargetF plasmids were both available via the non-profit plasmid distribution service Addgene (# 62225 and 62226, respectively) [72]. pTargetF (Amp) was constructed by replacing *aadA* (spectinomycin-resistant gene) with *bla* (ampicillin-resistant gene) using the overlap-extension PCR method. The resulting PCR product was digested using XhoI and MluI and cloned into pTargetF. To construct pTargetF (Amp)-original N_20_, a DNA fragment containing a 20 bp native protospacer (N_20_) of the *hns* gene 5′ UTR was amplified, digested using SpeI and EcoRI, and ligated into pTargetF (Amp). pTargetF (Amp)-artificial N_20_ was then inserted with N_20_ into pTargetF (Amp) and Basic Local Alignment Search Tool (http://www.rgenome.net/cas-offinder/) used to ensure that the selected 23 bp (N_20_-NGG) sgRNA target had no significant match elsewhere in the *S*. Typhimurium SL1344 genome.

### Genome editing using a CRISPR-Cas9 system

Nucleotide substitutions were introduced at positions A-31, A-9, and T-8 in the *hns* 5′ UTR sequence (A in start codon defined as +1) in the *S*. Typhimurium genome as described previously [17,72]. Two CRISPR events were performed using pTargetF (Amp)-original N_20_ and pTargetF (Amp)-artificial N_20_ with pCas, which was transformed into *S*. Typhimurium SL1344 and the transformed product spread onto LB agar containing kanamycin (50 μg ml^-1^). *S*. Typhimurium-competent cells harbouring pCas were prepared as described previously [73].

### Cell lines and culture conditions

Human intestinal epithelial cells (HCT116) were routinely cultured in McCoy’s 5A (Welgene) medium supplemented with 10% (v/v) heat-inactivated foetal bovine serum (Welgene) and 1% (v/v) penicillin-streptomycin solution (Welgene). Cell lines were cultivated at 37°C in a humidified atmosphere of 5% CO_2_ in air.

### Epithelial cell infection assay

*S*. Typhimurium strains were grown overnight in LB medium, washed with PBS, resuspended in PBS, and mixed with HCT116 at a multiplicity of infection (MOI) of 100. Infection assays were performed in the presence of antibiotics in serum free McCoy’s 5A medium. After incubation under 5% CO_2_ at 37°C for 1 h, extracellular bacteria were killed by treatment with gentamicin (100 μg ml^-1^) for 2 h (3 h post-infection) under the same conditions. Cells were then washed with PBS and intracellular bacteria were released using 0.25% sodium deoxycholate. The quantity of intracellular bacteria was assessed by measuring viable counts on non-selective and selective LB agar plates including appropriate antibiotics.

### Western blot analysis

Proteins were resolved and analyzed by western blotting, as described previously [9,70].

### Animal studies

Mice were intraperitoneally infected with 10^4^ CFUs of *S*. Typhimurium strains in 100 μl of PBS and euthanised after two days. The spleens, livers, and mesenteric lymph nodes were removed aseptically and viable intracellular bacterial cells counted as described previously [69].

### Flow cytometry analysis

To analyze CD11b+ cell frequency, mouse splenocytes were incubated with antibodies specific for murine CD11b (Tonbo Biosciences). To quantify inflammatory cytokines, serum IL-1α, IL-1β, IL-6, IL-10, IL-12p70, IL-17A, IL-23, IL-27, MCP-1, IFN-β, TNF-α, and GM-CSF levels were analyzed using a LEGENDplex mouse inflammation panel kit (BioLegend) according to the manufacturer’s instructions. Data were collected using an Attune NxT Acoustic Focusing Cytometer (Thermo Fisher Scientific) and analyzed using Flowjo software (BD Biosciences).

### Mass spectrometry analysis

Protein expression patterns in the supernatants and whole cell lysates of WT and Δ*rng* cells were compared following cultivation in LB medium with aeration (180 rpm) at 37°C. Cells harvested from 10 ml cultures were mixed with 300 μl of lysis buffer containing 8 M urea, 20 mM 3-[(3-cholamidopropyl) dimethylammonio]-1-propanesulfonate (CHAPS), and 10 mM dithiothreitol (DTT), and disrupted using 0.5 mm glass beads and a MINI BEADBEATER (BioSpec). Next, whole cell lysates were obtained by centrifugation at 4°C and 13,200 rpm for 15 min and protein concentration was determined using a Quick Start 1 × Dye Bradford reagent and bovine serum albumin standard (Bio-Rad Laboratories). Cellular enolase (Eno) levels in the whole cell lysates of WT and Δ*rng* cells were assessed by western blotting to evaluate whether *eno* mRNA levels were controlled by RNase G, as in *E*. *coli* [9,16].

Supernatants from the 10 ml cultures were treated with 10 mM DTT and 1× protease cocktail (Roche Diagnostics), filtered to remove cell debris using 0.2 μm membranes, and concentrated using centrifugal filter units with a cutoff value of 10 kDa (Merck Millipore). Concentrated supernatants were analyzed by SDS-PAGE and protein bands between 30 and 90 kDa were subjected to cysteine alkylation with 4.5% (w/v) iodoacetamide (Sigma-Aldrich) followed by trypsin digestion for mass spectrometry analysis, as described previously [74]. Eluted tryptic peptides were dissolved in 0.4% acetic acid and analyzed using a Velos Pro Mass analyser (Thermo Fisher Scientific) with a nano-liquid chromatography system and a Magic C18AQ column (75 μm × 75 mm), as described previously [75]. Tandem mass spectrometric data were analyzed using a SEQUEST search in Thermo Proteome Discoverer version 1.3 against a sequence database of proteins derived from the complete genome assembly of *S*. Typhimurium SL1344 (GCA_000210855.2) in the U.S. National Center for Biotechnology Information. The search options were as follows: average mass (*m/z*), maximum of one miscleavage site for trypsin digestion, precursor mass tolerance of 1.5 Da, fragment mass error of 1.0 Da, dynamic modification for methionine oxidation, static modification for cysteine alkylation, protein probability > 99%, and a false-discovery rate (FDR) < 0.01. Individual peptides were filtered at a probability > 0.95 to identify those with two or more unique peptides. Relative protein levels in each sample were quantitated using the normalized spectral abundance factor (NSAF) method [76].

### qRT-PCR

Total cellular RNA was extracted from cultures grown under aerobic and anaerobic conditions at 37°C to an OD_600_ of 0.6 and 0.3, respectively, using an RNeasy mini prep kit (Qiagen). qRT-PCR was performed as described previously [70].

### Northern blot analysis

Total RNA was isolated and analyzed by northern blotting, as described previously [9]. The random hexamer probes used to detect *hns* mRNA were synthesised using a random primer DNA labeling kit (Roche Diagnostics). PCR products containing the *hns* ORF were synthesised using RT-St-hns-F (+149) and St-hns (stop)-R primers and used as a template to synthesise random hexamer probes. The oligonucleotide probe M1-R was used to probe M1.

### Primer extension analysis

Primer extension analysis was performed as described previously [16,70]. To analyze the RNase G cleavage sites in the *hns* 5′ UTR, total cellular RNA was extracted from cells (WT, hns A-31C, hns A-9G, or hns U-8C) grown under anaerobic conditions at 37°C to an OD_600_ of 0.6 using St-hns-R (+30) primers. To analyze *in vitro*-cleaved synthetic *hns* RNA, the cleavage products were purified by phenol extraction and ethanol precipitation and hybridised with the 5′-^32^P-labeled primer, St-hns-R (+30). To identify the exact RNase III cleavage site in *rng* mRNA, total cellular RNA was extracted from cells cultured under aerobic conditions at 37°C to an OD_600_ of 0.6 using phenol extraction and ethanol precipitation, and then hybridised with the 5′-^32^P-labeled primer, St-rng (+219)-R.

### Purification of *S*. Typhimurium RNase G

*S*. Typhimurium RNase G (Rng) was purified from *E*. *coli* strain N3433*rng* containing pSt-rng-His using Ni-NTA agarose (Qiagen). Cultures were grown to an OD_600_ of 0.7 and harvested. RNase G was eluted from the columns using 250 mM imidazole, concentrated, and stored as described previously [9].

### RNA synthesis and *in vitro* cleavage assay

Synthetic *hns* RNA containing the 5′ UTR (WT, hns A-31C, hns A-9G, or hns U-8C) was synthesised from PCR DNA using a MEGAscript T7 kit (Thermo Fisher Scientific) according to the manufacturer’s instructions. PCR DNA was amplified from *S*. Typhimurium SL1344 genomic DNA (WT, hns A-31C, hns A-9G, or hns U-8C) using two primers, St-T7-hns-F and St-hns (stop)-R. RNA transcripts were labeled at the 5′ end using [γ-^32^P]ATP (Perkin Elmer, Waltham, MA, USA) and T4 polynucleotide kinase (New England Biolabs, Ipswich, MA, USA) and purified using 12% polyacrylamide gel containing 8 M urea. RNase G cleavage assays were performed as described previously [9].

### *cat* and *rng* mRNA stability assays

WT cells harbouring pSt-hns (WT)-cat, pSt-hns (A-31C)-cat, pSt-hns (A-9G)-cat, and pSt-hns (U-8C)-cat plasmids were cultured overnight in LB medium at 37°C with aeration, diluted 1:100 in same medium, and incubated at 37°C to an OD_600_ of 0.6. To stop RNA transcription, rifampicin (Sigma-Aldrich) was added to the cultures at a final concentration of 1 mg ml^-1^. For the zero-time point, culture samples were taken before rifampicin addition. To measure the half-life of *cat* mRNA, culture samples were taken 1, 2, 4, and 8 min after rifampicin addition and qRT-PCR analysis was performed as described above. To measure the half-life of *rng* mRNA, overnight cultures of WT or Δ*rnc* cells grown in LB medium at 37°C with aeration were diluted 1:100 in same medium and incubated at 37°C to an OD_600_ of 0.6 and 0.3, respectively, in the presence or absence of oxygen. Total RNA preparation and qRT-PCR were carried out as described above.

### RNA isolation from the organs of *S*. Typhimurium-infected mice

Three 6-week-old female mice were intraperitoneally infected with 10^4^ CFUs of the WT, hns A-31C, hns A-9G, or hns U-8C strains in 100 μl of PBS. Mice were euthanised after 2 days and their spleens, livers, and mesenteric lymph nodes were removed aseptically and homogenised in 500 μl of ice-cold TRIzol (Thermo Fisher Scientific). Total RNA samples were purified by phenol extraction and ethanol precipitation.

### Quantification and statistical analysis

The numerical data used in all main figures are included in S1 Data. The statistical details of all experiments are included in the figure legends. Multiple comparisons were performed using SAS v. 9.2 with the Student-Newman-Keuls test (SAS Institute) and Student’s *t*-tests were used for control comparisons in SAS v. 9.2 and SigmaPlot 10.0 (Systat Software). Data represent the mean ± standard error of the mean (s. e. m) and *P* < 0.05 was considered to indicate statistical significance. See S5 Table for exact statistics and reproducibility.

## Supporting information

S1 Data Excel spreadsheet containing, in separate sheets, the underlying numerical data and statistical analysis for Figure panels: 1A, 1B, 1C, 1D, 1E, 1F, 2B, 3A, 3B, 3C, 4A, 5A, 5C, 5D, 5E, 6A, 6B, 6C, 6D, 6F, and 6G.
(XLSX)

S1 Fig Dose-dependent survival of mice after *S.* Typhimurium infection. Mice were injected with 10^2^, 10^3^, 10^4^, and 10^5^ CFUs per mice (CFUs contained in 100 μl) of WT cells and incubated at 22 ± 1°C, and mortality was monitored daily. Kaplan-Meier survival curves were determined from three independent experiments. The control corresponds to the injection of PBS alone. Data are representative of three independent experiments, and similar results were obtained. ** *P* < 0.01 and **** *P* < 0.0001 for 10^2^-, 10^3^-, 10^4^-, or 10^5^ CFUs-injected mice versus PBS-treated mice (two-sided unpaired Student’s *t*-test). NS; not significant.
(TIF)

S2 Fig Inflammatory cytokine levels in infected mice. Serum was collected from mice that were left uninfected or infected with *S*. Typhimurium strains (WT, Δ*rng*, or Δ*rng*^comp^) for 60 h. The serum was analyzed for the expression of cytokines IL-1β, IL-12p70, IL-17A, IL-23, IL-27, IFN-β, and GM-CSF by multiplex cytokine analysis. The data are presented as the mean ± s. e. m. of at least two independent experiments, and statistically significant differences are indicated with different letters (one-way ANOVA with Student-Newman-Keuls test, not significant). n. d.; not detectable.
(TIF)

S3 Fig Protein expression patterns of enolase and type III secretion system effector proteins in WT and Δ_*rng*_ cells of *S.* Typhimurium strain SL1344. **(A)** Growth and protein production during growth of the cells in LB with aeration at 37°C. The incubation periods for the samples of exponential (3 h), early-stationary (9 h), stationary (24 h), and death phase (48 h) are denoted on the graph. The data are presented as the mean ± s. e. m. of three independent experiments. **(B)** Protein expression profiles in concentrated supernatants and whole-cell lysates of *S*. Typhimurium strains (WT and Δ*rng*), showing abundance changes in the secretion of type III secretion system (T3SS) effector proteins and flagellar proteins during different growth phases. Separated protein bands in four lanes (3 h, 9 h, 24 h, and 48 h) of the WT and Δ*rng* strains were sliced into three gel pieces (red dot line boxes) between 30 and 90 kDa for identification of proteins in the pooled data sets generated by tandem mass spectrometry, as shown in S1 and S2 Tables, and the normalized spectral abundance factor (NSAF) for comparing relative quantity was calculated from peptide spectrum match (PSM) counts as shown in Table 1 in the main text. Seven specific protein bands (SipA, SipB, FlgK, FliC, FliD, SipC, and FlgL) identified in SDS-PAGE analyzes of the secreted protein samples by in-gel digestion and tandem mass spectrometry are denoted to the right of the gel here and in Fig 2 in the main text. The relative band intensity after normalization to FliC/FliD levels in each lane (representative results of three independent culture experiments) is presented as a mean and standard deviation error bar in the histogram of Fig 2B in the main text. **(C)** Western blot intensities of Enolase (Eno) in *S*. Typhimurium strains (WT and Δ*rng*) at the indicated culture periods. WT Eno levels were set to 1. For **(B)** and **(C)**, M; size marker.
(TIF)

S4 Fig RNase G cleavage sites-dependent expression of CAT and stability of *cat* mRNA on the *hns::cat* fusion. **(A)** RNase G cleavage sites-dependent expression of CAT on the *hns*::*cat* fusion. Top: for each construct, a DNA fragment containing *trp*^c^ promoter, the 5′ UTR from TSS and the *hns* CDS for the first 10 amino acids was cloned in-frame with the CDS of the chloramphenicol acetyl transferase (*cat*) gene in the pCAT924 vector. RNase G cleavage sites are indicated in blue bold characters and nucleotide substitutions are indicated in red bold characters. Red arrows indicate primers used for qRT-PCR in (B). Bottom: WT strain harboring *hns*::*cat* fusion constructs were grown in LB at 37°C to an OD_600_ of 0.6, and were collected for western blot analysis of CAT using protein-specific polyclonal antibodies. WT CAT levels were set to 1. The data are presented as the mean ± s. e. m. of at least three independent experiments, and statistically significant differences are indicated with different letters (one-way ANOVA with Student-Newman-Keuls test, *P* < 0.01). **(B)** RNase G cleavage sites-dependent stability of *cat* mRNA on the *hns*::*cat* fusion. Total RNA samples of *S*. Typhimurium cultures used in (A), were prepared from the cultures 0, 1, 2, 4, and 8 min after the addition of rifampicin (1 mg ml^-1^) and cDNA synthesis was performed using random hexamer, and analyzed of *cat* mRNA levels using qRT-PCR. The expression levels of *cat* mRNA were normalized using 16S rRNA mRNA levels. Gene expression levels were quantified using the ΔΔCt method and represented semi-logarithmic plot. The data are presented as the mean ± s. e. m. of three independent experiments. Statistically significant values from two-sided unpaired Student’s *t*-tests are indicated. For **(A)**, ribosomal protein S1 was used as an internal standard to evaluate the amounts of cell extract in each lane.
(TIF)

S5 Fig Sanger sequencing confirmation of point mutation. Sequencing shows successful subsequent introduction of a point mutation in the *hns* 5′ UTR. TSS; Transcription start site.
(TIF)

S6 Fig *In vitro* cleavage of the synthetic full-length *hns* transcripts containing the wild-type or mutated sequences (hns A-31C, hns A-9G, or hns U-8C). **(A)** *In vitro* RNase G cleavage of the 5′-^32^P-end-labeled synthetic full-length *hns* transcripts containing the wild-type or the mutated sequences (hns A-31C, hns A-9G, or hns U-8C) generated cleavage products. Bold, blue characters indicate hydrolysis products. Black arrows indicate the cleavage sites identified in Fig 4B (1, 2, and 3). Grey arrows indicate (1′-5′) nonspecific RNase G cleavage sites. **(B)** The relative amount of RNase G cleavage product from the wild-type or the mutated *hns* transcripts was assessed by measuring the radioactivity of each cleavage product and plotted. **(C)** The secondary structures of the full-length *hns* transcripts containing the wild-type or mutated sequences (hns A-31C, hns A-9G, or hns U-8C). The secondary structures were deduced using the M-Fold program. Arrows indicate RNase G cleavage sites. The start codon is indicated by bold, green characters.
(TIF)

S7 Fig Characterization of *hns* and SPI-1-related genes mRNAs expression in *S.* Typhimurium cells during infection. Total RNA was isolated from the spleens and livers of *S*. Typhimurium strains (WT, hns A-31C, hns A-9G, and hns U-8C)-infected mice (*n* = 3 mice per group), and cDNA was synthesized from the total RNA purified from each organ. The relative abundance of each group of *hns* and SPI-1-related genes mRNAs was quantified and is shown right side the gel images. The expression levels of *hns* and SPI-1-related genes mRNAs were normalized using *ribE* mRNA levels. The data are presented as the mean ± s. e. m. of at least three independent experiments, and statistically significant differences are indicated with different letters (one-way ANOVA with Student-Newman-Keuls test, *P* < 0.05 for *hns* and *sipC* mRNA in spleens and *hns*, *hilA*, and *sipC* mRNA in livers, *P* < 0.01 for *sipA* mRNA in spleens, and *P* < 0.0001 for *hilA* mRNA in spleens and *sipA* mRNA in livers, respectively; small letters indicate a difference from spleens; Greek symbols indicate a difference from livers).
(TIF)

S8 Fig Analysis of binding of FNR and ArcA to *rnc*, *rng*, and *hns* promoters. **(A)** Predicted FNR and ArcA binding sites in the promoter region of *rnc* using the online software Prodoric Virtual Footprint Promoter Analysis Version 3.0 online tool to predict *E*. *coli* K12 FNR and ArcA binding sites. **(B)** Predicted FNR and ArcA binding sites in the promoter region of *rng* using the online software Prodoric Virtual Footprint Promoter Analysis Version 3.0 online tool to predict *E*. *coli* K12 FNR and ArcA binding sites. **(C)** Predicted FNR and ArcA binding sites in the promoter region of *hns* using the online software Prodoric Virtual Footprint Promoter Analysis Version 3.0 online tool to predict *E*. *coli* K12 FNR and ArcA binding sites. For **(A)**, **(B)**, and **(C)**, FNR and ArcA binding sites are highlighted in black box, and a score indicating how closely the binding site match FNR and ArcA binding sites consensus logo are given. A perfect match to the consensus sequence scores 8.93 and 7.44, respectively, as determined by Prodoric.
(TIF)

S1 Table Mass spectrometric data obtained from the in-gel digestion of gel bands containing the majority of T3SS effector proteins and flagellar proteins in supernatants of wild-type cells of *Salmonella* Typhimurium strain SL1344 by filtering protein identities with 2 or more unique peptides. (PDF)

S2 Table Mass spectrometric data obtained from the in-gel digestion of gel bands containing the majority of T3SS effector proteins and flagellar proteins in supernatants of *rng*-deleted cells of *Salmonella* Typhimurium strain SL1344 by filtering protein identities with 2 or more unique peptides. (PDF)

S3 Table Bacterial strains and plasmids used in this study. ^1^See METHOD DETAILS for detailed description scheme for plasmids. Abbreviation: Amp^r^, ampicillin resistance; Km^r^, kanamycin resistance; Tc^r^, tetracycline resistance; Cm^r^, chloramphenicol resistance; Spt^r^, spectinomycin resistance; Str^r^, streptomycin resistance.
(PDF)

S4 Table Primers used in this study. (PDF)

S5 Table Statistics and reproducibility. All statistical tests, biological replicates, exact *P* values, and significance for all graphs in this manuscript.
(PDF)
