# Supplementary figures and images for: Endoribonuclease-mediated control of hns mRNA stability constitutes a key regulatory pathway for Salmonella Typhimurium pathogenicity island 1 expression
Source: PLoS Pathog. 2021 Feb 1;17(2):e1009263. doi: 10.1371/journal.ppat.1009263 (PMC7877770; doi:10.1371/journal.ppat.1009263)

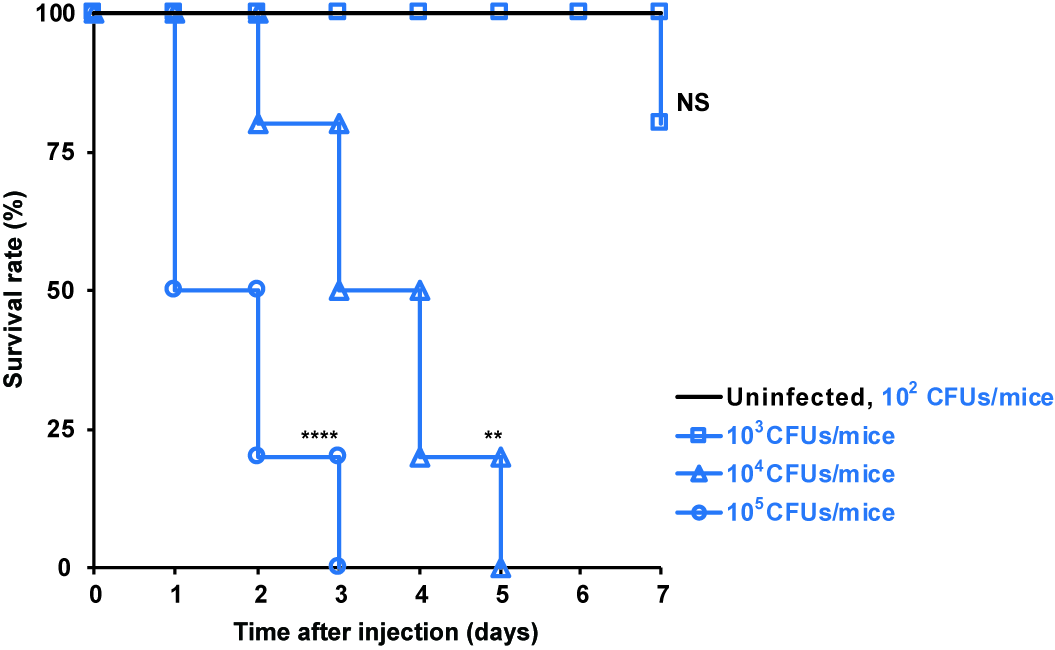

Supplement: S1 Fig — Mice were injected with 102, 103, 104, and 105 CFUs per mice (CFUs contained in 100 μl) of WT cells and incubated at 22 ± 1°C, and mortality was monitored daily. Kaplan-Meier survival curves were determined from three independent experiments. The control corresponds to the injection of PBS alone. Data are representative of three independent experiments, and similar results were obtained. ** P < 0.01 and **** P < 0.0001 for 102-, 103-, 104-, or 105 CFUs-injected mice versus PBS-treated mice (two-sided unpaired Student’s t-test). NS; not significant. (TIF) [file ppat.1009263.s002.tif]

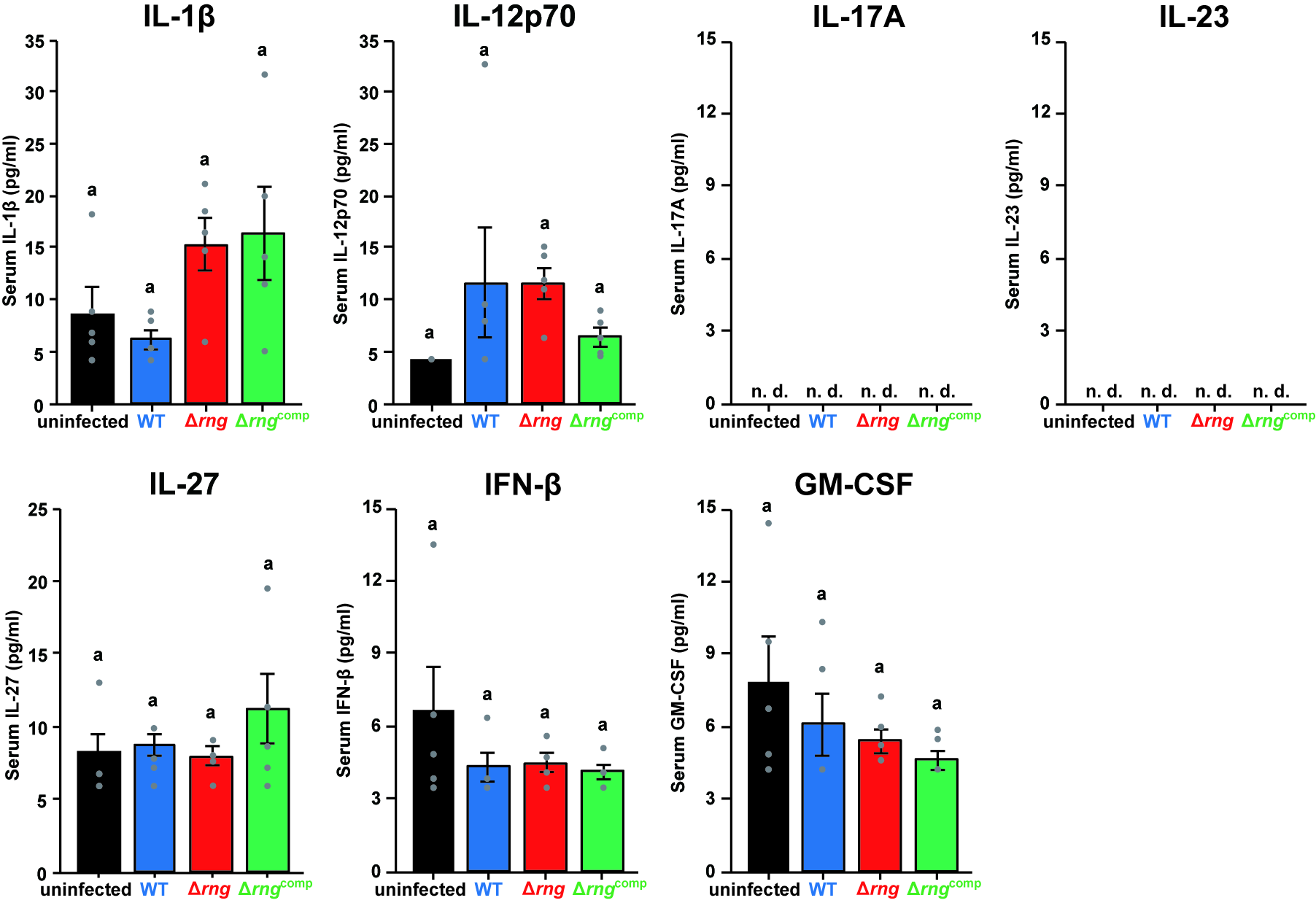

Supplement: S2 Fig — Serum was collected from mice that were left uninfected or infected with S. Typhimurium strains (WT, Δrng, or Δrngcomp) for 60 h. The serum was analyzed for the expression of cytokines IL-1β, IL-12p70, IL-17A, IL-23, IL-27, IFN-β, and GM-CSF by multiplex cytokine analysis. The data are presented as the mean ± s. e. m. of at least two independent experiments, and statistically significant differences are indicated with different letters (one-way ANOVA with Student-Newman-Keuls test, not significant). n. d.; not detectable. (TIF) [file ppat.1009263.s003.tif]

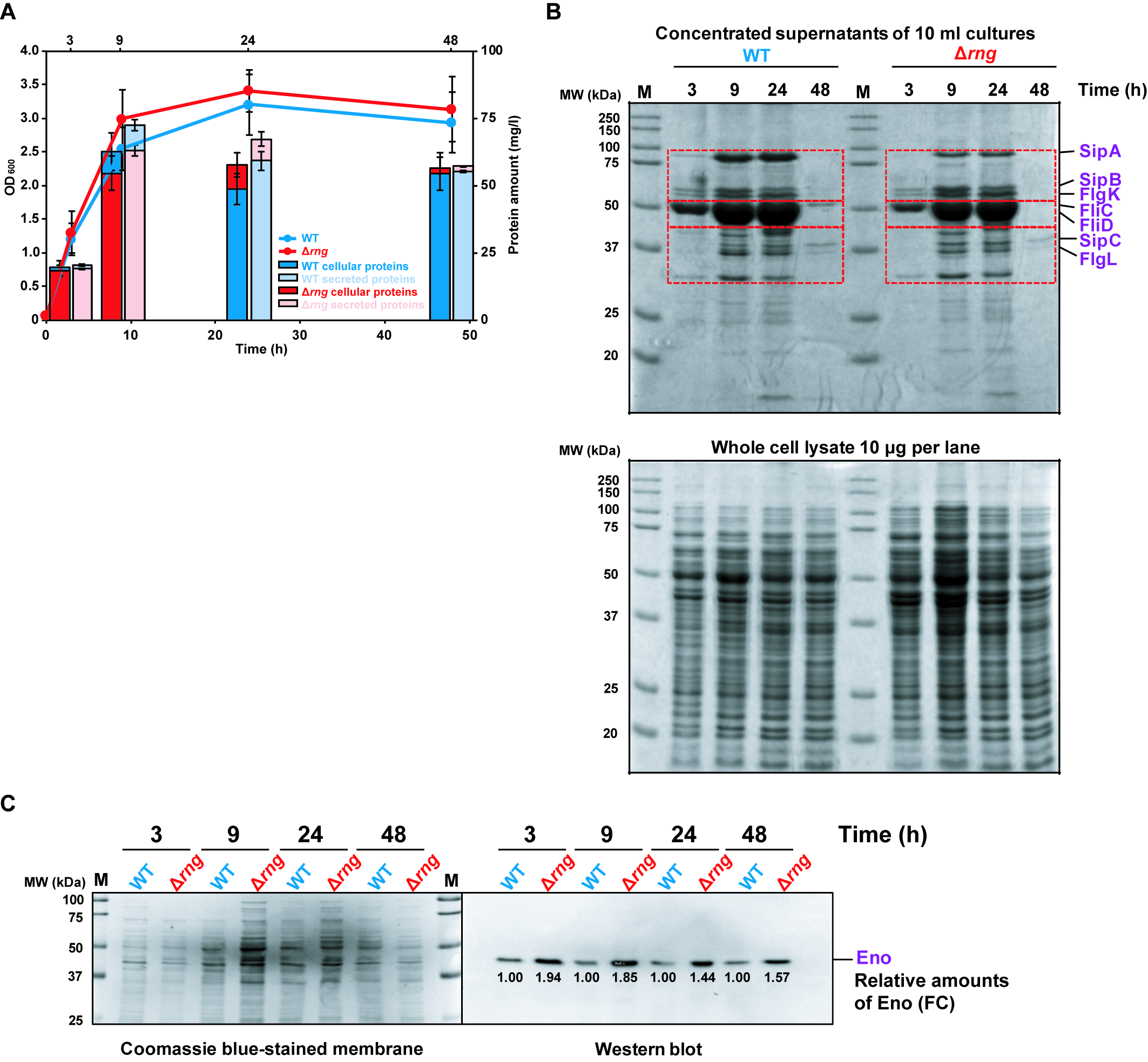

Supplement: S3 Fig — (A) Growth and protein production during growth of the cells in LB with aeration at 37°C. The incubation periods for the samples of exponential (3 h), early-stationary (9 h), stationary (24 h), and death phase (48 h) are denoted on the graph. The data are presented as the mean ± s. e. m. of three independent experiments. (B) Protein expression profiles in concentrated supernatants and whole-cell lysates of S. Typhimurium strains (WT and Δrng), showing abundance changes in the secretion of type III secretion system (T3SS) effector proteins and flagellar proteins during different growth phases. Separated protein bands in four lanes (3 h, 9 h, 24 h, and 48 h) of the WT and Δrng strains were sliced into three gel pieces (red dot line boxes) between 30 and 90 kDa for identification of proteins in the pooled data sets generated by tandem mass spectrometry, as shown in S1 and S2 Tables, and the normalized spectral abundance factor (NSAF) for comparing relative quantity was calculated from peptide spectrum match (PSM) counts as shown in Table 1 in the main text. Seven specific protein bands (SipA, SipB, FlgK, FliC, FliD, SipC, and FlgL) identified in SDS-PAGE analyzes of the secreted protein samples by in-gel digestion and tandem mass spectrometry are denoted to the right of the gel here and in Fig 2 in the main text. The relative band intensity after normalization to FliC/FliD levels in each lane (representative results of three independent culture experiments) is presented as a mean and standard deviation error bar in the histogram of Fig 2B in the main text. (C) Western blot intensities of Enolase (Eno) in S. Typhimurium strains (WT and Δrng) at the indicated culture periods. WT Eno levels were set to 1. For (B) and (C), M; size marker. (TIF) [file ppat.1009263.s004.tif]

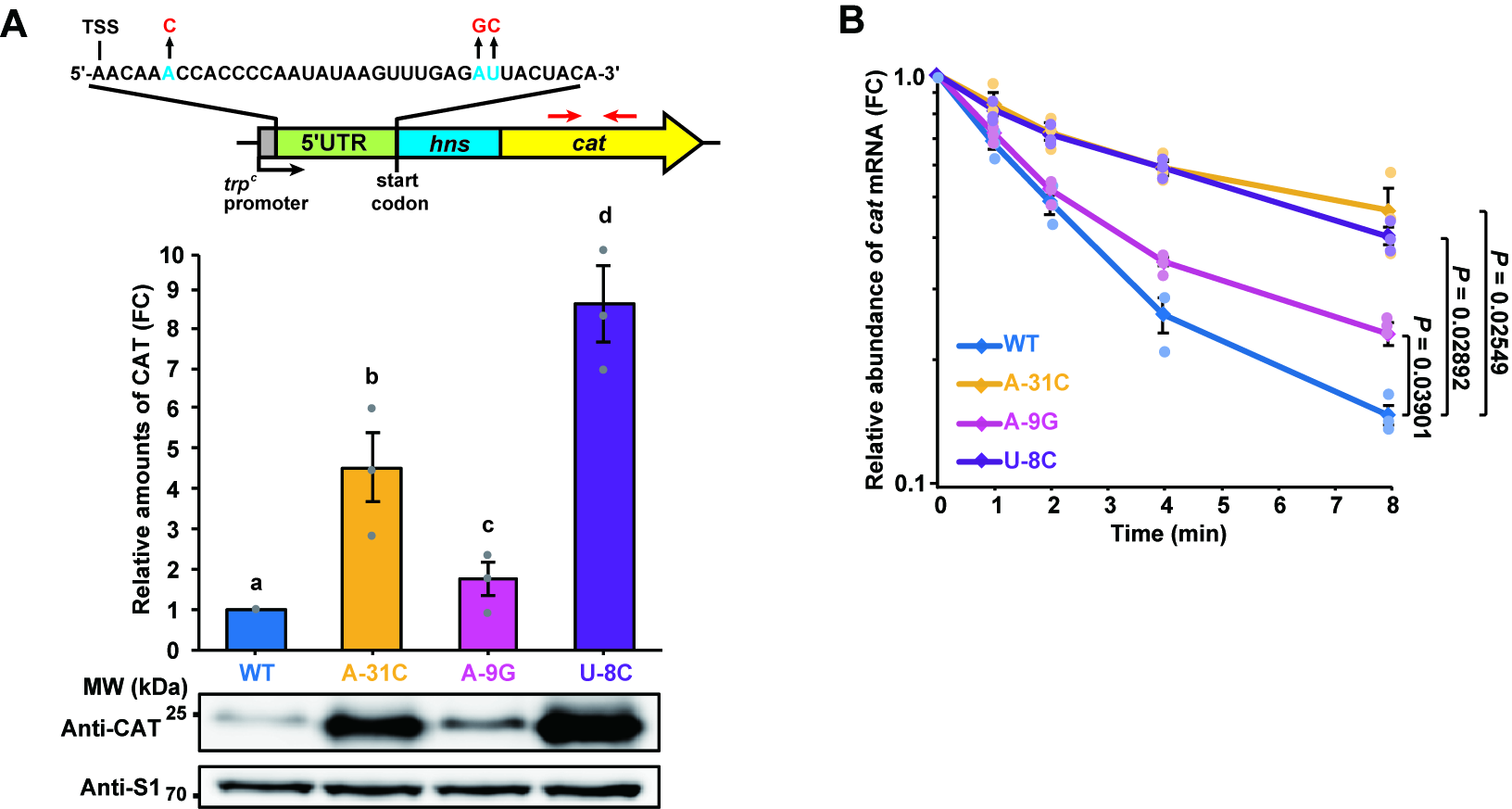

Supplement: S4 Fig — (A) RNase G cleavage sites-dependent expression of CAT on the hns::cat fusion. Top: for each construct, a DNA fragment containing trpc promoter, the 5′ UTR from TSS and the hns CDS for the first 10 amino acids was cloned in-frame with the CDS of the chloramphenicol acetyl transferase (cat) gene in the pCAT924 vector. RNase G cleavage sites are indicated in blue bold characters and nucleotide substitutions are indicated in red bold characters. Red arrows indicate primers used for qRT-PCR in (B). Bottom: WT strain harboring hns::cat fusion constructs were grown in LB at 37°C to an OD600 of 0.6, and were collected for western blot analysis of CAT using protein-specific polyclonal antibodies. WT CAT levels were set to 1. The data are presented as the mean ± s. e. m. of at least three independent experiments, and statistically significant differences are indicated with different letters (one-way ANOVA with Student-Newman-Keuls test, P < 0.01). (B) RNase G cleavage sites-dependent stability of cat mRNA on the hns::cat fusion. Total RNA samples of S. Typhimurium cultures used in (A), were prepared from the cultures 0, 1, 2, 4, and 8 min after the addition of rifampicin (1 mg ml-1) and cDNA synthesis was performed using random hexamer, and analyzed of cat mRNA levels using qRT-PCR. The expression levels of cat mRNA were normalized using 16S rRNA mRNA levels. Gene expression levels were quantified using the ΔΔCt method and represented semi-logarithmic plot. The data are presented as the mean ± s. e. m. of three independent experiments. Statistically significant values from two-sided unpaired Student’s t-tests are indicated. For (A), ribosomal protein S1 was used as an internal standard to evaluate the amounts of cell extract in each lane. (TIF) [file ppat.1009263.s005.tif]

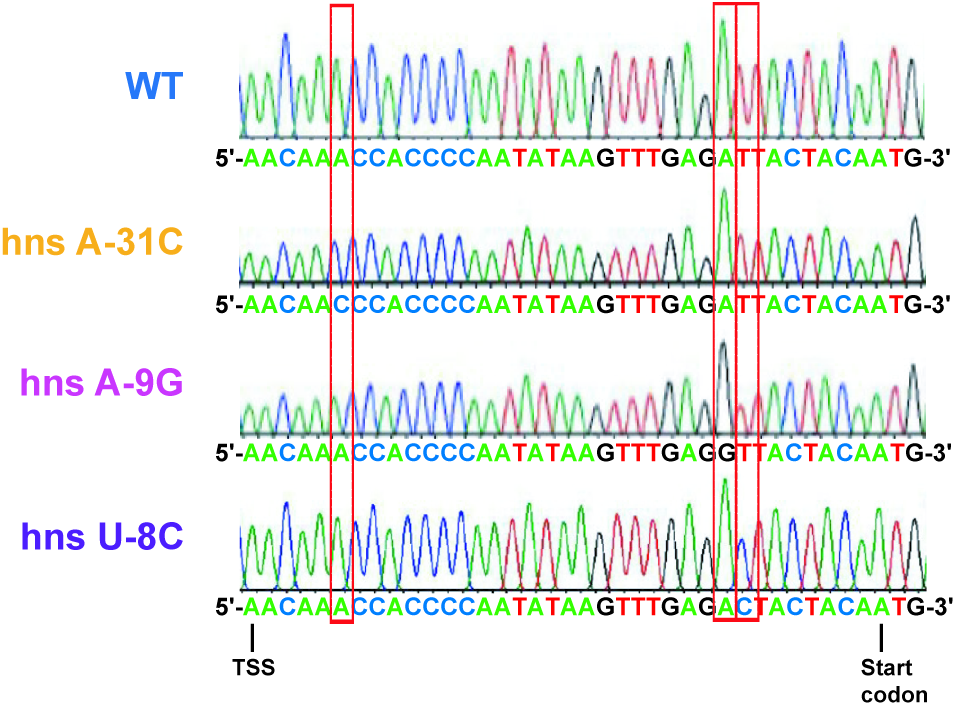

Supplement: S5 Fig — Sequencing shows successful subsequent introduction of a point mutation in the hns 5′ UTR. TSS; Transcription start site. (TIF) [file ppat.1009263.s006.tif]

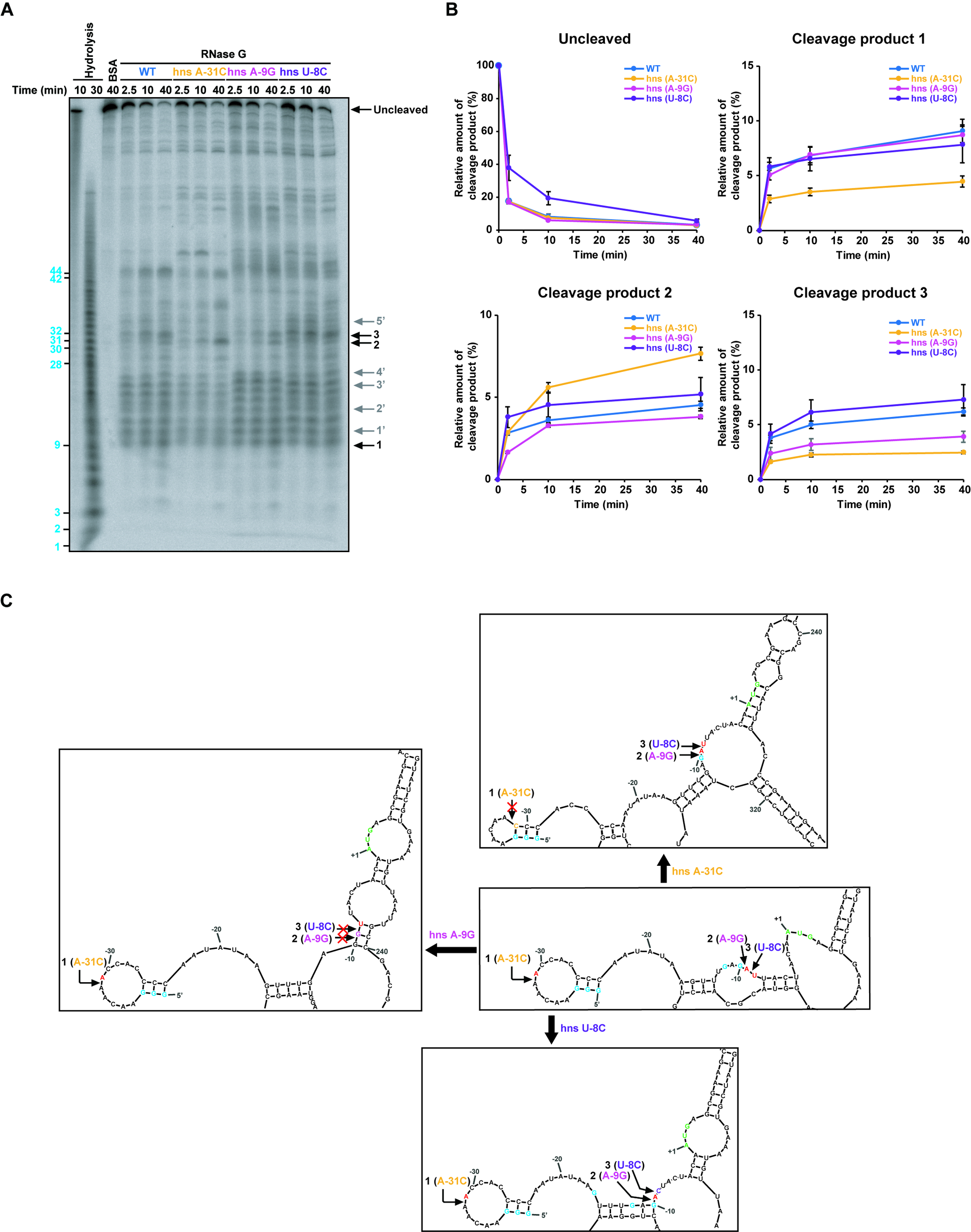

Supplement: S6 Fig — (A) In vitro RNase G cleavage of the 5′-32P-end-labeled synthetic full-length hns transcripts containing the wild-type or the mutated sequences (hns A-31C, hns A-9G, or hns U-8C) generated cleavage products. Bold, blue characters indicate hydrolysis products. Black arrows indicate the cleavage sites identified in Fig 4B (1, 2, and 3). Grey arrows indicate (1′-5′) nonspecific RNase G cleavage sites. (B) The relative amount of RNase G cleavage product from the wild-type or the mutated hns transcripts was assessed by measuring the radioactivity of each cleavage product and plotted. (C) The secondary structures of the full-length hns transcripts containing the wild-type or mutated sequences (hns A-31C, hns A-9G, or hns U-8C). The secondary structures were deduced using the M-Fold program. Arrows indicate RNase G cleavage sites. The start codon is indicated by bold, green characters. (TIF) [file ppat.1009263.s007.tif]

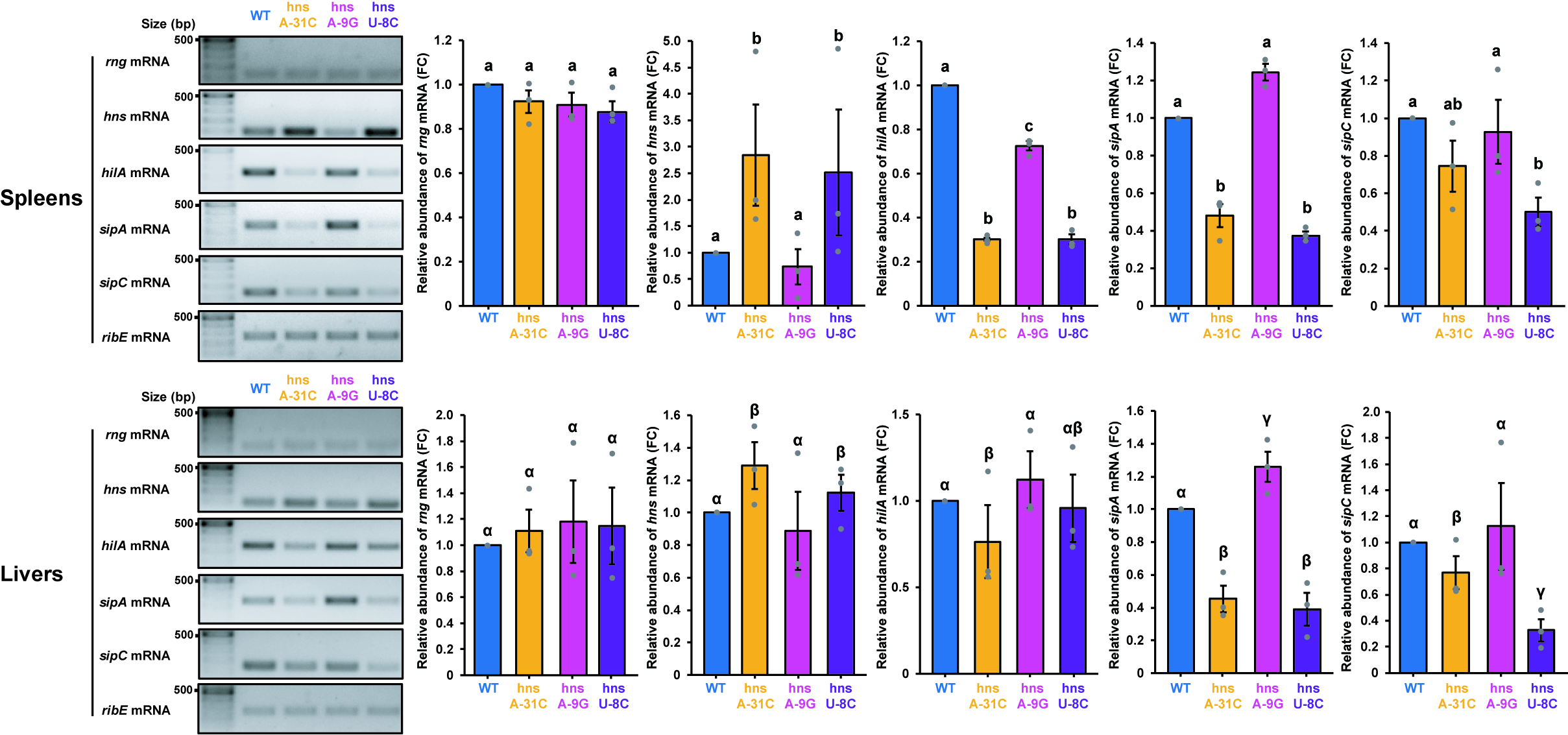

Supplement: S7 Fig — Total RNA was isolated from the spleens and livers of S. Typhimurium strains (WT, hns A-31C, hns A-9G, and hns U-8C)-infected mice (n = 3 mice per group), and cDNA was synthesized from the total RNA purified from each organ. The relative abundance of each group of hns and SPI-1-related genes mRNAs was quantified and is shown right side the gel images. The expression levels of hns and SPI-1-related genes mRNAs were normalized using ribE mRNA levels. The data are presented as the mean ± s. e. m. of at least three independent experiments, and statistically significant differences are indicated with different letters (one-way ANOVA with Student-Newman-Keuls test, P < 0.05 for hns and sipC mRNA in spleens and hns, hilA, and sipC mRNA in livers, P < 0.01 for sipA mRNA in spleens, and P < 0.0001 for hilA mRNA in spleens and sipA mRNA in livers, respectively; small letters indicate a difference from spleens; Greek symbols indicate a difference from livers). (TIF) [file ppat.1009263.s008.tif]

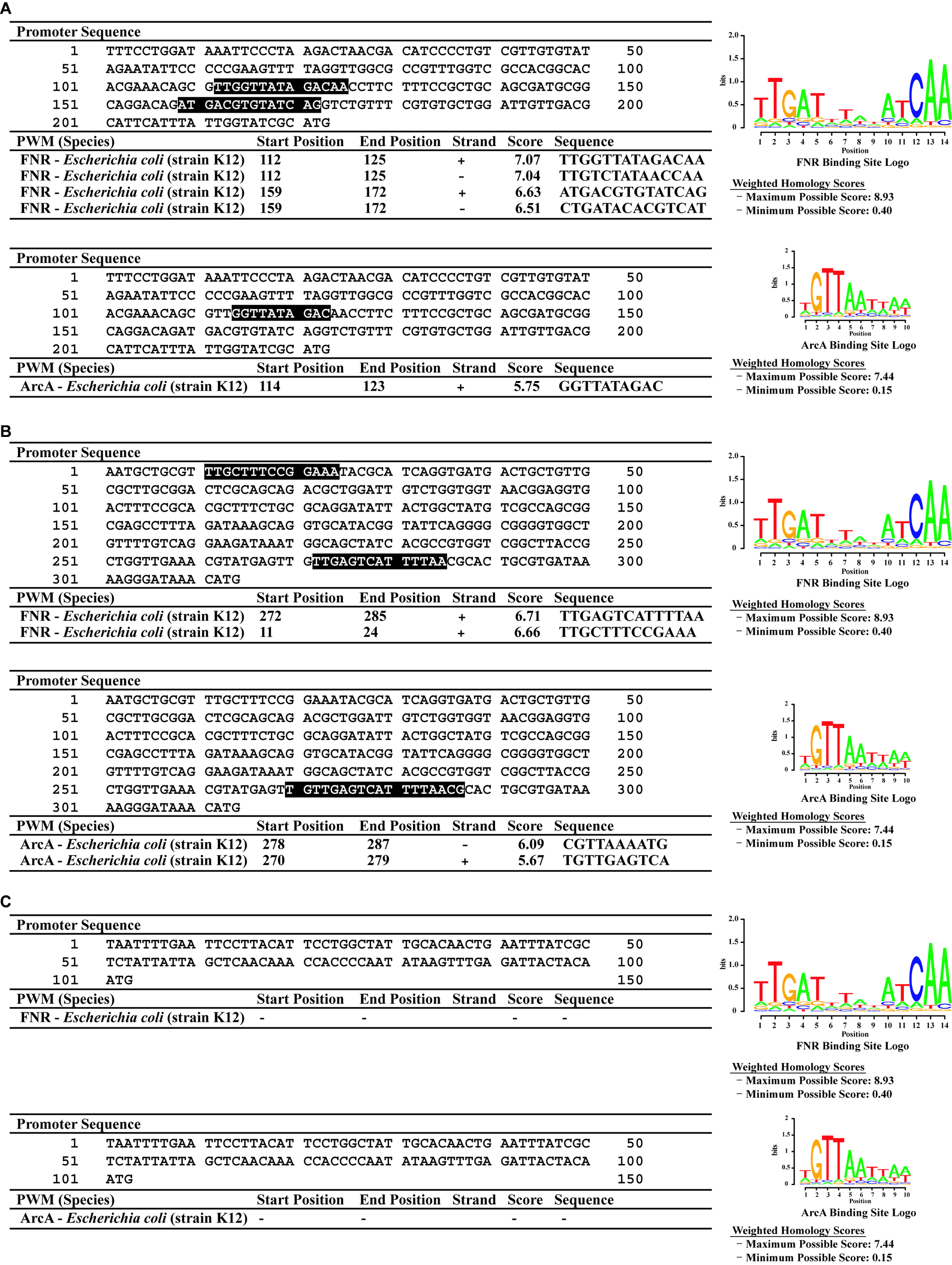

Supplement: S8 Fig — (A) Predicted FNR and ArcA binding sites in the promoter region of rnc using the online software Prodoric Virtual Footprint Promoter Analysis Version 3.0 online tool to predict E. coli K12 FNR and ArcA binding sites. (B) Predicted FNR and ArcA binding sites in the promoter region of rng using the online software Prodoric Virtual Footprint Promoter Analysis Version 3.0 online tool to predict E. coli K12 FNR and ArcA binding sites. (C) Predicted FNR and ArcA binding sites in the promoter region of hns using the online software Prodoric Virtual Footprint Promoter Analysis Version 3.0 online tool to predict E. coli K12 FNR and ArcA binding sites. For (A), (B), and (C), FNR and ArcA binding sites are highlighted in black box, and a score indicating how closely the binding site match FNR and ArcA binding sites consensus logo are given. A perfect match to the consensus sequence scores 8.93 and 7.44, respectively, as determined by Prodoric. (TIF) [file ppat.1009263.s009.tif]
