# Supplementary material for: Endoribonuclease-mediated control of hns mRNA stability constitutes a key regulatory pathway for Salmonella Typhimurium pathogenicity island 1 expression
Source: PLoS Pathog. 2021 Feb 1;17(2):e1009263. doi: 10.1371/journal.ppat.1009263 (PMC7877770; doi:10.1371/journal.ppat.1009263)
Supplement: S1 Table — (PDF) [file ppat.1009263.s010.pdf]

S1 Table. Mass spectrometric data obtained from the in-gel digestion of gel bands containing the majority of T3SS effector proteins and flagellar proteins in supernatants of wild-type cells of *Salmonella* Typhimurium strain SL1344 by filtering protein identities with 2 or more unique peptides.

Accession	Description	Score	Coverage	# Unique Peptides	#Peptides	#PSMs	#AAs	MW [kDa]			
CBW17983	Flagellin FliC	2539	50.91	16	25	605	495	51.6			
	Sequence	#PSMs	Modifications	XCorr	SpScore	Charge	MH+ [Da]	ΔM [ppm]	m/z [Da]	RT [min]	# Missed Cleavage
	INSAKDDAAGQAIANR	66		6.26	2210.7	2	1615.5	-133.1	808.3	45.5	1
	LNEIDRVSGQTQFNGVK	87		6.26	2143.3	3	1906.7	310.4	636.2	90.8	1
	AQPDLAEAAATTTENPLQK	9		6.21	3063.7	2	1970.3	96.1	985.7	61.7	0
	SRIEDSDYATEVSNMSR	61		5.94	2027.8	3	1961.8	345.4	654.6	56.3	1
	IEDSDYATEVSNmSR	8	M13(Oxidation)	5.92	2698.1	2	1734.0	138.7	867.5	52.1	0
	QINSQTLGLDTLNVQQK	50		5.87	1695.6	2	1901.4	134.0	951.2	63.9	0
	IEDSDYATEVSNMSR	9		5.84	2574.4	2	1717.2	-375.1	859.1	56.7	0
	SRIEDSDYATEVSNmSR	20	M15(Oxidation)	5.81	2348.6	3	1977.2	66.9	659.7	51.5	1
	LGGADGKTEVVSIGGK	55		5.43	1338.4	3	1488.9	114.5	497.0	101.7	1
	IDAALAQVDTLR	64		5.13	1779.9	2	1286.4	-49.2	643.7	62.1	0
	DDAAGQAIANRFTANIK	1		5.00	1421.0	2	1777.2	152.8	889.1	61.0	1
	AALTAAGVTGTASVVK	6		4.84	2736.0	2	1417.4	-158.7	709.2	53.9	0
	VGDDYYSATQNK	3		4.57	1759.2	2	1361.0	-276.3	681.0	45.8	0
	ASATGLGGTDQKIDGDLK	2		4.51	1209.9	2	1747.4	-271.8	874.2	52.1	1
	DDAAGQAIANR	31		4.37	1581.0	2	1102.2	19.7	551.6	40.3	0
	TYAASKAEGHNFK	2		4.35	1576.1	2	1424.1	-325.1	712.6	36.2	1
	VSGQTQFNGVK	39		4.31	1108.0	2	1165.2	-42.0	583.1	44.3	0
	IDGDLKFDDTTGK	1		3.97	1910.5	2	1425.0	-368.4	713.0	51.9	1
	SQSALGTAIER	36		3.89	1482.4	2	1134.0	695.5	567.5	103.3	0
	NVQVANADLTEAK	1		3.81	1243.9	2	1374.4	653.6	687.7	50.6	0
	ASATGLGGTDQK	3		3.56	813.3	2	1106.9	677.0	554.0	36.2	0
	FDDTTGKYYAK	1		3.54	1607.3	2	1309.0	-351.3	655.0	46.0	1
	SDLGAVQNR	29		3.38	1300.8	2	960.0	-17.1	480.5	100.2	0
	DGSISINTTK	9		3.36	577.1	2	1036.9	726.5	518.9	44.0	0
	DGYYEVSVDK	1		3.02	889.4	2	1175.1	-137.9	588.0	51.9	0
	MSYTDNNGK	4		2.86	206.6	1	1031.3	1172.2	1031. 3	35.3	0
	mSYTDNNGK	4	M1(Oxidation)	2.77	470.4	2	1046.9	753.4	523.9	25.9	0

	TEVVSIGGK	3		2.72	662.0	2	890.3	280.8	445.6	42.9	0
CBW18959	SPI-1 Type III secretion system effector protein SipA	2193	61.75	47	47	602	685	73.9			
	DAEALKEVFTNSNNVAGK	7		5.82	2266.9	2	1908.1	15.0	954.6	60.5	1
	GTTGETTSFDEVDGVTSK	61		5.67	2303.0	2	1832.2	146.1	916.6	95.9	0
	AGPAYESYLNKPGVDR	27		5.45	1850.7	2	1737.4	-318.4	869.2	54.4	0
	TQATNLAANLSAVR	68		5.18	2721.3	2	1430.4	-119.1	715.7	59.9	0
	LAEIALQFAR	58		5.10	2012.4	2	1133.0	595.4	567.0	81.9	0
	HLEMQTLIPLLLR	38		4.77	2044.5	2	1577.9	-59.6	789.4	82.9	0
	NVFAQIPADKLPDPK	7		4.62	795.2	2	1653.3	-348.9	827.2	61.2	1
	VGAEYTAQIIKDGLK	9		4.60	1693.6	2	1606.1	-472.4	803.6	60.9	1
	TDTLQSDTTVITGNK	7		4.60	2145.8	2	1594.2	-312.2	797.6	53.1	0
	SNLTHLVDKAAAK	9		4.48	1793.3	2	1368.2	-296.8	684.6	48.9	1
	EKLAEIALQFAR	2		4.34	1131.3	2	1389.4	-176.1	695.2	66.8	1
	mETLKEVITHHPQK	3	M1(Oxidation)	4.32	1730.7	3	1707.8	-95.9	569.9	48.6	1
	LENLEKQLLDIIK	2		4.28	1949.5	3	1570.3	244.3	524.1	77.6	1
	SIIGKPVQATVHGVDDNK	3		4.28	1115.8	3	1879.2	62.8	627.1	51.3	0
	VGAEYTAQIIK	9		4.26	1488.7	2	1194.5	963.3	597.8	54.4	0
	HLEmQTLIPLLLR	9	M4(Oxidation)	4.26	1396.7	2	1594.6	411.9	797.8	72.2	0
	EVFTNSNNVAGKK	5		4.24	847.5	2	1408.8	153.2	704.9	42.4	1
	GPQLEDFPALIK	9		4.23	1573.6	2	1329.2	510.0	665.1	69.7	0
	KAIMEFAGLFR	7		4.21	1620.0	2	1282.9	-550.5	641.9	68.4	1
	SALNATSDSPEAK	19		4.19	1028.8	2	1291.1	-175.7	646.1	42.8	0
	AVEALDmcHQK	8	M7(Oxidation);<br>C8(Carbamidomethyl)	4.16	865.6	2	1320.0	1112.8	660.5	39.8	0
	AQcSDIDKHPELK	7	C3(Carbamidomethyl)	4.09	1248.2	2	1543.0	826.7	772.0	42.1	1
	AIMEFAGLFR	23		4.09	1580.1	2	1156.2	703.3	578.6	74.8	0
	METLKEVITHHPQK	4		4.09	1255.2	3	1692.3	159.4	564.8	50.5	1
	KALEPDTSTPFPVR	2		4.08	851.3	2	1558.3	-304.2	779.7	54.8	1
	EKSAFGPWLPETK	2		4.02	903.5	2	1490.3	-275.3	745.6	60.4	1
	VITTVDGLHMQR	10		3.99	1170.9	2	1370.4	-190.8	685.7	54.0	0
	IPEPAAGPVPDGGK	13		3.97	1084.2	2	1306.2	581.0	653.6	49.4	0
	HIDNSNHDNSR	16		3.95	1162.9	2	1309.2	-45.5	655.1	6.3	0
	LTQEQGTSVGR	20		3.84	1206.1	2	1176.5	215.4	588.8	101.3	0
	AVEALDMcHQK	7	C8(Carbamidomethyl)	3.82	1022.0	2	1302.0	-411.9	651.5	45.3	0
	ESATATLSGEIK	47		3.81	1654.7	2	1208.6	1021.1	604.8	95.9	0
	QLLDIIKNNTGGELSK	4		3.74	687.4	2	1744.3	156.4	872.6	64.6	1
	EVITHHPQKEK	9		3.73	406.2	2	1347.0	331.1	674.0	33.1	1
	SAFGPWLPETKK	1		3.72	748.6	2	1361.3	-236.5	681.1	59.7	1
	AFDGLRAEILPNDTIK	5		3.71	856.7	2	1773.5	-305.6	887.2	63.5	1

	VITTVDGLHmQR	2	M10(Oxidation)	3.67	1565.7	2	1386.1	-359.7	693.6	48.7	0
	KAImEFAGLFR	3	M4(Oxidation)	3.62	726.7	2	1299.2	-282.2	650.1	62.2	1
	IPEPAAGPVPDGGKK	3		3.62	1649.6	2	1433.3	-264.8	717.1	47.5	1
	EVFTNSNNVAGK	4		3.48	1192.3	2	1280.1	-201.8	640.6	44.7	0
	AImEFAGLFR	3	M3(Oxidation)	3.46	1011.0	2	1171.1	-299.6	586.0	67.3	0
	HIDNSNHDNSRK	8		3.46	533.0	2	1437.4	-19.5	719.2	6.3	1
	SNLTHLVDK	14		3.41	895.7	2	1027.0	-192.3	514.0	45.6	0
	TQPPVIMPGmQTEIK	1	M10(Oxidation)	3.30	316.6	2	1688.3	720.5	844.6	58.3	0
	AEAKLENLEK	2		3.30	765.1	2	1145.2	-76.2	573.1	44.4	1
	HINNSRSHVDNSQR	1		3.29	534.4	3	1665.7	606.5	555.9	31.5	1
	AEILPNDTIK	1		3.19	608.9	1	1115.4	1040.6	1115.4	52.9	0
	AGTTDNDNSQTDK	1		3.17	704.6	2	1367.4	26.8	684.2	5.5	0
	SAFGPWLPETK	18		2.97	547.8	1	1234.5	891.1	1234.5	64.0	0
	NVFAQIPADK	1		2.96	753.0	2	1102.7	-529.5	551.8	54.8	0
	TFIDNSQR	1		2.89	666.3	2	981.1	23.3	491.0	42.2	0
	EVITHHPQK	1		2.71	286.8	2	1089.0	-239.2	545.0	29.8	0
	ALEPDTSTPFPVR	1		2.67	557.5	2	1430.2	-311.0	715.6	59.2	0
CBW18855	Flagellin FljB	1621	17.39	10	10	388	506	52.5			
	INSAKDDAAGQAIANR	66		6.26	2210.7	2	1615.5	-133.1	808.3	45.5	1
	LNEIDRVSGQTQFNGVK	87		6.26	2143.3	3	1906.7	310.4	636.2	90.8	1
	SRIEDSDYATEVSNMSR	61		5.94	2027.8	3	1961.8	345.4	654.6	56.3	1
	IEDSDYATEVSNmSR	8	M13(Oxidation)	5.92	2698.1	2	1734.0	138.7	867.5	52.1	0
	IEDSDYATEVSNMSR	9		5.84	2574.4	2	1717.2	-375.1	859.1	56.7	0
	SRIEDSDYATEVSNmSR	20	M15(Oxidation)	5.81	2348.6	3	1977.2	66.9	659.7	51.5	1
	DDAAGQAIANRFTANIK	1		5.00	1421.0	2	1777.2	152.8	889.1	61.0	1
	IDAALAQVDALR	1		4.97	1884.4	2	1256.9	346.2	628.9	59.5	0
	DDAAGQAIANR	31		4.37	1581.0	2	1102.2	19.7	551.6	40.3	0
	VSGQTQFNGVK	39		4.31	1108.0	2	1165.2	-42.0	583.1	44.3	0
	SQSALGTAIER	36		3.89	1482.4	2	1134.0	695.5	567.5	103.3	0
	SDLGAVQNR	29		3.38	1300.8	2	960.0	-17.1	480.5	100.2	0
CBW17984	Flagellar hook associated protein FliD	783	59.96	28	28	208	467	49.8			
	INVTQLAAAQSLATK	15		6.82	2881.5	2	1530.5	480.9	765.8	60.9	0
	LNNTSSYLTQQFTAMNK	6		5.84	2500.7	3	1962.7	242.5	654.9	62.0	0
	TMAEIGITQDGTSGK	11		5.82	1843.8	2	1510.1	304.1	755.6	51.9	0
	LNNTSSYLTQQFTAmNK	5	M15(Oxidation)	5.71	2356.9	3	1978.2	-3.7	660.1	57.1	0
	TTFATTKEQLGDTSVTSR	3		5.63	2104.0	2	1943.3	-401.8	972.2	50.7	1
	TmAEIGITQDGTSGK	8	M2(Oxidation)	5.48	2142.9	2	1527.1	931.3	764.1	46.9	0

	QSNTVTDAPQGITLTLTK	6		5.41	1901.2	3	1889.9	406.4	630.6	59.7	0
	SALEKFQTANTALNK	4		5.30	1944.8	2	1637.4	351.5	819.2	51.6	1
	YKAQFTQLDTMMSK	4		5.26	2103.5	2	1692.4	-374.1	846.7	57.2	1
	AQFTQLDTMMSK	5		5.18	1578.7	2	1402.6	667.0	701.8	61.4	0
	DAINDADSGIAASIVK	7		5.15	3488.5	2	1560.4	-222.0	780.7	60.8	0
	FQTANTALNKADLFK	3		5.12	1551.2	2	1683.5	339.8	842.2	56.2	1
	QYLSVSNSIDETVAR	13		5.09	1235.3	2	1682.3	-328.7	841.6	60.5	0
	YKAQFTQLDTmmSK	2	M11(Oxidation); M12(Oxidation)	4.68	2788.8	3	1725.1	47.7	575.7	50.5	1
	AQFANSGSNSAFK	12		4.47	1432.6	2	1330.0	448.4	665.5	47.8	0
	ELLVGDGKETGITTK	9		4.43	811.2	2	1561.4	-233.0	781.2	51.6	1
	VTDATVTVTKDDTK	5		4.40	1513.9	2	1494.3	-224.1	747.7	39.5	1
	LNVNGIDIER	10		4.05	1324.9	2	1144.0	604.9	572.5	58.4	0
	AQFTQLDTmmSK	3	M9(Oxidation); M10(Oxidation)	4.04	1449.7	2	1434.1	321.6	717.6	48.1	0
	AQFTQLDTmMSK	3	M9(Oxidation)	3.99	1525.4	2	1417.2	-330.5	709.1	52.2	0
	YKAQFTQLDTmMSK	1	M11(Oxidation)	3.80	1671.3	2	1708.2	-468.0	854.6	52.2	1
	LDKGDTSMEAIR	3		3.76	1472.1	2	1337.9	1033.1	669.4	44.4	1
	LDKGDTSmEAIR	4	M8(Oxidation)	3.68	1227.8	3	1352.8	211.0	451.6	37.7	1
	KVTDATVTVTK	6		3.64	1341.1	2	1162.8	-451.4	581.9	38.0	1
	FQTANTALNK	7		3.51	559.4	2	1109.0	667.6	555.0	45.8	0
	NGALLGDSVVR	5		3.45	1484.2	2	1100.9	-348.2	550.9	51.9	0
	STVASSTTEDLK	14		3.41	1314.9	2	1239.0	-237.1	620.0	39.8	0
	YTAVEPGEEASDK	3		3.34	1276.8	2	1396.1	-260.0	698.5	42.5	0
	VSTTAGAAAGTYK	13		3.33	1593.0	2	1198.0	-246.4	599.5	40.1	0
	VTDATVTVTK	1		3.23	889.4	2	1034.6	-599.3	517.8	39.6	0
	VLKDNTAAAR	6		3.22	831.2	2	1058.8	-425.6	529.9	32.4	1
	GDTSMEAIR	1		2.68	347.3	2	979.4	-675.5	490.2	44.7	0
	IDDDKLTK	1		2.67	502.8	3	948.6	557.9	316.9	37.7	1
	ELLVGDGK	3		2.51	312.1	1	830.4	-618.9	830.4	41.6	0
	EQLGDTSVTSR	6		2.47	210.3	1	1194.4	974.3	1194.4	41.5	0
CBW18139	Type III secretion system effector protein SopA	55	20.46	11	11	14	782	86.7			
	HDFPVFLAAFNQQATQR	3		5.16	1863.8	2	1990.3	-464.0	995.7	71.2	0
	AGENVSTAQISPVELFR	1		4.92	1275.6	2	1818.6	-258.8	909.8	69.0	0
	FTYSTLSDVVcSNTK	1	C11(Carbamidomethyl)	4.88	1843.1	2	1722.3	-363.2	861.6	63.0	0
	KHIANQTcDYNYR	1	C8(Carbamidomethyl)	4.71	2311.8	3	1683.8	-50.8	561.9	40.8	1
	AGETLTAADRDFSNADFR	1		4.65	2035.8	3	1958.6	274.6	653.5	58.1	1
	YSSSAIFGTEHDSPPALR	2		3.94	1246.6	2	1935.0	-588.6	968.0	58.6	0

	KYFPSVLSSILPLAWA	1		3.90	869.4	2	1793.3	55.0	897.1	93.8	1
	ISSGAINFSTIPNQVK	1		3.18	568.0	2	1677.4	318.8	839.2	62.6	0
	KNISAEDVSK	1		3.08	877.5	2	1090.3	-827.4	545.7	37.7	1
	GYAEALMQK	1		3.05	1218.1	2	1010.6	-629.8	505.8	52.0	0
	ILPVLLDSFDR	1		2.93	783.3	2	1288.3	-171.6	644.7	73.5	0
CBW18962	SPI-1 Type III secretion system effector protein SipB	35	16.36	8	8	10	593	62.4			
	LMTLLGDVSLSQLESR	1		5.56	2486.2	2	1762.6	-240.3	881.8	78.1	0
	AMSSAVQQNADASR	1		4.05	1850.2	2	1436.0	-371.1	718.5	41.6	0
	AmSSAVQQNADASR	1	M2(Oxidation)	3.79	2247.2	2	1452.0	-358.3	726.5	36.7	0
	FAMDQIQQWLK	1		3.76	1380.5	2	1408.3	-295.3	704.6	75.0	0
	LAEAAFEGVR	2		3.61	1951.8	2	1063.2	37.9	532.1	53.2	0
	EALDKATDATVK	1		3.19	468.6	2	1262.0	-305.1	631.5	42.3	1
	NASEALADFMLAR	1		2.91	2030.2	2	1409.1	-328.1	705.1	71.9	0
	ITSGLGNVGSK	1		2.72	767.7	2	1033.0	-145.3	517.0	45.0	0
	MGLQTNALSK	1		2.71	710.8	2	1062.9	-363.3	531.9	50.1	0
CBW19351	Polynucleotide phosphorylase	30	11.39	7	7	8	711	77.0			
	EIMQVALNQAK	1		4.35	1484.0	2	1245.3	-163.6	623.1	56.0	0
	LHILGVMEQAINAPR	1		4.32	1747.5	3	1663.1	49.3	555.0	69.4	0
	WDWQPEAVNDALNAR	1		4.13	1095.9	2	1784.9	-550.9	893.0	68.7	0
	VTDYLQMGQEVPVK	1		3.67	1354.6	2	1606.5	-844.1	803.8	61.8	0
	EGRPSEGETLIAR	2		3.50	535.5	2	1415.0	-361.4	708.0	47.2	0
	REGRPSEGETLIAR	1		3.47	861.4	3	1572.1	210.9	524.7	44.9	1
	AAVAGIAMGLVK	1		3.41	1520.1	2	1102.1	654.3	551.6	63.2	0
CBW16874	Type III secretion system effector protein SlrP	30	10.33	6	6	8	765	86.8			
	GAFDSHLAELIMAGR	2		4.99	1899.9	2	1588.7	-111.7	794.8	72.6	0
	MLAFPAYADNIQYSR	1		4.48	1204.5	2	1760.3	-414.8	880.6	67.1	0
	VLFAMGDFSIVR	1		3.66	1769.6	2	1355.4	-186.6	678.2	75.9	0
	VTESEQASSASGSK	1		3.54	2092.1	2	1367.7	-513.3	684.4	33.5	0
	TTEVQMDAER	1		3.45	1106.6	2	1181.2	735.1	591.1	43.0	0
	EIFRLEQIESLAR	2		3.05	987.2	3	1604.9	37.3	535.6	69.4	1
CBW16256	Acetyltransferase component (E2) of pyruvate dehydrogenase	25	13.35	7	7	7	629	66.0			
	VPDIGTDEVEITEILVK	1		5.69	2609.0	2	1870.6	-265.6	935.8	78.4	0
	FGEVEEVELGR	1		3.59	927.0	2	1264.7	260.1	632.9	57.6	0
	FITIINNMLSDIR	1		3.49	2336.4	2	1550.2	-424.8	775.6	82.4	0
	KEAAPAAAPAAAAAK	1		3.38	987.7	2	1309.2	-253.9	655.1	40.1	1
	FNSSLSEDGQR	1		3.16	1183.9	2	1239.4	-689.1	620.2	44.9	0
	VIDGADGAR	1		2.77	514.6	2	874.0	12.3	437.5	34.2	0

	ISGANLSR	1		2.74	707.7	2	817.9	-66.8	409.4	40.6	0
CBW20124	Glycerol kinase	24	14.74	6	6	7	502	56.0			
	YIVALDQGTTSSR	1		4.13	2276.4	2	1411.4	-93.7	706.2	52.2	0
	SSEVYGQTNIGGK	1		4.03	1592.4	2	1339.9	-398.9	670.5	43.7	0
	ATLESIAYQTR	2		3.85	1320.4	2	1253.4	22.5	627.2	53.0	0
	AVVMDHDANIVSVSQR	1		3.22	889.4	3	1741.9	-66.3	581.3	54.4	0
	VHVTDYTNASR	1		3.07	1067.3	2	1262.9	-374.3	631.9	40.7	0
	TADIcEQLKR	1	C5(Carbamidomethyl)	3.05	1234.7	2	1234.3	-69.3	617.7	44.2	1
CBW18961	SPI-1 Type III secretion system effector protein SipC	22	11.00	4	4	6	409	43.0			
	IDKLTTESHSIK	1		3.83	1397.5	3	1372.7	132.7	458.3	42.4	1
	ANEVVQTQLR	1		3.80	1523.5	2	1157.9	-312.6	579.5	47.2	0
	SEQQISQVNNR	3		3.61	1457.8	2	1303.0	-284.7	652.0	39.9	0
	DILNSIGISSSK	1		3.44	1512.0	2	1234.1	-209.7	617.6	63.1	0
CBW16257	Dihydrolipoamide dehydrogenase	21	14.14	5	5	5	474	50.6			
	GISYETATFPWAASGR	1		5.48	1595.6	2	1715.7	461.9	858.3	65.0	0
	GVHEGHVAAEVIAGKK	1		4.56	1838.8	3	1602.6	-136.5	534.9	42.6	1
	YDAVLVAIGR	1		4.10	1855.2	2	1077.6	335.3	539.3	59.6	0
	AIASDcADGmTK	1	C6(Carbamidomethyl); M10(Oxidation)	3.29	1372.6	2	1255.8	-498.1	628.4	35.3	0
	ALAEHGIVFGEPK	1		3.10	802.4	3	1369.3	511.7	457.1	52.6	0
CBW19508	Elongation factor G	18	10.09	5	5	5	704	77.6			
	VYSGVVNSGDTVLNSVK	1		5.08	1759.4	2	1738.3	-373.5	869.6	60.1	0
	SGPLAGYPVVDLGVR	1		4.47	1212.9	2	1501.5	479.7	751.2	67.6	0
	IATDPFVGNLTFFR	1		3.09	1169.4	2	1598.4	-274.6	799.7	76.9	0
	AKVTDIEGK	1		2.90	865.3	2	961.2	117.7	481.1	38.1	1
	EFNVEANVGKPQVAYR	1		2.87	628.6	3	1822.3	143.4	608.1	56.7	0
CBW17216	Flagellar hook-associated protein 1 FlgK	18	6.69	3	3	5	553	59.1			
	LLNTGSLGGLLTFR	2		4.71	1866.4	2	1462.6	-70.5	731.8	74.5	0
	GAQNQSSGLTTR	1		3.35	1340.4	2	1220.1	-167.5	610.5	35.4	0
	TSSTTQANVVK	2		3.16	879.1	2	1136.2	-10.1	568.6	35.3	0
CBW17006	Formate acetyltransferase 1	16	7.89	4	4	4	760	85.0			
	TMAcGIAGLSVAADSLSAIK	1	C4(Carbamidomethyl)	5.59	1805.4	2	1937.5	125.0	969.3	74.9	0
	LREEIAEQHR	1		3.79	1505.4	3	1281.2	-148.6	427.7	39.3	1
	GAIPTQSVLTITSNVVYGK	1		3.51	1281.8	2	1948.8	-234.1	974.9	72.9	0
	VDDMAVDLVER	1		3.26	1260.1	2	1261.8	-493.2	631.4	59.4	0
CBW19923	ATP synthase beta subunit	13	10.00	4	4	4	460	50.3			
	YTLAGTEVSALLGR	1		4.29	1521.8	2	1451.6	-53.2	726.3	66.7	0

	VGLFGGAGVGK	1		3.31	1458.9	2	962.2	28.3	481.6	53.4	0
	GVQSILQR	1		2.84	886.9	2	901.8	859.5	451.4	49.8	0
	VYDALEVQNGNEK	1		2.82	1895.8	2	1479.0	-364.2	740.0	49.7	0
CBW17950	Chemotaxis protein CheA	11	5.96	3	3	3	671	73.0			
	ILLPLTLAILDGMSVR	1		4.49	2702.8	2	1725.6	-334.5	863.3	94.6	0
	NSLDHGIEMPEKR	1		3.84	844.5	3	1526.5	-136.8	509.5	48.8	1
	GEYLPLPVELWK	1		2.88	513.4	2	1347.6	-14.0	674.3	79.1	0
CBW17217	Flagellar hook-associated protein 3 FlgL	10	6.94	2	2	3	317	34.2			
	TPVEGNNVEKEK	2		3.47	1039.8	2	1344.0	-382.3	672.5	37.2	1
	SVTQQVDSAR	1		2.97	675.7	2	1091.0	-189.7	546.0	40.2	0
CBW18409	Phosphate acetyltransferase	6	4.34	2	2	2	714	77.2			
	AGGDAPDQTTTIVR	1		3.39	1013.5	2	1402.1	-281.1	701.6	46.6	0
	ATVFIFPDLNTGNTTYK	1		2.89	357.7	2	1902.3	-427.1	951.7	73.0	0
	AAYAVDDSGKR	1		2.67	1344.1	2	1152.6	-582.3	576.8	37.9	1
