# Supplementary material for: Endoribonuclease-mediated control of hns mRNA stability constitutes a key regulatory pathway for Salmonella Typhimurium pathogenicity island 1 expression
Source: PLoS Pathog. 2021 Feb 1;17(2):e1009263. doi: 10.1371/journal.ppat.1009263 (PMC7877770; doi:10.1371/journal.ppat.1009263)
Supplement: S2 Table — (PDF) [file ppat.1009263.s011.pdf]

S2 Table. Mass spectrometric data obtained from the in-gel digestion of gel bands containing the majority of T3SS effector proteins and flagellar proteins in supernatants of *rng*-deleted cells of *Salmonella* Typhimurium strain SL1344 by filtering protein identities with 2 or more unique peptides.

Accession	Description	Score	Coverage	#Unique Peptides	#Peptides	# PSMs	# AAs	MW [kDa]			
CBW17983	Flagellin FliC	2279	52.32	16	25	565	495	51.6			
	Sequence	# PSMs	Modifications	XCorr	SpScore	Charge	MH+ [Da]	ΔM [ppm]	m/z [Da]	RT [min]	#Missed cleavage
	LNEIDRVSGQTQFNGVK	60		6.38	2201.2	3	1907.0	493.4	636.4	52.3	1
	IEDSDYATEVSNMSR	10		6.28	2497.2	2	1719.0	683.4	860.0	54.4	0
	INSAKDDAAGQAIANR	47		5.99	1546.9	2	1616.2	288.1	808.6	47.5	1
	SRIEDSDYATEVSNMSR	59		5.90	1970.2	3	1961.8	386.3	654.6	56.9	1
	AQPDLAEAAATTTENPLQK	13		5.79	2746.3	2	1969.5	-341.5	985.2	106.4	0
	QINSQTLGLDTLNVQQK	47		5.58	1313.4	3	1901.8	365.3	634.6	60.4	0
	LGGADGKTEVVSIGGK	45		5.55	1402.9	3	1488.7	35.0	496.9	47.9	1
	IDAALAQVDTLR	60		5.49	1835.3	2	1287.4	758.1	644.2	57.2	0
	SRIEDSDYATEVSNmSR	21	M15(Oxidation)	5.42	1322.5	3	1977.8	381.4	659.9	52.1	1
	IEDSDYATEVSNmSR	7	M13(Oxidation)	5.39	2349.9	2	1733.2	-356.8	867.1	52.2	0
	AALTAAGVTGTASVVK	10		4.63	2998.4	2	1417.4	-177.6	709.2	58.0	0
	IDGDLKFDDTTGK	2		4.59	1416.7	2	1425.0	-341.5	713.0	50.5	1
	VGDDYYSATQNK	4		4.54	1820.3	2	1361.0	-286.7	681.0	42.7	0
	ASATGLGGTDQKIDGDLK	3		4.50	1310.0	2	1747.4	-313.4	874.2	52.6	1
	VSGQTQFNGVK	39		4.42	1113.4	2	1165.6	306.0	583.3	40.1	0
	DDAAGQAIANR	35		4.14	1480.0	2	1102.2	49.9	551.6	44.2	0
	NVQVANADLTEAK	1		3.90	2236.0	2	1373.2	-199.1	687.1	49.2	0
	TYAASKAEGHNFK	1		3.90	2128.8	2	1423.8	-523.1	712.4	40.9	1
	DGSISINTTK	7		3.66	840.5	2	1036.9	732.2	518.9	46.8	0
	SQSALGTAIER	39		3.62	1459.3	2	1133.4	176.5	567.2	102.6	0
	FDDTTGKYYAK	1		3.52	1328.8	2	1308.8	-444.9	654.9	46.6	1
	ASATGLGGTDQK	3		3.51	1243.2	2	1105.9	-255.7	553.5	37.3	0
	SDLGAVQNR	36		3.47	1367.6	2	960.6	639.3	480.8	101.6	0
	FTANIKGLTQASR	1		3.28	1094.4	2	1408.7	801.9	704.9	56.0	1
	mSYTDNNGK	9	M1(Oxidation)	2.90	510.5	2	1046.7	545.5	523.8	27.0	0
	TEVVSIGGK	1		2.76	676.4	2	890.0	-19.4	445.5	47.1	0
	DGYYEVSVDK	1		2.75	940.3	2	1174.8	-346.2	587.9	50.5	0
	MSYTDNNGK	3		2.59	404.3	1	1031.4	1268.0	1031.	36.5	0

									4		
CBW18959	SPI-1 Type III secretion system effector protein SipA	1631	60.44	48	48	458	685	73.9			
	AGPAYESYLNKPGVDR	16		5.77	1876.4	2	1737.6	-164.1	869.3	56.6	0
	TQATNLAANLSAVR	41		5.39	2799.0	2	1431.1	334.1	716.0	58.9	0
	GTTGETTSFDEVDGVTSK	56		5.21	1999.6	2	1831.9	26.9	916.5	54.4	0
	METLKEVITHHPQK	3		5.20	1573.6	2	1691.4	-375.7	846.2	52.5	1
	SIIGKPVQATVHGVDDNK	3		4.99	1540.1	3	1879.5	190.6	627.2	50.3	0
	LENLEKQLLDIIK	4		4.94	2443.0	2	1569.4	-328.0	785.2	80.1	1
	SNLTHLVDKAAAK	11		4.86	1752.6	2	1368.2	-311.8	684.6	50.5	1
	NVFAQIPADKLPDPK	4		4.84	783.4	2	1653.3	-362.3	827.2	62.2	1
	VGAEYTAQIIKDGLK	7		4.69	1800.9	2	1606.4	-317.5	803.7	61.3	1
	TDTLQSDTTVITGNK	4		4.60	1815.1	2	1594.2	-350.2	797.6	53.9	0
	HLEMQTLIPLLLR	19		4.53	2140.0	2	1577.7	-157.3	789.4	84.9	0
	HIDNSNHDNSRK	48		4.51	780.3	2	1437.5	11.9	719.2	16.2	1
	DAEALKEVFTNSNNVAGK	2		4.50	1685.3	2	1906.6	-773.6	953.8	60.6	1
	LAEIALQFAR	47		4.49	2039.0	2	1132.1	-188.9	566.6	64.7	0
	HIDNSNHDNSR	17		4.44	1090.0	2	1310.5	924.8	655.8	9.2	0
	EVFTNSNNVAGKK	4		4.35	861.9	2	1408.2	-263.0	704.6	42.6	1
	KAIMEFAGLFR	7		4.30	1675.0	2	1282.9	-570.9	641.9	70.0	1
	LPDPKIPEPAAGPVPDGGK	1		4.28	1079.5	3	1856.4	164.0	619.5	58.1	1
	KALEPDTSTPFPVR	2		4.18	841.3	2	1558.4	-245.0	779.7	56.5	1
	HLEmQTLIPLLLR	14	M4(Oxidation)	4.15	1231.1	2	1593.1	-577.9	797.0	74.0	0
	GPQLEDFPALIK	3		4.15	1548.1	2	1329.3	595.2	665.2	70.5	0
	EKLAEIALQFAR	4		4.00	1151.3	2	1389.3	-277.7	695.1	68.9	1
	LTQEQGTSVGR	13		3.98	1367.5	2	1175.6	-554.1	588.3	40.6	0
	EKSAFGPWLPETK	2		3.97	880.2	2	1490.3	-271.0	745.7	62.0	1
	VITTVDGLHMQR	4		3.97	1101.8	2	1370.4	-164.4	685.7	54.1	0
	mETLKEVITHHPQK	3	M1(Oxidation)	3.94	2334.5	3	1708.2	111.6	570.1	49.4	1
	AVEALDMcHQK	4	C8(Carbamidomethyl)	3.85	1324.3	2	1302.0	-398.1	651.5	46.8	0
	IPEPAAGPVPDGGKK	2		3.85	1681.2	2	1433.3	-242.1	717.2	47.4	1
	IPEPAAGPVPDGGK	5		3.85	853.8	1	1306.6	850.1	1306.6	50.3	0
	AIMEFAGLFR	8		3.77	1551.7	2	1156.3	763.0	578.6	75.8	0
	SALNATSDSPEAK	10		3.75	1200.5	2	1291.2	-91.0	646.1	43.7	0
	AQcSDIDKHPELK	7	C3(Carbamidomethyl)	3.74	886.4	2	1541.2	-369.8	771.1	43.8	1
	AVEALDmcHQK	4	M7(Oxidation); C8(Carbamidomethyl)	3.72	1353.5	2	1320.0	1095.5	660.5	47.7	0
	AFDGLRAEILPNDTIK	7		3.71	730.4	2	1773.3	-437.1	887.1	65.3	1
	AImEFAGLFR	5	M3(Oxidation)	3.69	910.5	2	1171.1	-258.5	586.1	75.2	0

	QLLDIIKNNTGGELSK	4		3.68	600.4	2	1744.4	229.8	872.7	62.5	1
	VITTVDGLHmQR	3	M10(Oxidation)	3.59	1429.4	2	1386.2	-321.5	693.6	49.2	0
	EVFTNSNNVAGK	3		3.53	1442.2	2	1281.7	1030.9	641.4	46.6	0
	EVITHHPQKEK	7		3.42	484.0	2	1346.2	-269.5	673.6	34.4	1
	SNLTHLVDK	7		3.39	1144.6	2	1027.0	-118.4	514.0	47.5	0
	VGAEYTAQIIK	2		3.30	1633.7	2	1193.2	-161.1	597.1	54.7	0
	SAFGPWLPETK	9		3.25	787.9	2	1233.1	-230.5	617.1	65.2	0
	ESATATLSGEIK	11		3.25	1460.4	2	1207.6	260.6	604.3	95.7	0
	KAImEFAGLFR	1	M4(Oxidation)	3.07	1576.4	3	1298.8	-593.4	433.6	63.8	1
	TFIDNSQR	5		3.02	582.1	2	981.7	682.9	491.4	44.3	0
	cGKDAEALK	5	C1(Carbamidomethyl)	2.83	491.2	3	992.5	377.9	331.5	30.9	1
	AEAKLENLEK	2		2.83	576.5	2	1145.1	-171.4	573.1	45.3	1
	ETFLGVIRK	1		2.76	321.5	2	1062.7	-521.6	531.9	57.5	1
	LTQEQGTSVGREAR	1		2.74	583.6	2	1532.1	-346.3	766.6	40.3	1
	HINNSRSHVDNSQR	1		2.65	494.0	3	1666.2	863.8	556.1	34.7	1
	EVITHHPQK	1		2.65	302.1	2	1088.8	-364.4	544.9	31.9	0
	RAFDGLR	2		2.63	712.3	2	834.4	-614.6	417.7	44.2	1
	SALNATSDSPEAKTLLMK	1		2.62	768.2	3	1878.1	-33.9	626.7	57.8	1
	AEILPNDTIK	1		2.60	433.3	1	1115.5	1132.0	1115.5	53.7	0
CBW18855	Flagellin FljB	1402	18.77	10	10	356	506	52.5			
	LNEIDRVSGQTQFNGVK	60		6.38	2201.2	3	1907.0	493.4	636.4	52.3	1
	IEDSDYATEVSNMSR	10		6.28	2497.2	2	1719.0	683.4	860.0	54.4	0
	INSAKDDAAGQAIANR	47		5.99	1546.9	2	1616.2	288.1	808.6	47.5	1
	SRIEDSDYATEVSNMSR	59		5.90	1970.2	3	1961.8	386.3	654.6	56.9	1
	SRIEDSDYATEVSNmSR	21	M15(Oxidation)	5.42	1322.5	3	1977.8	381.4	659.9	52.1	1
	IEDSDYATEVSNmSR	7	M13(Oxidation)	5.39	2349.9	2	1733.2	-356.8	867.1	52.2	0
	IDAALAQVDALR	2		4.70	1781.7	2	1256.6	147.4	628.8	59.5	0
	VSGQTQFNGVK	39		4.42	1113.4	2	1165.6	306.0	583.3	40.1	0
	DDAAGQAIANR	35		4.14	1480.0	2	1102.2	49.9	551.6	44.2	0
	SQSALGTAIER	39		3.62	1459.3	2	1133.4	176.5	567.2	102.6	0
	SDLGAVQNR	36		3.47	1367.6	2	960.6	639.3	480.8	101.6	0
	FTANIKGLTQASR	1		3.28	1094.4	2	1408.7	801.9	704.9	56.0	1
CBW17984	Flagellar hook associated protein FliD	571	58.89	26	26	152	467	49.8			
	INVTQLAAAQSLATK	12		7.29	2952.0	2	1530.5	457.9	765.7	61.3	0
	LNNTSSYLTQQFTAMNK	4		5.75	1890.0	2	1961.4	-398.7	981.2	62.1	0
	DAINDADSGIAASIVK	7		5.59	3176.2	2	1560.4	-224.3	780.7	60.2	0
	TMAEIGITQDGTSGK	7		5.42	1819.7	2	1510.0	212.4	755.5	53.8	0
	TTFATTKEQLGDTSVTSR	3		5.41	1432.6	3	1944.2	38.0	648.7	50.6	1

	QSNTVTDAPQGITLTLTK	6		5.37	1678.1	3	1889.4	153.8	630.5	59.8	0
	LNNTSSYLTQQFTAmNK	2	M15(Oxidation)	4.91	1470.2	2	1977.3	-433.5	989.2	57.1	0
	TmAEIGITQDGTSGK	6	M2(Oxidation)	4.71	2150.4	2	1525.1	-356.8	763.1	47.0	0
	YKAQFTQLDTMMSK	2		4.66	2115.9	2	1692.2	-481.0	846.6	57.2	1
	QYLSVSNSIDETVAR	10		4.60	1346.5	2	1683.1	171.4	842.1	57.5	0
	ELLVGDGKETGITTK	5		4.57	990.6	2	1561.2	-348.4	781.1	53.6	1
	AQFTQLDTmMSK	2	M9(Oxidation)	4.45	1477.9	2	1419.1	1025.7	710.1	55.6	0
	FQTANTALNKADLFK	1		4.26	841.2	2	1682.2	-430.0	841.6	56.7	1
	AQFANSGSNSAFK	12		4.24	1593.0	2	1329.6	141.1	665.3	44.6	0
	AQFTQLDTMMSK	4		4.24	1527.8	2	1401.4	-212.9	701.2	58.7	0
	VTDATVTVTKDDTK	2		4.09	1539.2	2	1494.0	-409.2	747.5	43.2	1
	LDKGDTSmEAIR	3	M8(Oxidation)	3.75	1245.1	3	1352.5	-27.4	451.5	37.4	1
	LNVNGIDIER	8		3.74	1248.3	2	1143.7	376.5	572.4	55.4	0
	KVTDATVTVTK	4		3.64	1121.0	2	1164.1	623.0	582.5	39.3	1
	LDKGDTSMEAIR	2		3.53	1276.9	2	1337.1	459.3	669.1	44.6	1
	STVASSTTEDLK	10		3.51	1596.5	2	1239.0	-242.2	620.0	40.4	0
	YTAVEPGEEASDK	3		3.44	1107.7	2	1396.3	-125.0	698.6	44.7	0
	VTDATVTVTK	3		3.39	958.9	2	1035.7	537.9	518.4	43.3	0
	VSTTAGAAAGTYK	10		3.39	1514.7	2	1197.8	-461.6	599.4	46.5	0
	NGALLGDSVVR	3		3.34	1459.0	2	1100.9	-346.7	550.9	51.9	0
	AQFTQLDTmmSK	2	M9(Oxidation);, M10(Oxidation)	3.12	1390.0	2	1432.8	-569.9	716.9	50.7	0
	FQTANTALNK	8		3.12	814.6	2	1107.6	-557.8	554.3	42.2	0
	VLKDNTAAAR	5		3.12	830.2	2	1058.9	-261.4	530.0	32.5	1
	EQLGDTSVTSR	2		2.74	936.0	2	1193.1	-161.5	597.0	41.2	0
	IDDDKLTK	2		2.63	1053.4	2	947.4	-682.7	474.2	38.0	1
	ELLVGDGK	2		2.59	314.3	1	830.5	-596.5	830.5	41.7	0
CBW20124	Glycerol kinase	45	24.50	11	11	13	502	56.0			
	DVLEAMQADSGIR	1		4.69	2841.9	2	1405.3	-224.7	703.1	62.2	0
	ADISSDQIAAIGITNQR	1		4.51	1761.6	2	1773.4	-328.5	887.2	59.5	0
	YIVALDQGTTSSR	1		4.23	2052.9	2	1412.0	305.5	706.5	55.6	0
	MLDVLDIPR	1		3.69	1422.4	2	1072.1	-209.6	536.5	65.1	0
	SSEVYGQTNIGGK	2		3.61	1349.7	2	1339.7	-511.3	670.4	43.8	0
	AVVMDHDANIVSVSQR	2		3.58	892.1	3	1741.5	-243.4	581.2	51.8	0
	ATLESIAYQTR	1		3.46	1170.4	2	1253.5	93.9	627.3	53.1	0
	KSSEVYGQTNIGGK	1		3.10	420.4	3	1468.6	22.2	490.2	39.7	1
	VHVTDYTNASR	1		2.91	1205.2	2	1262.5	-682.5	631.7	40.9	0
	TADIcEQLKR	1	C5(Carbamidomethyl)	2.86	1106.3	2	1234.1	-228.2	617.6	44.3	1
	AMAWEEHDK	1		2.70	997.8	2	1116.9	-298.1	559.0	39.9	0
CBW17006	Formate acetyltransferase 1	43	19.21	12	12	13	760	85.0			

	TMLYAINGGVDEK	1		4.40	1895.0	2	1412.1	371.3	706.6	58.6	0
	RAGAPFGPGANPMHGR	1		4.29	2027.7	3	1594.7	559.4	532.2	49.3	1
	SGVLTGLPDAYGR	1		3.84	846.0	2	1307.7	982.8	654.4	60.7	0
	LREEIAEQHR	1		3.71	1142.8	3	1281.5	62.5	427.8	40.0	1
	VALYGIDYLMK	1		3.40	1347.9	2	1286.6	53.0	643.8	71.8	0
	GAIPTQSVLTITSNVVYGK	2		3.28	776.6	3	1948.7	-292.6	650.2	73.6	0
	VDDMAVDLVER	1		3.09	1235.5	2	1261.9	-389.4	631.5	60.3	0
	SQNGAAMSFGR	1		2.99	478.6	2	1127.1	788.4	564.1	48.6	0
	GDWQNEVNVR	1		2.86	1352.1	2	1216.5	-611.2	608.8	53.3	0
	IVGLQTEAPLKR	1		2.79	765.0	2	1325.7	95.8	663.4	53.2	1
	VSTFLDAYIER	1		2.69	1097.3	2	1313.8	-549.4	657.4	67.8	0
	EQQQDVITR	1		2.66	399.8	2	1118.0	701.7	559.5	40.6	0
CBW16257	Dihydrolipoamide dehydrogenase	41	24.89	9	9	10	474	50.6			
	YNTLGGVcLNVGcIPSK	1	C8(Carbamidomethyl); C13(Carbamidomethyl)	5.71	1835.1	2	1853.2	-11.8	927.1	63.6	0
	GISYETATFPWAASGR	1		5.08	1459.1	2	1715.4	323.8	858.2	65.0	0
	GVHEGHVAAEVIAGKK	1		5.00	2139.6	3	1603.0	93.2	535.0	42.4	1
	VITQLTGGLAGMAK	1		4.89	2230.6	2	1361.0	232.3	681.0	59.7	0
	YDAVLVAIGR	1		4.44	1914.9	2	1077.6	311.3	539.3	59.7	0
	AIASDcADGmTK	1	C6(Carbamidomethyl); M10(Oxidation)	3.66	1424.4	2	1255.8	-497.7	628.4	34.9	0
	VWDSTDALELK	1		3.44	1055.5	2	1277.4	-48.0	639.2	59.3	0
	KFNLMLETK	1		3.24	929.1	2	1124.2	-139.9	562.6	53.9	1
	ALAEHGIVFGEPK	2		3.16	551.4	2	1368.2	-298.6	684.6	56.8	0
CBW18962	SPI-1 Type III secretion system effector protein SipB	36	18.04	9	9	10	593	62.4			
	LMTLLGDVSLSQLESR	1		5.08	2226.8	2	1763.1	31.4	882.1	79.0	0
	NASEALADFMLAR	1		4.83	2213.7	2	1410.5	637.5	705.8	72.5	0
	AmSSAVQQNADASR	1	M2(Oxidation)	4.33	2296.8	2	1452.1	-315.1	726.5	37.0	0
	AMSSAVQQNADASR	1		3.89	1817.9	2	1436.0	-389.4	718.5	42.0	0
	LAVWQAMIESQK	1		3.33	1772.0	2	1404.4	-229.7	702.7	69.9	0
	FAMDQIQQWLK	1		3.32	1360.8	2	1408.2	-305.8	704.6	75.7	0
	LAEAAFEGVRK	1		3.20	1525.5	2	1190.1	-1054.3	595.6	50.1	1
	SAEFQEETR	1		2.88	1169.4	2	1096.8	-288.6	548.9	41.7	0
	ITSGLGNVGSK	1		2.82	966.0	2	1033.0	-212.6	517.0	45.4	0
	MGLQTNALSK	1		2.68	694.2	2	1062.3	-867.8	531.7	50.8	0
CBW19508	Elongation factor G	33	14.91	8	8	9	704	77.6			
	VYSGVVNSGDTVLNSVK	1		5.19	1776.4	2	1738.4	-296.8	869.7	60.9	0
	IATDPFVGNLTFFR	2		4.50	1228.9	2	1599.5	389.3	800.2	78.1	0

	SGPLAGYPVVDLGVR	1		3.72	1240.8	2	1500.3	-310.7	750.6	68.3	0
	MEFPEPVISIAVEPK	1		3.62	1045.4	2	1686.9	-71.4	843.9	73.9	0
	IVQMHANKR	1		3.32	1143.7	2	1097.0	-275.5	549.0	33.3	1
	EFNVEANVGKPQVAYR	1		3.28	582.2	3	1822.6	284.8	608.2	57.1	0
	GQESEVTGVK	1		3.00	979.8	2	1034.9	808.2	518.0	39.4	0
	AKVTDIEGK	1		2.94	861.8	2	961.9	819.2	481.4	38.7	1
CBW19351	Polynucleotide phosphorylase	33	15.47	10	10	10	711	77.0			
	WDWQPEAVNDALNAR	1		4.55	1176.4	2	1785.2	-398.6	893.1	69.1	0
	GETQALVTATLGTAR	1		3.89	1414.7	2	1489.3	-233.2	745.2	61.0	0
	EGRPSEGETLIAR	1		3.61	608.8	2	1415.2	-256.6	708.1	47.7	0
	EIMQVALNQAK	1		3.34	1347.6	2	1244.6	-692.6	622.8	56.3	0
	REGRPSEGETLIAR	1		3.26	212.8	2	1571.2	-315.1	786.1	46.1	1
	DGISALQMDIK	1		3.24	1119.7	2	1191.0	-304.6	596.0	62.7	0
	GVLAVMPDMDKFPYTVR	1		2.94	850.3	3	1940.7	203.9	647.6	72.0	1
	VAALAESR	1		2.81	1150.2	2	816.5	-546.1	408.7	39.6	0
	IAATDGEKAK	1		2.80	748.9	2	1003.6	-497.3	502.3	30.5	1
	GDISEFAPR	1		2.76	1268.2	2	991.9	-158.7	496.5	53.0	0
CBW18537	NADP-dependent malate dehydrogenase	32	15.55	10	10	10	759	82.3			
	VALLSHSNFGSSNSLSASK	1		4.40	1463.1	3	1907.1	-17.0	636.4	57.0	0
	GALDVGATAINEEMK	1		4.33	1375.9	2	1519.2	-364.9	760.1	61.0	0
	RVVLPEGEEAR	1		3.45	1037.7	2	1255.1	-220.2	628.1	47.1	1
	TNLFMKPIFSQAR	1		3.44	576.3	2	1555.3	912.3	778.1	64.7	0
	IQVSPTKPLATQR	1		3.01	828.1	2	1439.2	-352.8	720.1	49.1	0
	AAYAVDDSGKR	1		2.92	1193.9	2	1152.9	-272.6	577.0	38.4	1
	RGITQEQAQR	1		2.88	653.4	2	1186.8	-399.3	593.9	34.9	1
	GITQEQAQR	1		2.66	393.0	2	1032.0	837.4	516.5	35.8	0
	AMIGNHTAIGAIMVQR	1		2.62	754.1	3	1684.4	198.3	562.1	60.7	0
	GREPNMAETK	1		2.61	613.7	3	1133.7	373.1	378.6	36.3	1
CBW17380.2	Phosphoenolpyruvate synthase	31	13.90	9	9	9	806	88.8			
	DIFSLTNEEVQELAK	1		4.60	2063.0	2	1736.3	-330.8	868.7	77.8	0
	AFDFAcLPNEGVGLAR	1	C6(Carbamidomethyl)	4.42	1267.1	2	1737.4	-336.3	869.2	70.7	0
	LTEGIATLGAAFYPK	1		4.15	2101.6	2	1552.5	-175.3	776.8	71.1	0
	GVALSAGVQR	1		3.54	1569.7	2	957.9	-190.4	479.5	47.3	0
	NDMGLTNVEIMIPFVR	1		3.49	1358.0	2	1849.7	-268.8	925.4	82.8	0
	HVFASLFNDR	1		2.96	642.5	2	1206.5	114.1	603.7	59.9	0
	MVYAATQEHGK	1		2.91	1300.8	2	1236.1	573.7	618.6	39.8	0
	TcHAAIIAR	1	C2(Carbamidomethyl)	2.74	1111.8	2	1012.8	-448.6	506.9	41.1	0
	VMMNVGNPDR	1		2.67	962.3	2	1132.8	-512.5	566.9	48.8	0
CBW16998	Anaerobic dimethyl sulfoxide	31	13.27	8	8	9	814	90.3			

	reductase chain A precursor										
	TPEWAAQITGIPADR	1		5.12	1576.7	2	1626.3	-320.5	813.6	65.5	0
	ISWDEAYDIIATNMQR	2		4.13	1351.7	2	1926.9	-130.8	964.0	83.5	0
	LGVEQQFTEGR	1		3.60	1314.7	2	1265.1	598.9	633.1	53.6	0
	MSGGGVTYYLEQAR	1		3.58	1396.4	2	1532.1	-435.9	766.5	62.3	0
	VHSTYGNVDVLK	1		3.16	566.4	2	1332.1	-316.7	666.5	51.8	0
	THEILQDDKK	1		3.16	432.4	3	1226.5	-668.1	409.5	36.1	1
	LVVLFGNNPGETR	1		2.85	1395.8	2	1416.2	-275.8	708.6	62.3	0
	DFREDPQANPLTTPSGK	1		2.64	627.7	3	1873.6	-244.6	625.2	54.2	1
CBW18409	Phosphate acetyltransferase	31	16.25	9	9	9	714	77.2			
	GIATcVLLGNPDEINR	1	C5(Carbamidomethyl)	4.84	1383.6	2	1742.3	-406.5	871.6	64.4	0
	DAEVVLVEGLVPTRK	1		4.15	1345.8	2	1627.2	801.2	814.1	67.2	1
	ATVFIFPDLNTGNTTYK	1		3.63	341.3	2	1903.4	133.7	952.2	73.4	0
	HLNATIINEGDIK	1		3.44	1418.2	2	1437.5	-756.4	719.3	55.0	0
	NTNITGVIINK	1		3.38	1000.4	2	1187.1	-210.3	594.1	55.6	0
	AGGDAPDQTTTIVR	1		3.33	898.1	2	1402.0	-331.3	701.5	47.0	0
	LSVFKPIAQPR	1		2.73	601.8	2	1255.7	-669.3	628.4	56.1	0
	RVVLPEGDEPR	1		2.71	676.6	3	1266.8	-474.0	422.9	48.7	1
	RLSPPAFR	1		2.67	708.8	2	943.8	-323.4	472.4	50.2	1
CBW18139	Type III secretion system effector protein SopA	26	11.51	6	6	7	782	86.7			
	HDFPVFLAAFNQQATQR	2		4.75	2144.9	3	1990.8	-211.4	664.3	72.5	0
	YSSSAIFGTEHDSPPALR	1		4.71	1259.9	2	1936.0	-30.1	968.5	59.4	0
	KYFPSVLSSILPLAWA	1		3.81	755.6	2	1792.5	-360.5	896.8	94.6	1
	LATIFDPLLPEGK	1		3.26	1157.0	2	1414.5	-146.5	707.7	73.6	0
	AGENVSTAQISPVELFR	1		2.66	1389.5	2	1817.6	-792.5	909.3	69.5	0
	LIDDASVSR	1		2.65	593.3	2	976.0	-55.5	488.5	45.3	0
CBW19030	Enolase	19	9.95	3	3	4	432	45.6			
	SNFGANAILAVSLANAK	2		5.68	2663.5	2	1661.5	-242.3	831.3	71.4	0
	FNQIGSLTETLAAIK	1		4.85	1642.2	2	1607.4	321.1	804.2	72.8	0
	DAGYTAVISHR	1		3.17	1335.2	2	1190.1	-191.6	595.5	50.6	0
CBW16256	Acetyltransferase component (E2) of pyruvate dehydrogenase	19	8.11	4	4	5	629	66.0			
	VPDIGTDEVEITEILVK	1		5.08	1875.6	2	1870.7	-249.5	935.8	79.5	0
	KEAAPAAAPAAAAAK	2		4.03	946.9	2	1309.2	-200.3	655.1	40.4	1
	FNSSLSEDGQR	1		3.78	901.2	2	1241.4	873.4	621.2	45.4	0
	ISGANLSR	1		2.67	727.3	2	817.7	-277.6	409.4	41.1	0
CBW18961	SPI-1 Type III secretion system effector protein SipC	17	10.51	4	4	5	409	43.0			
	SEQQISQVNNR	2		3.85	1399.6	2	1303.8	312.3	652.4	40.6	0

	ANEVVQTQLR	1		3.60	1464.6	2	1157.6	-581.1	579.3	47.6	0
	IDKLTTESHSIK	1		3.13	1497.0	3	1372.4	-98.6	458.1	43.5	1
	LGAEGVDSLK	1		2.98	1501.6	2	988.8	-361.1	494.9	49.9	0
CBW19923	ATP synthase beta subunit	17	12.17	5	5	5	460	50.3			
	DVLLFVDNIYR	1		4.03	1177.5	2	1368.6	766.3	684.8	74.6	0
	VIDLMcPFAK	1	C6(Carbamidomethyl)	3.50	1271.9	2	1194.4	-125.5	597.7	65.3	0
	VYDALEVQNGNEK	1		3.21	2279.9	2	1479.0	-429.7	740.0	49.6	0
	VALTGLTMAEK	1		3.11	1353.1	2	1134.5	93.0	567.7	55.5	0
	VGLFGGAGVGK	1		2.95	1302.5	2	961.4	-765.3	481.2	53.6	0
CBW17216	Flagellar hook-associated protein 1 FlgK	16	8.50	4	4	5	553	59.1			
	TSSTTQANVVK	1		3.52	803.4	2	1137.1	746.1	569.1	36.4	0
	GAQNQSSGLTTR	2		3.46	1326.3	2	1219.8	-360.9	610.4	35.0	0
	QLAAVPSSADPTR	1		3.12	601.6	2	1312.3	-860.5	656.7	50.1	0
	QIANLNDQISR	1		2.67	520.5	2	1272.1	-226.2	636.6	51.7	0
CBW18240	Periplasmic beta-glucosidase precursor	13	7.06	4	4	4	765	83.3			
	HGTAADPEDAVR	1		3.68	899.3	2	1238.5	-646.9	619.7	41.3	0
	LKIPLFFAYDVVHGQR	1		3.17	542.0	3	1904.2	-15.1	635.4	79.0	1
	YDMGLFNDPYSHLGPK	1		3.12	704.7	2	1854.6	-267.3	927.8	67.0	0
	TNITIPQSQR	1		2.88	713.9	2	1158.1	-160.2	579.6	49.1	0
CBW16874	Type III secretion system effector protein SlrP	12	6.14	4	4	4	765	86.8			
	VTESEQASSASGSK	1		3.44	1413.2	2	1368.1	-228.6	684.5	33.6	0
	IIVEYNPFSER	1		2.78	982.4	2	1367.2	-266.0	684.1	65.0	0
	LMSSVDYQGPR	1		2.76	833.2	2	1252.5	-722.0	626.8	52.5	0
	EAANREEAVQR	1		2.75	320.1	3	1273.2	-120.3	425.1	34.4	1
CBW18348	Ribonucleoside-diphosphate reductase 1 alpha chain	11	4.73	3	3	3	761	85.7			
	TLDELEELAILAVR	1		4.75	2957.0	2	1586.8	629.4	793.9	86.6	0
	HMDYGVQINK	1		3.37	933.0	2	1205.2	-174.5	603.1	49.5	0
	YSDGSANNLTHK	1		3.21	709.9	2	1307.2	-106.8	654.1	39.7	0
CBW17217	Flagellar hook-associated protein 3 FlgL	7	6.94	2	2	2	317	34.2			
	TPVEGNNVEKEK	1		3.7	799.8	2	1345.2	544.6	673.1	37.5	1
	SVTQQVDSAR	1		2.8	673.2	2	1090.9	-262.2	545.9	40.9	0
CBW20129	Heat shock protein	6	5.19	2	2	2	443	49.6			
	HLDALVADEDLSR	1		3.72	1188.9	2	1454.0	-395.2	727.5	53.5	0
	YRAEELAEER	1		2.76	905.7	2	1267.1	596.6	634.1	43.5	1
	VmAENLLFNGQDLK	1	M2(Oxidation)	2.59	862.9	2	1609.9	626.8	805.4	61.8	0
