# Supplementary material for: Endoribonuclease-mediated control of hns mRNA stability constitutes a key regulatory pathway for Salmonella Typhimurium pathogenicity island 1 expression
Source: PLoS Pathog. 2021 Feb 1;17(2):e1009263. doi: 10.1371/journal.ppat.1009263 (PMC7877770; doi:10.1371/journal.ppat.1009263)
Supplement: S3 Table — 1See METHOD DETAILS for detailed description scheme for plasmids. Abbreviation: Ampr, ampicillin resistance; Kmr, kanamycin resistance; Tcr, tetracycline resistance; Cmr, chloramphenicol resistance; Sptr, spectinomycin resistance; Strr, streptomycin resistance. (PDF) [file ppat.1009263.s012.pdf]

S3 Table. Bacterial strains and plasmids used in this study.

Strains or plasmids	Relevant characteristics	Reference
***Salmonella* Typhimurium**		
SL1344	*hisG46, rpsL*, Str^{r}	[1]
Δ*rng*	Same as SL1344, but *rng*	[2]
Δ*rnc*	Same as SL1344, but *rnc-14*::Tn*10,* Tc^{r}	This study
Δ*fnr*	Same as SL1344, but *fnr*::*cat*, Cm^{r}	This study
Δ*arcA*	Same as SL1344, but *arcA*::*cat*, Cm^{r}	This study
SL1344 hns-N_{20}	Same as SL1344, but containing a 20 bp artificial protospacer (N20) of the 5' UTR of *hns* gene	This study
hns A-31C	Same as SL1344, but A-31C of 5' UTR of *hns*	This study
hns A-9G	Same as SL1344, but A-9G of 5' UTR of *hns*	This study
hns U-8C	Same as SL1344, but T-8C of 5' UTR of *hns*	This study
Escherichia coli		
DH5α	F^{-}, *endA1*, *glnV44*, *thi-1*, *recA1*, *relA1*, *gyrA96*, *deoR*, *nupG*, *purB20*, φ80d*lacZ*ΔM15, Δ(*lacZYA-argF*)U169, hsdR17($r_K^- m_K^+$), λ^{-}	Laboratory strain
HT115	F^{-}, *mcrA*, *mcrB*, IN(*rrnD-rrnE*)1, λ^{-}, *rnc-14*::Tn*10,* Tc^{r}	[3]
N3433	*Hfr, lacZ43,* λ^{-}*, relA1, spoT1, thi-1*	[4]
N3433*rng*	Same as N3433, but *rng*	[2]
**Plasmids[1]**		
pACYC177	p15A *ori*, Amp^{r}, Km^{r}	[5]
pKD46	*repA101*(ts), *oriR101, bla,* P_{araB}-(*gam bet exo*), Amp^{r}	[6]
pKD3	*oriRγ*, *cat*, Δ(*phoB-phoR*)*580*, *galU95*, Δ*uidA3*::*pir*$^{+}$, Δ*endA*::FRT, Cm^{r}	[6]
pCP20	λ*cI1857*(ts), *repA101*(ts), *oriR101*, *bla*, *cat*, λp_{R-}, FLP, Amp^{r}, Cm^{r}	[7]
pCAT924	pBR322 *ori*, *cat* under *trp*^{c} promoter, Amp^{r}, Cm^{r}	[8]
pSt-rng	pACYC177 containing the *S.* Typhimurium *rng* gene, Amp^{r}	This study
pSt-rng-His	Same as pSt-rng, but C-terminal hexa-histidine tag, Amp^{r}	This study
pSt-hns (WT)-cat	pCAT924 containing the *S.* Typhimurium wild-type *hns::cat* fusion under *trp*^{c} promoter, Amp^{r}, Cm^{r}	This study
pSt-hns (A-31C)-cat	Same as pSt-hns (WT)-cat, but A-31C of 5'-UTR of *hns*	This study
pSt-hns (A-9G)-cat	Same as pSt-hns (WT)-cat, but A-9G of 5'-UTR of *hns*	This study
pSt-hns (U-8C)-cat	Same as pSt-hns (WT)-cat, but T-8C of 5'-UTR of *hns*	This study
pSt-rngP-cat	Same as pCAT924, but *cat* under *S.* Typhimurium *rng* promoter, Amp^{r}, Cm^{r}	This study
pSt-rncP-cat	Same as pCAT924, but *cat* under *S.* Typhimurium *rnc* promoter, Amp^{r}, Cm^{r}	This study
pSt-hnsP-cat	Same as pCAT924, but *cat* under *S.* Typhimurium *hns* promoter, Amp^{r}, Cm^{r}	This study
pCas	*repA101*(Ts), *kan*, P_{cas}-*cas9*, P_{araB}-*Red*, *lacI*^{q}, P_{trc}-sgRNA-*pMB1*, Km^{r}	[9] Addgene: 62225
pTargetF	*pMB1*, *aadA*, sgRNA-*pMB1*, Spt^{r}	[9] Addgene: 62226
pTargetF (Amp)	Same as pTargetF, but *aadA*::*bla*, Amp^{r}	This study
pTargetF (Amp)-original N_{20}	Same as pTargetF (Amp) containing a 20 bp native protospacer (N20) of the 5' UTR of *hns* gene, Amp^{r}	This study
pTargetF (Amp)-artificial N_{20}	Same as pTargetF (Amp) containing a 20 bp artificial protospacer (N20), Amp^{r}	This study

[1]See Methods for detailed description scheme for plasmids.

Abbreviation: Amp^{r}, ampicillin resistance; Km^{r}, kanamycin resistance; Tc^{r}, tetracycline

resistance; Cm^{r}, chloramphenicol resistance; Spt^{r}, spectinomycin resistance; Str^{r}, streptomycin resistance.

Supplemental References

1. Hoiseth SK, Stocker BA. Aromatic-dependent *Salmonella typhimurium* are non-virulent and effective as live vaccines. Nature. 1981;291(5812):238-9. Epub 1981/05/21. doi: 10.1038/291238a0. PubMed PMID: 7015147.

2. Song W, Kim YH, Sim SH, Hwang S, Lee JH, Lee Y, et al. Antibiotic stress-induced modulation of the endoribonucleolytic activity of RNase III and RNase G confers resistance to aminoglycoside antibiotics in *Escherichia coli*. Nucleic Acids Res. 2014;42(7):4669-81. Epub 2014/02/04. doi: 10.1093/nar/gku093. PubMed PMID: 24489121; PubMed Central PMCID: PMCPMC3985665.

3. Court DL. Control of messenger RNA stability. Belasco JG, Brawerman G, editors. New York: Academic Press, INC.; 1993.

4. Goldblum K, Apririon D. Inactivation of the ribonucleic acid-processing enzyme ribonuclease E blocks cell division. J Bacteriol. 1981;146(1):128-32. Epub 1981/04/01. PubMed PMID: 6163761; PubMed Central PMCID: PMCPMC217061.

5. Chang AC, Cohen SN. Construction and characterization of amplifiable multicopy DNA cloning vehicles derived from the P15A cryptic miniplasmid. J Bacteriol. 1978;134(3):1141-56. Epub 1978/06/01. PubMed PMID: 149110; PubMed Central PMCID: PMCPMC222365.

6. Datsenko KA, Wanner BL. One-step inactivation of chromosomal genes in *Escherichia coli* K-12 using PCR products. Proc Natl Acad Sci U S A. 2000;97(12):6640-5. Epub 2000/06/01. doi: 10.1073/pnas.120163297. PubMed PMID: 10829079; PubMed Central PMCID: PMCPMC18686.

7. Cherepanov PP, Wackernagel W. Gene disruption in *Escherichia coli*: Tc^R and Km^R cassettes with the option of Flp-catalyzed excision of the antibiotic-resistance determinant. Gene. 1995;158(1):9-14. Epub 1995/05/26. doi: 10.1016/0378-1119(95)00193-a. PubMed

PMID: 7789817.

8. Sim SH, Yeom JH, Shin C, Song WS, Shin E, Kim HM, et al. *Escherichia coli* ribonuclease III activity is downregulated by osmotic stress: consequences for the degradation of *bdm* mRNA in biofilm formation. Mol Microbiol. 2010;75(2):413-25. Epub 2009/12/01. doi: 10.1111/j.1365-2958.2009.06986.x. PubMed PMID: 19943899.

9. Jiang Y, Chen B, Duan C, Sun B, Yang J, Yang S. Multigene editing in the *Escherichia coli* genome via the CRISPR-Cas9 system. Appl Environ Microbiol. 2015;81(7):2506-14. Epub 2015/02/01. doi: 10.1128/AEM.04023-14. PubMed PMID: 25636838; PubMed Central PMCID: PMCPMC4357945.
