# Supplementary material for: Endoribonuclease-mediated control of hns mRNA stability constitutes a key regulatory pathway for Salmonella Typhimurium pathogenicity island 1 expression
Source: PLoS Pathog. 2021 Feb 1;17(2):e1009263. doi: 10.1371/journal.ppat.1009263 (PMC7877770; doi:10.1371/journal.ppat.1009263)
Supplement: S4 Table — (PDF) [file ppat.1009263.s013.pdf]

Table S4 Primers used in this study.

Primers	Sequence (5' - 3')
**Construction of deletion strains**	
rnc-1F	ATGAACCCCATCGTAATTAA
rnc-86R	TGAGTTAATGCCTGCTGCAA
St-fnr-del-F	CTTCTCCGGGATAGCTCAGACTTACGCGCTCACCAAAAAGGTGTAGGCTGGAGCTGCTTC
St-fnr-del-R	ACGATATGGCAGAAGATAACATCAATGGTTTAGCTGACGTCATATGAATATCCTCCTTA
St-arcA-del-F	ACTTCCTGTTTCGATTTAGTTGGCAATTTAGGTAGCAAACGTGTAGGCTGGAGCTGCTTC
St-arcA-del-R	AACTTACCGGCTGTTTTTACAGTTTGGCGCCTGGGCCGAACATATGAATATCCTCCTTA
St-fnr-con-F	GCGTAACGGCACTCTGAC
St-fnr-con-R	AGGAAGCGCTTCTGCCAGAT
St-arcA-con-F	GTTAATTTGCGACATGCATC
St-arcA-con-R	GCTTAGCGGAAGGGGGACAT
**Construction of pSt-rng and pSt-rng-His**	
rng-F-XhoI	GATCCTCGAGCTGGCTATGTCGCCAGCG
rng-R-BamHI	GATCGGATCCCGTCTCTCCTTGTCTGCC
rng-His-R-BamHI	TAGGATCCTTAATGATGATGATGATGATGCATCATTACGACGTCAAACTG
**Construction of pSt-hns-cat and pSt-hns-cat mutants**	
St-hns(WT)-cat F (NotI)	AAGCGGCCGCAACAAACCACCCCAATATAA
St-hns(MT)-cat F (NotI)	AAGCGGCCGCAACAANCCACCCCAATATAAGTTTGAGNNTACTACAATG (N-G,C, T or A)
St-hns-cat M R	GGTGGTATATCCAGTGATTTTTTTCTCTTCTCTTGCCTGCGCACGAAGAGTACG
St-hns-cat M F	CGTACTCTTCGTGCGCAGGCAAGAGAAGAGAAAAAAATCACTGGATATACCACC
cat-R (NcoI)	AATATTTGCCCATGGTGAAAACGGGGGCGAAGAAGTTGTC
**Construction of pSt-rngP-cat**	
St-rngP-cat F (NotI)	AAGCGGCCGCAATGCTGCGTTTGCTTTCCG
St-rngP-cat M R	GGTGGTATATCCAGTGATTTTTTTCTCATCAATGTACGCCACGCGCGTTTCCGA
St-rngP-cat M F	TCGGAAACGCGCGTGGCGTACATTGATGAGAAAAAAATCACTGGATATACCACC
**Construction of pSt-rncP-cat**	
St-rncP-cat F (NotI)	AAGCGGCCGCTTTCCTGGATAAATTCCCTA
St-rncP-cat M R	GGTGGTATATCCAGTGATTTTTTTCTCCTGATGATTAAAAGTGTAGCCCAGCTT
St-rncP-cat M F	AAGCTGGGCTACACTTTTAATCATCAGGAGAAAAAAATCACTGGATATACCACC
**Construction of pSt-hnsP-cat**	
St-hnsP-cat F (NotI)	AAGCGGCCGCTTTGAATTCCTTACATTCCT
St-hnsP-cat M R	CAGTGATTTTTTTCTCCATTTGCGGAGGGATTGAGGTAATAATAGAGCCTTAAATT
St-hnsP-cat M F	AATTTAAGGCTCTATTATTACCTCAATCCCTCCGCAAATGGAGAAAAAAATCACTG
**Construction of pTargetF (Amp)**	
amp F (XhoI)	CTACTCGAGTTCATGTGCAG
amp M R	CGGAAATGTTGAATACTCATGACATTGCACTCCACCGCTG
amp M F	CAGCGGTGGAGTGCAATGTCATGAGTATTCAACATTTCCG
amp R (MluI)	CTTTACGCGTTCGCGGAACTTCGGAATAGGAACTTATGAGCTCAGCCAATCGACTGGCGAG CGGCATCTTACCAATGCTTAATCAGTG
**Construction of pTargetF (Amp)-original N$_{20}$**	
pTargetF-hns-originalN20-SpeI-F	GGTATAATACTAGTAAAAGAGCTTATTGGCAGGGGTTTTAGAGCTAGAAATAGC

pTargetF-hns-originalN20-EcoRI-R	CGGAATTCAAAAAAAGCACCGACTCG
**Construction of pTargetF (Amp)-artificial N_{20}**	
pTargetF-hns-artificialN20-SpeI-F	GGTATAATACTAGTCAGCAACTCCAGGGGGCCGCGTTTTAGAGCTAGAAATAGC
**Construction of *hns* mutant strains**	
hns-original N20 F	AAAAGAGCTTATTGGCAGGG
artificial N20 F	CAGCAACTCCAGGGGGCCGC
hns a-N20 H F	GCAATAGCCAGGAATGTAAGGA
hns a-N20 M R	CGACAGACGGTGAGTATCCCGCGGCCCCCTGGAGTTGCTGTTGTGCGGTGCCTCAAGGAA
hns a-N20 M F	TTCCTTGAGGCACCGCACAACAGCAACTCCAGGGGGCCGCGGGATACTCACCGTCTGTCG
hns a-N20 H R	CTCATACCCGGTGCAAAGTAAT
St-hns (A-31C)-M-F	CTCTATTATTAGCTCAACAACCCACCCCAATATAAGTTTGA
St-hns (A-31C)-M-R	TCAAACTTATATTGGGGTGGGTTGTTGAGCTAATAATAGAG
St-hns (A-9G)-M-F	CACCCCAATATAAGTTTGAGGTTACTACAATGAGCGAAGCA
St-hns (A-9G)-M-R	TGCTTCGCTCATTGTAGTAACCTCAAACTTATATTGGGGTG
St-hns (T-8C)-M-F	ACCCCAATATAAGTTTGAGACTACTACAATGAGCGAAGCAC
St-hns (T-8C)-M-R	GTGCTTCGCTCATTGTAGTAGTCTCAAACTTATATTGGGGT
hns hR	AGCTTTGGTACCGGATTTAG
**For real-time qRT-PCR and RT-PCR**	
RT-St-hns-F (+149)	AAGTGGAAGAACGCACTCGT
RT-St-hns-R (+248)	TTAGCGGCAGCCATGCTATT
RT-St-rng (+1187)-F	GCGTGGAACATGTGCTTTGT
RT-St-rng (+1318)-R	AGCGATCGGAGTCATAAGCG
RT-St-sipA-F	GTCAGAAAAGGGCACTACGG
RT-St-sipA-R	CGGTCGTACCGGCTTTATTA
RT-St-hilA-F	CGTGAAGGGATTATCGCAGT
RT-St-hilA-R	AGAAGCGGGTTGGTGTTCTA
RT-St-sipC-F	CCATGGATATGACCCGAATC
RT-St-sipC-R	GCTAATTTGCTGCTCGGAAC
RT-St-ribE-F	TACGGGGATCGTACAGGGTA
RT-St-ribE-R	GCGCTCTACGTTGACCTCAT
16S-rRNA-1F	GAGTTTGATCATGGCTCAGA
16S-rRNA-300R	TCCAGTGTGGCTGATCATCC
CAT-RT F	AAAGACGGTGAGCTGGTGAT
CAT-RT R	GCCGGAAATCGTCGTGGTAT
**For northern blot analysis**	
St-hns (stop)-R	TTATTCCTTGATCAGGAAATC
M1-R	GCTCTCTGTTGCACTGGTCG
**For primer extension**	
St-hns-R (+30)	GTTGTTCAGAATTTTAAGTG

St-rng (+219)-R	AATGTCGGAAGCGTGAAGAAATGCG
**For cleavage assay**	
St-T7-hns-F	TAATACGACTCACTATAGGGAACAAACCACCCCAATATAAG
St-hns (stop)-R	TTATTCCTTGATCAGGAAATC

2
