# Supplementary material for: Endoribonuclease-mediated control of hns mRNA stability constitutes a key regulatory pathway for Salmonella Typhimurium pathogenicity island 1 expression
Source: PLoS Pathog. 2021 Feb 1;17(2):e1009263. doi: 10.1371/journal.ppat.1009263 (PMC7877770; doi:10.1371/journal.ppat.1009263)
Supplement: S5 Table — All statistical tests, biological replicates, exact P values, and significance for all graphs in this manuscript. (PDF) [file ppat.1009263.s014.pdf]

Table S5 Statistics and reproducibility.

All statistical tests, biological replicates, exact *P* values, and significance for all graphs in this manuscript.

**Figure**	**Test**	**Comparison**	***P* value**	**Significance**
1A	Multiple comparisons n=3 biologically independent experiments	WT	<.0001	a
		Δrng	<.0001	b
		Δrng^{comp}	<.0001	c
1B	Multiple comparisons n=3 biologically independent experiments	WT	0.0123	a
		Δrng	0.0123	b
		Δrng^{comp}	0.0123	a
1C	Unpaired Student *t*-test, two-sided n=10 mice per group	uninfected VS. WT	0.0024179406	**
		uninfected VS. Δrng	0.0279153996	*
		uninfected VS. Δrng^{comp}	0.0063403679	**
1D	Multiple comparisons n=5 mice per group biologically independent experiments	(Spleens) WT	<.0001	a
		(Spleens) Δrng	<.0001	b
		(Spleens) Δrng^{comp}	<.0001	a
		(Mesenteric lymph nodes) WT	<.0001	A
		(Mesenteric lymph nodes) Δrng	<.0001	B
		(Mesenteric lymph nodes) Δrng^{comp}	<.0001	A
		(Livers) WT	<.0001	α
		(Livers) Δrng	<.0001	β
		(Livers) Δrng^{comp}	<.0001	α
1E	Multiple comparisons n=5 mice per group biologically independent experiments	(% of CD11b+ cells) uninfected	<.0001	a
		(% of CD11b+ cells) WT	<.0001	b
		(% of CD11b+ cells) Δrng	<.0001	c
		(% of CD11b+ cells) Δrng^{comp}	<.0001	d
		(Number of CD11b+ cells) uninfected	<.0001	a
		(Number of CD11b+ cells) WT	<.0001	b
		(Number of CD11b+ cells) Δrng	<.0001	c
		(Number of CD11b+ cells) Δrng^{comp}	<.0001	b
1F	Multiple comparisons n=5 mice per group biologically independent experiments	(TNF-α) uninfected	<.0001	a
		(TNF-α) WT	<.0001	b
		(TNF-α) Δrng	<.0001	c
		(TNF-α) Δrng^{comp}	<.0001	b
		(IL-1α) uninfected	0.0031	a
		(IL-1α) WT	0.0031	b
		(IL-1α) Δrng	0.0031	a
		(IL-1α) Δrng^{comp}	0.0031	c
		(IL-6) uninfected	0.0017	a
		(IL-6) WT	0.0017	b
		(IL-6) Δrng	0.0017	a
		(IL-6) Δrng^{comp}	0.0017	b
		(IL-10) uninfected	0.0044	a
		(IL-10) WT	0.0044	b
		(IL-10) Δrng	0.0044	a
		(IL-10) Δrng^{comp}	0.0044	b
		(MCP-1) uninfected	<.0001	a
		(MCP-1) WT	<.0001	b
		(MCP-1) Δrng	<.0001	c
		(MCP-1) Δrng^{comp}	<.0001	d
2B	Unpaired Student *t*-test, two-sided n=3 biologically independent experiments	(3 h) FliC/FliD VS. SipC	0.9402135814	NS
		(3 h) FliC/FliD VS. SipA	0.8666289059	NS
		(3 h) FliC/FliD VS. SipB	0.2592797000	NS
		(3 h) FliC/FliD VS. FlgK	0.1502161172	NS
		(3 h) FliC/FliD VS. FlgL	0.0377868697	*
		(9 h) FliC/FliD VS. SipC	0.0013215199	**
		(9 h) FliC/FliD VS. SipA	0.0050205275	**

		(9 h) FliC/FliD VS. SipB	0.4232201229	NS
		(9 h) FliC/FliD VS. FlgK	0.4702085632	NS
		(9 h) FliC/FliD VS. FlgL	0.9353378975	NS
		(24 h) FliC/FliD VS. SipC	0.9330020322	NS
		(24 h) FliC/FliD VS. SipA	0.0062830456	**
		(24 h) FliC/FliD VS. SipB	0.3023721185	NS
		(24 h) FliC/FliD VS. FlgK	0.3170290629	NS
		(24 h) FliC/FliD VS. FlgL	0.3213470739	NS
3A	Unpaired Student *t*-test, two-sided n=3 biologically independent experiments	(*rng*) aerobic VS. anaerobic	5.131E-06	****
		(*hns*) aerobic VS. anaerobic	0.0157479285	*
		(*hilA*) aerobic VS. anaerobic	0.0026095803	**
		(*sipA*) aerobic VS. anaerobic	0.0003947900	***
		(*sipC*) aerobic VS. anaerobic	0.0003769326	***
3B	Unpaired Student *t*-test, two-sided n=3 biologically independent experiments	(Rng) aerobic VS. anaerobic	0.0391885332	*
		(H-NS) aerobic VS. anaerobic	0.0448323618	*
		(SipC) aerobic VS. anaerobic	0.0391871152	*
3C	Unpaired Student *t*-test, two-sided n=3 biologically independent experiments	(*rng*) 2 h VS. 3 h	0.0025663757	**
		(*rng*) 2 h VS. 5 h	0.0021901334	**
		(*hns*) 2 h VS. 3 h	0.0013046057	**
		(*hns*) 2 h VS. 5 h	0.0002302533	***
		(*hilA*) 2 h VS. 3 h	0.4898778592	NS
		(*hilA*) 2 h VS. 5 h	0.0015869642	**
		(*sipA*) 2 h VS. 3 h	0.1705842958	NS
		(*sipA*) 2 h VS. 5 h	0.0002717395	***
		(*sipC*) 2 h VS. 3 h	0.2311969041	NS
		(*sipC*) 2 h VS. 5 h	0.0041229997	**
4A	Unpaired Student *t*-test, two-sided n=3 biologically independent experiments	WT VS. Δrng	0.0179149608	*
		WT VS. Δrng^{comp}	0.0439913402	*
5A	Multiple comparisons n=3 biologically independent experiments	WT	0.0007	a
		hns (A-31C)	0.0007	b
		hns (A-9G)	0.0007	a
		hns (U-8C)	0.0007	b
5C	Unpaired Student *t*-test, two-sided n=15 mice per group	WT VS. hns (A-31C)	0.0021068	**
		WT VS. hns (A-9G)	0.62758914	NS
		WT VS. hns (U-8C)	7.9151E-05	****
5D	Multiple comparisons n=5 mice per group biologically independent experiments	(Spleen) WT	<.0001	a
		(Spleen) hns (A-31C)	<.0001	b
		(Spleen) hns (A-9G)	<.0001	c
		(Spleen) hns (U-8C)	<.0001	b
		(Mesenteric lymph nodes) WT	<.0001	A
		(Mesenteric lymph nodes) hns (A-31C)	<.0001	B
		(Mesenteric lymph nodes) hns (A-9G)	<.0001	C
		(Mesenteric lymph nodes) hns (U-8C)	<.0001	B
		(Livers) WT	<.0001	α
		(Livers) hns (A-31C)	<.0001	α
		(Livers) hns (A-9G)	<.0001	β
		(Livers) hns (U-8C)	<.0001	α
5E	Multiple comparisons n=3 mice per group biologically independent experiments	(*rng*) WT	0.7817	A
		(*rng*) hns (A-31C)	0.7817	A
		(*rng*) hns (A-9G)	0.7817	A
		(*rng*) hns (U-8C)	0.7817	A
		(*hns*) WT	0.0389	A
		(*hns*) hns (A-31C)	0.0389	B
		(*hns*) hns (A-9G)	0.0389	A
		(*hns*) hns (U-8C)	0.0389	B
		(*hilA*) WT	<.0001	A
		(*hilA*) hns (A-31C)	<.0001	B
		(*hilA*) hns (A-9G)	<.0001	C
		(*hilA*) hns (U-8C)	<.0001	B
		(*sipA*) WT	<.0001	A
		(*sipA*) hns (A-31C)	<.0001	B
		(*sipA*) hns (A-9G)	<.0001	A
		(*sipA*) hns (U-8C)	<.0001	B

		(*sipC*) WT<br>(*sipC*) hns (A-31C)<br>(*sipC*) hns (A-9G)<br>(*sipC*) hns (U-8C)	0.0191<br>0.0191<br>0.0191<br>0.0191	A<br>B<br>A<br>AB
6A	Multiple comparisons n=3 biologically independent experiments	(Rng) WT<br>(Rng) Δ*rng*<br>(Rng) Δ*rnc*	0.0002<br>0.0002<br>0.0002	a<br>b<br>c
		(Rnc) WT<br>(Rnc) Δ*rng*<br>(Rnc) Δ*rnc*	<.0001<br><.0001<br><.0001	A<br>A<br>B
		(H-NS) WT<br>(H-NS) Δ*rng*<br>(H-NS) Δ*rnc*	0.0139<br>0.0139<br>0.0139	αβ<br>α<br>β
6B	Unpaired Student *t*-test, two-sided n=3 biologically independent experiments	aerobic VS. anaerobic	0.0002928525	***
6C	Unpaired Student *t*-test, two-sided n=3 biologically independent experiments	aerobic VS. anaerobic	0.0230336734	*
6D	Unpaired Student *t*-test, two-sided n=10 biologically independent experiments	aerobic VS. anaerobic	0.8770621094	NS
6F	Unpaired Student *t*-test, two-sided n=3 biologically independent experiments	(*rngP*) aerobic VS. anaerobic	0.0174540948	*
		(*rncP*) aerobic VS. anaerobic	0.0005685293	***
		(*hnsP*) aerobic VS. anaerobic	0.6889200634	NS
6G	Multiple comparisons n=3 biologically independent experiments	(Rnc) (aerobic) WT<br>(Rnc) (aerobic) Δ*fnr*<br>(Rnc) (aerobic) Δ*arcA*<br>(Rnc) (anaerobic) WT<br>(Rnc) (anaerobic) Δ*fnr*<br>(Rnc) (anaerobic) Δ*arcA*	<.0001<br><.0001<br><.0001<br><.0001<br><.0001<br><.0001	a<br>a<br>a<br>b<br>c<br>b
		(Rng) (aerobic) WT<br>(Rng) (aerobic) Δ*fnr*<br>(Rng) (aerobic) Δ*arcA*<br>(Rng) (anaerobic) WT<br>(Rng) (anaerobic) Δ*fnr*<br>(Rng) (anaerobic) Δ*arcA*	0.0004<br>0.0004<br>0.0004<br>0.0004<br>0.0004<br>0.0004	A<br>A<br>A<br>B<br>A<br>B
		(H-NS) (aerobic) WT<br>(H-NS) (aerobic) Δ*fnr*<br>(H-NS) (aerobic) Δ*arcA*<br>(H-NS) (anaerobic) WT<br>(H-NS) (anaerobic) Δ*fnr*<br>(H-NS) (anaerobic) Δ*arcA*	0.0006<br>0.0006<br>0.0006<br>0.0006<br>0.0006<br>0.0006	α<br>α<br>α<br>β<br>α<br>β
S1	Unpaired Student *t*-test, two-sided n=5 mice per group	uninfected VS. 10^{2}<br>uninfected VS. 10^{3}<br>uninfected VS. 10^{4}<br>uninfected VS. 10^{5}	1<br>0.3370490580<br>0.0014965078<br>3.25E-8	NS<br>NS<br>**<br>****
S2	Multiple comparisons n=5 mice per group biologically independent experiments	(IL-1β) uninfected<br>(IL-1β) WT<br>(IL-1β) Δ*rng*<br>(IL-1β) Δ*rng*^{comp}	0.4112<br>0.4112<br>0.4112<br>0.4112	a<br>a<br>a<br>a
		(IL-12p70) uninfected<br>(IL-12p70) WT<br>(IL-12p70) Δ*rng*<br>(IL-12p70) Δ*rng*^{comp}	0.0655<br>0.0655<br>0.0655<br>0.0655	a<br>a<br>a<br>a
		(IL-17A) uninfected<br>(IL-17A) WT<br>(IL-17A) Δ*rng*<br>(IL-17A) Δ*rng*^{comp}	not determined<br>not determined<br>not determined<br>not determined	
		(IL-23) uninfected<br>(IL-23) WT<br>(IL-23) Δ*rng*<br>(IL-23) Δ*rng*^{comp}	not determined<br>not determined<br>not determined<br>not determined	
		(IL-27) uninfected	0.4112	a

		(IL-27) WT<br>(IL-27) Δ*rng*<br>(IL-27) Δ*rng*^{comp}	0.4112<br>0.4112<br>0.4112	a<br>a<br>a
		(IFN-β) uninfected<br>(IFN-β) WT<br>(IFN-β) Δ*rng*<br>(IFN-β) Δ*rng*^{comp}	0.2904<br>0.2904<br>0.2904<br>0.2904	a<br>a<br>a<br>a
		(GM-CSF) uninfected<br>(GM-CSF) WT<br>(GM-CSF) Δ*rng*<br>(GM-CSF) Δ*rng*^{comp}	0.3054<br>0.3054<br>0.3054<br>0.3054	a<br>a<br>a<br>a
S3A	n=3 biologically independent experiments			
S4A	Multiple comparisons n=3 biologically independent experiments	WT<br>A-31C<br>A-9G<br>U-8C	0.0038<br>0.0038<br>0.0038<br>0.0038	a<br>b<br>c<br>d
S4B	Unpaired Student *t*-test, two-sided n=3 biologically independent experiments	WT VS. A-31C<br>WT VS. A-9G<br>WT VS. U-8C	0.0254926797<br>0.0390064921<br>0.0289188726	*<br>*<br>*
S7	Multiple comparisons n=3 mice per group biologically independent experiments	(Spleens) (*rng*) WT<br>(Spleens) (*rng*) hns (A-31C)<br>(Spleens) (*rng*) hns (A-9G)<br>(Spleens) (*rng*) hns (U-8C)	0.3178<br>0.3178<br>0.3178<br>0.3178	a<br>a<br>a<br>a
		(Spleens) (*hns*) WT<br>(Spleens) (*hns*) hns (A-31C)<br>(Spleens) (*hns*) hns (A-9G)<br>(Spleens) (*hns*) hns (U-8C)	0.0314<br>0.0314<br>0.0314<br>0.0314	a<br>b<br>a<br>b
		(Spleens) (*hilA*) WT<br>(Spleens) (*hilA*) hns (A-31C)<br>(Spleens) (*hilA*) hns (A-9G)<br>(Spleens) (*hilA*) hns (U-8C)	<.0001<br><.0001<br><.0001<br><.0001	a<br>b<br>c<br>b
		(Spleens) (*sipA*) WT<br>(Spleens) (*sipA*) hns (A-31C)<br>(Spleens) (*sipA*) hns (A-9G)<br>(Spleens) (*sipA*) hns (U-8C)	0.0012<br>0.0012<br>0.0012<br>0.0012	a<br>b<br>a<br>b
		(Spleens) (*sipC*) WT<br>(Spleens) (*sipC*) hns (A-31C)<br>(Spleens) (*sipC*) hns (A-9G)<br>(Spleens) (*sipC*) hns (U-8C)	0.0401<br>0.0401<br>0.0401<br>0.0401	a<br>ab<br>a<br>b
		(Livers) (*rng*) WT<br>(Livers) (*rng*) hns (A-31C)<br>(Livers) (*rng*) hns (A-9G)<br>(Livers) (*rng*) hns (U-8C)	0.9479<br>0.9479<br>0.9479<br>0.9479	α<br>α<br>α<br>α
		(Livers) (*hns*) WT<br>(Livers) (*hns*) hns (A-31C)<br>(Livers) (*hns*) hns (A-9G)<br>(Livers) (*hns*) hns (U-8C)	0.0337<br>0.0337<br>0.0337<br>0.0337	α<br>β<br>α<br>β
		(Livers) (*hilA*) WT<br>(Livers) (*hilA*) hns (A-31C)<br>(Livers) (*hilA*) hns (A-9G)<br>(Livers) (*hilA*) hns (U-8C)	0.0442<br>0.0442<br>0.0442<br>0.0442	α<br>β<br>α<br>αβ
		(Livers) (*sipA*) WT<br>(Livers) (*sipA*) hns (A-31C)<br>(Livers) (*sipA*) hns (A-9G)<br>(Livers) (*sipA*) hns (U-8C)	<.0001<br><.0001<br><.0001<br><.0001	α<br>β<br>γ<br>β
		(Livers) (*sipC*) WT<br>(Livers) (*sipC*) hns (A-31C)<br>(Livers) (*sipC*) hns (A-9G)<br>(Livers) (*sipC*) hns (U-8C)	0.0409<br>0.0409<br>0.0409<br>0.0409	α<br>β<br>α<br>γ
